# Community-led strategies for communicable disease prevention and management in low- and middle- income countries: A mixed-methods systematic review of health, social, and economic impact

**DOI:** 10.1371/journal.pgph.0004304

**Published:** 2025-04-02

**Authors:** Pitchaya P. Indravudh, Kathleen McGee, Euphemia L. Sibanda, Elizabeth L. Corbett, Katherine Fielding, Fern Terris-Prestholt

**Affiliations:** 1 Department of Global Health and Development, London School of Hygiene & Tropical Medicine, London, United Kingdom; 2 Malawi-Liverpool-Wellcome Trust Clinical Research Programme, Blantyre, Malawi; 3 Centre for Sexual Health and HIV/AIDS Research, Harare, Zimbabwe; 4 Department of International Public Health, Liverpool School of Tropical Medicine, Liverpool, United Kingdom; 5 Department of Clinical Research, London School of Hygiene & Tropical Medicine, London, United Kingdom; 6 Department of Infectious Disease Epidemiology, London School of Hygiene & Tropical Medicine, London, United Kingdom; 7 School of Public Health, University of the Witwatersrand, Johannesburg, South Africa; 8 Joint United Nations Programme on HIV/AIDS, Geneva, Switzerland; University of California San Francisco, UNITED STATES OF AMERICA

## Abstract

Control of infectious diseases is a global health priority and a target of the 2015-2030 Sustainable Development Goals (SDGs). Community participation is fundamental to advancing primary health care and meeting SDGs. We conducted a mixed-methods systematic literature review of quantitative and qualitative evidence to understand the health, social, and economic impact of community-led strategies for communicable disease prevention and management in low- and middle- income countries. We searched seven electronic databases through 31 December, 2023 for cluster-randomised trials and economic evaluations of community-led communicable disease control. Reference searches identified additional studies associated with eligible database records. Data extraction and narrative synthesis summarised evidence on impact, costs, and cost-effectiveness, described the nature and extent of community participation, and examined implementation, mechanisms of impact, and contexts. Risk of bias of was assessed using the Cochrane Risk-of-Bias Tool and the Drummond checklist. Our database search yielded 14,269 records. Following database and reference screening, we included 49 records across 16 unique cluster-randomised trials, mostly from sub-Saharan Africa. Communicable disease strategies included provision of biomedical products, environmental modifications, and education and outreach. Based on evidence with moderate risk of bias, we found that community-led strategies can improve health behaviours for diarrhoeal diseases, HIV, malaria, and neglected tropical diseases. Evidence for impact on mortality and morbidity, health care access and utilisation, and community and social outcomes was less conclusive. Impact depended on the intensity of implementation by community actors. Factors facilitating implementation included motivation, trust, and health systems engagement. Contextual influences included attitudes and norms around communicable diseases. Economic studies were few and many omitted societal costs and consequences. This review supports community-led communicable disease control as a potentially effective strategy to improve health behaviours and contribute to SDGs. Operational guidance for implementation and evaluation is critical to support rapid evidence generation in this important area.

## Introduction

Control of infectious diseases is a global health priority and a target of the 2015-2030 Sustainable Development Goals (SDGs) [1]. Major communicable diseases, including HIV, tuberculosis, and malaria, are leading contributors to the global burden of disease, especially in low and middle- income countries [2]. While their impact on morbidity and mortality has been declining in recent decades, endemic and epidemic communicable diseases continue to pose significant threats to public health [2]. Advancement of primary health care is critical to universal health coverage and to meeting SDGs [3]. A fundamental component of primary health care is community participation [3].

Community-led strategies, which involve communities leading decision making and resource mobilisation in health programmes, have been advocated for decades but with limited implementation [4]. Responses that are driven by communities have potential to increase uptake and coverage of health programmes, improve health outcomes, and impact sustainability [5,6]. Empowerment of communities is suggested to enhance programme delivery through community-centred design and implementation and impact social determinants of health through power decentralisation, community systems strengthening, and collective engagement [7]. Calls for increased investment in community-led initiatives are based on the recognition that community participation is essential for meeting SDG targets [8]. Further, communicable diseases have spillover properties, making them amenable to a collective approach for prevention, screening and management, and surveillance [9].

There is an urgent need to consolidate evidence on community-led responses to support SDGs targeting communicable diseases. However, synthesising evidence on whether community participation improves health and, if so, through which mechanisms has been challenging [10,11]. Definitions of community participation are not standardised, leading to inconsistencies in their use and practice [12]. The scope of community participation is highly heterogeneous, and frameworks characterising participation lack agreement [13–19]. Further, community participation is a multicomponent process that interacts with many variables, including context, to improve outcomes. Complex interventions and systems can be difficult to capture through relatively simplified cause-effect frameworks [11], underscoring the importance of contextualising findings in evidence synthesis to identify common attributes across studies [20].

The main aim of this mixed-methods systematic literature review was to summarise and synthesise quantitative and qualitative evidence on community-led strategies for communicable disease prevention and management in low and middle- income countries. Previous reviews have examined community participation more broadly [10,21–24] or have been disease specific [25–27]. The novel aspects of this review were that we aimed to focus on community-led studies and to assess evidence across a range of diseases and disease syndemics [28]. The specific objectives were to: (i) summarise the impact, costs, and cost-effectiveness of community-led approaches, (ii) describe the nature and extent of community participation, and (iii) examine the mechanisms through which community-led approaches affect outcomes and their interactions with contexts.

## Methods

The review was registered with PROSPERO (CRD42021281164) and followed the Cochrane handbook for systematic reviews and PRISMA guidelines ( S1 PRISMA Checklist) [29,30].

### Defining ‘community-led’

UNAIDS defines community-led responses as “actions and strategies that…are specifically informed and implemented by and for communities and the organisations, groups, and networks that represent them” [31]. However, definitions and applications of ‘community’ and ‘participation’ have varied widely in public health [12]. Community refers to a group of people with shared spatial or social characteristics or collective interests [32]. Community participation encapsulates a continuum of increasing empowerment, as outlined by frameworks summarised in Text A in S1 Text. These frameworks characterise the nature and extent of participation by external actors (e.g., governmental and non-governmental organisations) and community actors in health programmes. At the lowest end of the continuum, health is defined as the absence of disease [14]; external actors are perceived as experts who are best positioned to identify health problems and solutions, with the community acting as a setting or target of externally prescribed agendas [13–17,19]. The highest end defines health broadly as the human condition [14]; the community is an agent for change, supported by external actors to prioritise and solve health problems [13–17,19]. Community-led responses, which have adopted a range of terminology, are founded on principles of empowerment [31,33–35].

### Eligibility criteria

**Fig 1:**
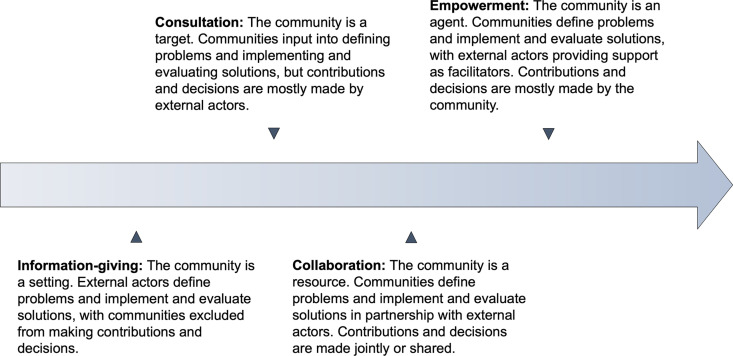
Framework for community participation. Continuum of community participation indicating an increasing degree of empowerment. Adapted from Rifkin and Pridmore (2001) and Draper (2010) [14,18].

Eligibility criteria included cluster-randomised trials, economic evaluations, and process evaluations in low-and-middle-income countries that compared community-led strategies for communicable disease control against any alternatives, including facility- and community-based alternatives (Text B in S1 Text). Studies comparing alternative community-led strategies were also eligible. Interventions qualified as community-led if (i) the intervention was delivered outside of standard health facilities, (ii) the intervention involved community participation in the delivery of the intervention, and (iii) the extent of community participation involved making significant contributions and decisions for at least one stage of the intervention (design, implementation, monitoring and evaluation, or post-implementation). The framework used to define and categorise interventions for the last criterion is summarised in Fig 1 and mainly adapted from Rifkin and Pridmore (2001) and Draper (2010) [14,18]. Using this framework, we included studies where at least one stage of the intervention was classified as ‘empowerment’ and excluded studies where all stages of the intervention were classified as ‘information-giving’, ‘consultation’, or ‘collaboration’.

Studies were also included if at least one outcome was related to communicable diseases. Outcomes included morbidity and mortality, health care access and utilisation, health behaviours, community and social outcomes, environmental outcomes, and costs and cost-effectiveness. Additional criteria were studies published in peer-reviewed journals and in English, with no limitations on date of publication.

### Search strategy, screening, and data extraction

We searched seven electronic databases (Cochrane Trials, Econlit, Embase, Global Health, Medline, Pubmed, Web of Science) on 11 October, 2021, updated through 31 December, 2023. Searches were based on terms for community-led strategies and communicable diseases, as described in Text B in S1 Text. References from eligible studies were also screened. Database searches were calibrated to yield randomised trials and related studies, including economic evaluations. Reference searches aimed to identify additional studies, including process evaluations, associated with eligible records from database searches. Following automated removal of duplicates, PPI screened titles and abstracts for initial inclusion and PPI and KM independently reviewed full texts for final inclusion, with disagreements resolved by consensus.

PPI extracted data using standardised forms on study characteristics; intervention and comparator characteristics, including the nature and extent of community participation; results on effects, costs, and cost-effectiveness; results on implementation, mechanisms of impact, and contexts; and details for quality appraisal (Text C in S1 Text). Estimates were extracted for all outcomes and time points from adjusted analyses, if reported. Results from subgroup analysis were extracted if outcomes were only assessed for subgroups. Risk of bias, including from missing data, was assessed using the Revised Cochrane Risk of Bias Tool for Randomised Trials and the Drummond checklist [36,37]. Certainty of evidence and risk of publication bias were not assessed due to heterogeneity in outcomes. To evaluate consistency, KM independently extracted data and conducted quality appraisal for a random sample of records as well as records that included authors of the current review.

### Data synthesis

We followed narrative reporting based on synthesis without meta-analysis guidelines, since meta-analysis was not appropriate given variation in outcomes [38]. All included studies were eligible for synthesis and are described with their risk of bias, if relevant. Narrative reporting on effectiveness was grouped by disease area and outcome domain, which included mortality and morbidity, health care access and utilisation, health behaviours, community and social outcomes, and environmental outcomes. Narrative reporting prioritised primary outcomes as well as secondary outcomes more relevant to communicable diseases and their determinants. We also aimed to identify common attributes and themes and draw conclusions by disease area and outcome domain.

Synthesis addressed each of our objectives. We summarised the direction of effect from outcomes reported in cluster-randomised trials and used harvest plots to present summaries by disease area, outcome domain, and level of community participation [39]. Cost and cost-effectiveness estimates were standardised to 2023 US Dollars [40] and summarised. To measure community participation, we categorised interventions into domains using a scoring method [14,18,21,23] from 0 to 4 (0=no information, 1=information giving, 2=consultation, 3=collaboration, 4=empowerment) that was applied to design, implementation, monitoring and evaluation, and post-implementation stages (Text D in S1 Text). Overall scores ranged from 0 to 16, indicating low to high levels of community participation. Radar graphs were used to illustrate scores. Finally, we mapped evidence on implementation, mechanisms of impact, and contexts based on the Medical Research Council process evaluation framework [41]. Specifically, we synthesised what was implemented and how; how the intervention changed outcomes; and how factors external to the intervention affected implementation and outcomes [41]. Analysis used a results-based convergent synthesis approach, whereby quantitative and qualitative data for each process evaluation function were extracted and analysed separately, compared in a thematic matrix, and interpreted to consider all evidence [42].

## Results

**Fig 2:**
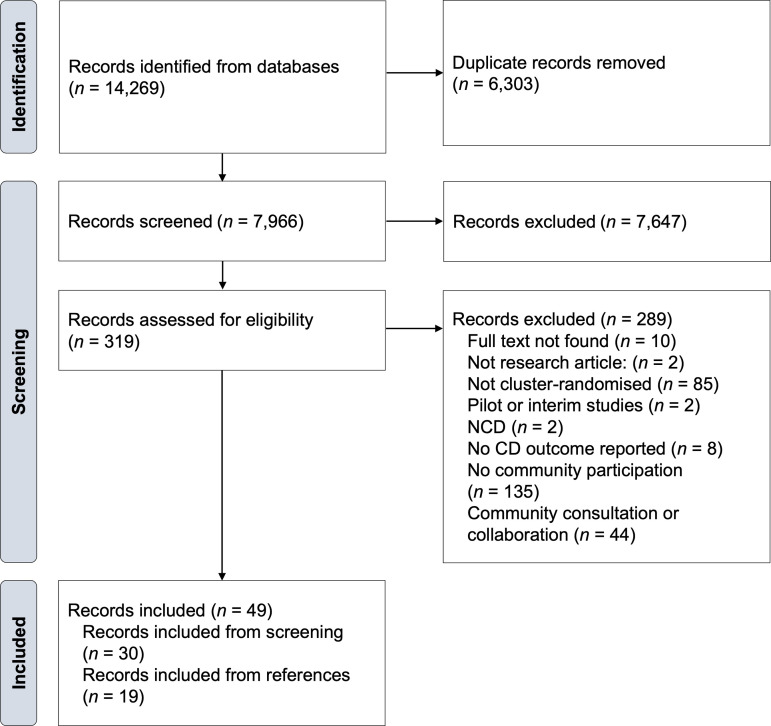
Flow diagram. CD, communicable disease; NCD, non-communicable disease. Flow diagram of record identification, screening, and inclusion.

Our database search strategy yielded 14,269 records (Fig 2). After removing duplicate articles, we screened titles and abstracts of 7,966 records, of which 319 records were eligible for full-text review. We included 30 records and identified an additional 19 records from reference searches. Overall, we included 49 records across 16 unique cluster-randomised trials [43–58]. That is, each trial had multiple records, with 30 records reporting on impact outcomes [43–72]; 12 records reporting on economic outcomes (eight economic evaluations, four costing studies) [51,52,55,58,73–80]; and 26 records reporting on process outcomes (15 quantitative studies, eight qualitative studies, three mixed-methods studies) [45,47,49–52,55,56,58–61,64–66,81–91]. Table 1 describes the characteristics and main results of included cluster-randomised trials and lists their substudies.

**Table 1:** Study characteristics of cluster-randomised trials.

						Community participation score					
Article	Study design	Setting	Population	Intervention	Control	Design	Implementation	M&E	Post-implementation	Overall	Main results
**Diarrhoeal diseases**											
Biran (2018) [45]	CRT of group village head units	Malawi	People with disabilities	CLTS inclusive of people with disabilities. External actors and health surveillance assistants facilitated ‘triggering’ exercises (e.g., community mapping, action planning), aiming to include people with disabilities. Sanitation committees led improved sanitation activities inclusive of people with disabilities in their villages, with some monitoring from external actors and community health workers.	CLTS. External actors and health surveillance assistants facilitated ‘triggering’ exercises (e.g., community mapping, action planning). Sanitation committees led improved sanitation activities in their villages, with some monitoring from external actors and community health workers.	3	4	3	0	10	No difference between arms in primary outcome of latrine construction. No differences between arms in sanitation outcomes, including improved latrine access and use.
Briceño (2017) [46] **Related studies** ^‡^ Briceño (2015) [73]	Factorial CRT of wards	Tanzania	General population (adults and children)	CLTS. External actors, including district and ward officers, facilitated ‘triggering’ exercises (e.g., community mapping, action planning), trained local masons, and conducted mass media activities. Sanitation committees led and monitored improved sanitation activities in their villages. CLTS and handwashing promotion.	Handwashing promotion. Trained community activists provided handwashing promotion activities to households alongside mass media activities and infrastructure building. No intervention.	3	4	4	0	11	No differences in primary outcome of 7-day child diarrhoeal prevalence between both intervention arms vs control arm. Lower 14-day diarrhoeal prevalence, haemoglobin levels, and weight-for-age among children in CLTS and handwashing promotion arm vs. control arm. No differences in morbidity outcomes between CLTS arm vs. control arm. Higher coverage of most sanitation outcomes, including improved latrine access and use and absence of open defection, in both intervention arms vs. control arm.
Cameron (2019) [47] Related studies ^*^ Borja-Vega (2014) [82]	CRT of villages	Indonesia	General population (adults and children)	CLTS. External actors, including government officers, facilitated ‘triggering’ exercises, including community mapping and action planning. Community members led improved sanitation activities in their villages, with some monitoring from external actors.	No intervention.	3	4	3	0	10	Lower roundworm density among children in intervention arm vs. control arm. No differences in haemoglobin levels, height, weight, and health index. Higher latrine construction and absence of open defection in intervention arm vs. control arm. No difference in diarrhoeal knowledge.
Cha (2021) [48] **Related studies** ^‡^ Cha (2020) [74]	CRT of villages	Ethiopia	General population (adults and children)	CLTS. External actors, including district health officers, health care providers, and health extension workers, facilitated ‘triggering’ exercises (e.g., community mapping) and identified and trained WASH promoters on sanitation management. WASH promoters led improved sanitation activities in their villages, with monitoring from external actors, and received compensation.	SOC. Government sanitation services provided by health extension workers.	3	4	3	0	10	For primary outcomes, lower diarrhoeal incidence and 100-day diarrhoeal prevalence among children in intervention arm vs. control arm. No differences in diarrhoeal duration and 7-day diarrhoeal prevalence. Higher coverage of most sanitation outcomes, including improved latrine access, in intervention arm vs. control arm. High probability of cost-effectiveness.
Crocker (2016) [49] Related studies ^*^ Crocker (2017) [63] ^‡^ Crocker (2017) [75] ^‡^ Crocker (2021) [76]	CRT of villages	Ghana	General population (adults and children)	CLTS with training of natural leaders. External actors facilitated ‘triggering’ exercises (e.g., community mapping) and trained natural leaders, including on action planning. Natural leaders led improved sanitation activities in their villages.	CLTS. External actors facilitated ‘triggering’ exercises, including community mapping. Community members led improved sanitation activities in their villages.	3	4	0	0	7	Higher improved latrine access and use and lower open defecation in intervention arm vs. control arm.
Pickering (2015) [56]	CRT of villages	Mali	General population (adults and children)	CLTS. External actors, including Sanitation Department officers, facilitated ‘triggering’ exercises, including community mapping. Sanitation committees led improved sanitation activities in their villages, with monitoring from external actors.	No intervention.	3	4	3	0	10	For primary outcomes, no differences between arms in 2-day diarrhoeal prevalence and 2-week diarrhoeal prevalence among children. Differences between arms in most development outcomes among children, including height-for age, weight-for-age, stunting, and wasting. Lower diarrhoea-related mortality in intervention arm vs. control arm. No differences in diarrhoeal symptoms. Differences between arms in most sanitation outcomes, including latrine access and use and open defecation. No difference between arms in E. coli level.
Quattrochi (2018) [57] Related studies ^*^ Crocke (2022) [64]	CRT of village groups	Democratic Republic of Congo	General population (adults and children)	Community-led WASH. External actors, including Health Zone officers, trained WASH committees, including on problem solving and assessment, action planning, and WASH management. WASH committees and volunteers decided on and led improved WASH activities in their villages, with monitoring from Health Zone officers, and received compensation and material support.	No intervention.	4	4	3	3	14	For primary outcomes, higher coverage of improved water and sanitation infrastructure in intervention arm vs. control arm. No differences in water access and availability. No differences between arms in child diarrhoea symptoms and health care use. Higher coverage of WASH outcomes, including improved water and sanitation indices, in intervention arm vs. control arm.
**HIV**											
Abramsky (2014) [59] Related studies ^*^^,^^†^ Kyegombe (2014) [85] ^†^ Kyegombe (2014) [86] ^*^ Abramsky (2016) [43] ^*^ Abramsky (2016) [60] ^‡^ Michaels-Igbokwe (2016) [78] ^†^ Starmann (2017) [90] ^*^ Abramsky (2018) [81] ^*^^,^^†^ Starmann (2018) [91]	Pair-matched CRT of administrative parishes	Uganda	General population (adults)	Community mobilisation for HIV and IPV prevention. External actors facilitated a four-phase cycle (start, awareness, support, and action), which included training of community activists on strategies for local activism. Community activists led mobilisation activities, with support from external actors. Mass media and advocacy, communications, and ongoing trainings and mentoring to community activists, leaders, and stakeholders were also provided by external actors.	Enhanced SOC. Community activists provided with basic health training.	4	4	0	3	11	For primary outcomes, higher acceptance of refusal to have sex among women and men in intervention arm vs. control arm. Lower acceptance of physical IPV among women and concurrency of sexual partners among men. No differences in physical IPV, sexual IPV, acceptance of physical IPV among men, and community response to IPV. Higher HIV testing among men in intervention arm vs. control arm. Differences between arms in some IPV outcomes, including emotional IPV, and outcomes on gender attitudes and norms, including gender roles.
Indravudh (2021) [50] **Related studies** ^‡^ Indravudh (2021) [77] ^*^ Indravudh (2022) [66]	CRT of group village head units	Malawi	General population (adults)	Community-led HIVST. External actors trained community groups and volunteers, including on problem solving and assessment, action planning, and HIVST. Community groups and volunteers decided on, led, and monitored HIVST activities in their villages and received compensation and material support.	SOC. Standard HIV testing services provided through government health facilities.	4	4	4	0	12	For primary outcome, higher lifetime HIV testing among adolescents in intervention arm vs. control arm. Higher HIV testing among adults ≥40 years and HIV testing among men in intervention arm vs. control arm. No difference in antiretroviral therapy initiation. Higher social cohesion and shared concern for HIV in intervention arm vs. control arm. No differences in knowledge of HIV treatment benefits, HIV testing stigma, community HIV stigma, and critical consciousness. Low to moderate probability of cost-effectiveness
Sibanda (2021) [58] Related studies ^*^ Thomas (2023) [72]	CRT of village headman units	Zimbabwe	General population (adults)	Community-led HIVST. External actors engaged community leaders and members, who decided on HIVST activities. Community distributors received trainings and led HIVST activities in their villages, with monitoring from health facilities, and received material support.	Community-based HIVST. Trained community distributors implemented door-to-door HIVST delivery.	4	4	3	0	11	No differences between arms in primary outcomes of new HIV diagnosis and linkage to confirmatory HIV testing and prevention.
**Malaria**											
McCann (2021) [54] Related studies ^*^^,^^†^ Malenga (2017) [87] ^†^ Kaunda-Khangamwa (2019) [84] ^†^ Gowelo (2020) [83] ^‡^ Phiri (2021) [79] ^*^ Gowelo (2023) [65]	Factorial CRT of village groups	Malawi	General population (adults and children)	Community-driven larval source management. External actors trained village committees and health animators on malaria control. Village committees and health animators led and monitored activities for larval source management activities in their villages. Community-driven house improvement. External actors trained village committees and health animators on malaria control. Village committees and health animators led and monitored activities for house improvement activities in their villages. Community-driven larval source management and house improvement.	SOC. Government malaria control programmes.	3	4	3	0	10	No difference in primary outcome of entomological inoculation rate between intervention arms vs. control arm. No differences in malaria prevalence and haemoglobin levels between intervention arms vs. control arm. Almost no differences in mosquito densities between intervention arms vs. control arm.
**Neglected tropical diseases**											
Andersson (2015) [44] Related studies ^*^ Carcamo (2017) [62] ^*^ Jimenez-Alejo (2017) [67] ^*^ Legorreta-Soberanis (2017) [68] ^*^ Legorreta-Soberanis (2017) [69] ^*^ Legorreta-Soberanis (2017) [70] ^*^ Alvarado-Castro (2019) [61] ^‡^ Tschampl (2020) [80]	CRT of census enumeration areas	Mexico, Nicaragua	General population (adults and children)	Community-led dengue control. External actors trained community groups and volunteers on dengue control. Community groups and volunteers decided on and led dengue control activities at community level, with monitoring from neighbouring peers. Community volunteers also implemented household activities.	SOC. Government dengue control programmes, including distribution of temephos sachets and space spraying.	4	4	3	0	11	For primary outcome, lower dengue infection in intervention arm vs. control arm. Differences between arms in some dengue control outcomes, including pesticide use. Almost no differences in community attitudes and norms on dengue control. Lower larvae and pupae density in intervention arm vs. control arm. Low to moderate probability of cost-effectiveness
Massa (2009) [53] Related studies ^*^ Massa (2009) [71] ^†^ Massa (2009) [88]	CRT of school catchment areas	Tanzania	General population (children)	Community-directed distribution of treatment for schistosomiasis and soil-transmitted helminthiasis. External actors engaged community leaders and members, who decided on parasitological treatment activities. Community drug distributors received trainings and led parasitological treatment activities in their villages and received material support.	School-based treatment for schistosomiasis and soil-transmitted helminthiasis. Teachers distributed drugs to school-age children.	4	4	0	0	8	For primary outcomes, some differences between arms in parasitological prevalence outcomes. Some differences between arms in treatment coverage outcomes.
**Multiple diseases**											
Lewycka (2013) [51] **Related studies** ^†^ Rosato (2012) [89]	Factorial CRT of census enumeration areas	Malawi	General population (women and children)	Participatory women’s groups for maternal and child health, including for HIV, malaria, and immunisation. External actors and community facilitators guided a four-phase cycle (identifying and prioritising problems, planning, implementation, and evaluation). Women’s groups prioritised problems and decided on, led, and evaluated maternal and child health activities. Participatory women’s groups and peer counselling.	Peer counselling for pregnant women. Trained peer counsellors provided health education to pregnant women through scheduled antenatal and postnatal visits at home. Enhanced SOC. Standard services provided through government health facilities, with health systems strengthening.	4	4	4	4	16	No differences in primary outcomes of maternal, perinatal, neonatal, and infant mortality rates between intervention arms vs. control arm. Some differences in outcomes on access of antenatal and infant care, including infant immunisation, between intervention arms vs. control arm. Almost no differences in use of insecticide treated bed nets and breastfeeding practices between intervention arms vs. control arm.
Makaula (2019) [52]	CRT of health facility catchment areas	Malawi	General population (adults and children)	Community-directed primary health care. External actors, including health care providers, engaged community members, who decided on primary health care activities. Community volunteers received trainings and led primary health care activities in their villages, with monitoring from external actors.	SOC. Standard services provided through government health facilities.	4	4	3	0	11	No differences between arms in use of Antimalarial drugs, vitamin A, and praziquantel. Higher use of long-lasting insecticide treated bed nets among women and children in intervention arm vs. control arm.
Nair (2017) [55]	CRT of villages and adjoining hamlets	India	General population (women and children)	Participatory women’s groups for maternal and child health, including for immunisation. External actors and community-based workers guided a four-phase cycle (identifying and prioritising problems, planning, implementation, and evaluation). Women’s groups prioritised problems and decided on, led, and evaluated maternal and child health activities.	Enhanced SOC. Capacity strengthening of village health and sanitation committees and standard government health services.	4	4	4	0	12	For primary outcome, lower child length-for-age in intervention arm vs. control arm. Almost no differences between arms in infant mortality and child development outcomes, including wasting and stunting. No differences in infant care outcomes, including immunisation. Differences between arms in most outcomes on child nutrition and hygiene, including handwashing.

ART, antiretroviral therapy; CLTS, community-led total sanitation; CRT, cluster randomised trial; HIVST, HIV self-testing; IPV, intimate partner violence; SOC, standard of care; WASH, water, sanitation, and hygiene. Scores use a 0–4 scale: 0=not reported, 1=information giving, 2=consultation, 3=collaboration, 4=empowerment.

* Quantitative studies.

† Qualitative studies.

‡ Economic studies.

### Characteristics of included studies

Disease areas were diarrhoeal diseases, HIV, malaria, and neglected tropical diseases, with three cluster-randomised trials including strategies targeting multiple diseases [51,52,55]. Most trials were in sub-Saharan Africa, with 10 trials in Eastern and Southern Africa [45,46,48,50–54,58,59] and three trials in Western and Central Africa [49,56,57]. All trials were directed towards the general population, except for one trial, which focused on people with disabilities [45]. In all trials, ‘community’ was defined geographically (Table A in S1 Text). Strategies for engaging community actors were varied and included problem solving and assessment, action planning, skills development, and goal setting and review. Communicable disease strategies included provision of biomedical products, environmental modifications, or education and outreach. Periods of implementation spanned from 2 weeks to 4 years. Overall scores for community participation had a mean of 10.9 out of 16, indicating upper-moderate levels of participation. Scores were highest for the implementation stage and lowest for the post-implementation stage (Fig A in S1 Text).

Of the 16 trials, one study had low risk of bias and 10 studies had moderate risk of bias (Table B in S1 Text). Five studies were found to have high risk of bias, mostly related to their reporting of missing outcome data. Among the eight economic evaluations, all except one study reported high risk of bias (Table C in S1 Text), with the most common reason being exclusion of important costs and consequences, namely societal.

### Impact

**Fig 3:**
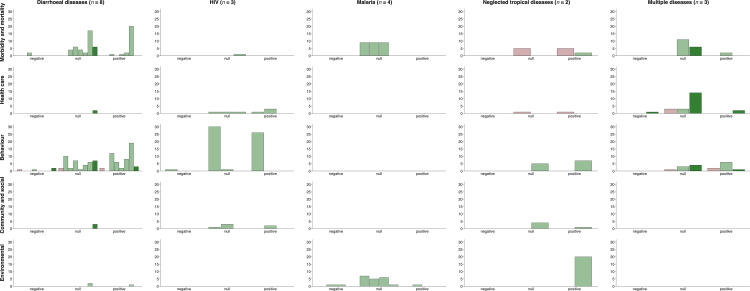
Evidence of intervention effects by disease area. Harvest plot with outcome domains indicated in rows and disease areas indicated in columns. Each bar represents a single study, or if a single study included more than two comparison arms, each bar represents a single comparison (*N* = 20). The height of the bar represents the number of outcomes within that study that reported a positive, negative, or null effect, which was determined based on the effect estimate and confidence interval for each outcome. A positive effect indicates a favourable outcome, while a negative effect indicates an adverse outcome. The colour of each bar represents community participation scores ranging from 1-16: dark red for low scores of 1-4, light red for lower-moderate scores of 5-8, light green for upper-moderate scores of 9-12, and dark green for high scores of 13-16.

Table D in S1 Text summarises intervention effects for each cluster-randomised trial. Most studies evaluated outcomes related to health, health care access and utilisation, and health behaviours, while few studies assessed community and social outcomes. Some studies also assessed environmental outcomes, such as parasitological and entomological indices. Fig 3 includes a harvest plot that illustrates the direction of intervention effects by disease area, outcome domain, and community participation domain. Each bar represents a single study, with its height indicating the number of outcomes reporting a positive or favourable effect, negative or adverse effect, or null effect. The colour of each bar represents community participation scores. For example, Abramsky et al., has a light green bar, indicating an upper-moderate community participation score, and reported intervention effects for 60 outcomes, of which 27 were positive, 1 was negative, and 32 were null. Overall, the figure showed no observable patterns on the direction of effect by disease area, outcome domain, and level of community participation.

#### Diarrhoeal diseases.

Seven cluster-randomised trials targeted diarrhoeal diseases, mainly through community-led total sanitation (CLTS) [45–49,56,63]. CLTS involved external actors initiating a situational assessment or ‘triggering’ with community actors, who subsequently devised and enacted action plans to meet goals for improved sanitation. The implementation period ranged from less than 1 year to 2 years. Communicable disease strategies, such as latrine construction, were often predefined by external actors. Levels of community participation varied from lower-moderate to upper-moderate. Another trial evaluated a community-driven water, sanitation, and hygiene (WASH) strategy across 6 months in the Democratic Republic of Congo [57,64]. Administrative health zones facilitated problem assessment and solving. Village committees then led the design and implementation of action plans and collaborated with health zones on monitoring and evaluation and post-implementation, achieving a high community participation score.

Three trials, which had moderate risk of bias, evaluated the impact of CLTS on child diarrhoeal prevalence and incidence compared with the standard of care (SOC) [46,48,56]. In Ethiopia, Cha et al. observed moderate evidence of a decrease in primary outcomes of diarrhoeal incidence (adjusted incidence ratio 0.66, 95% CI 0.45 to 0.97) and 100-day diarrhoeal prevalence (adjusted prevalence ratio 0.70, 95% CI 0.52 to 0.95) [48]. A factorial trial in Tanzania reported weak evidence of reductions in diarrhoeal prevalence at 14-days when CLTS was combined with handwashing promotion, with no differences measured for other diarrhoea and child development outcomes [46]. No differences in morbidity outcomes were observed when evaluating CLTS alone against the SOC. A Malian trial of CLTS found no evidence of changes in diarrhoeal prevalence but reported statistically significant evidence of improvements in outcomes for diarrhoea-related mortality and child development [56]. In Indonesia, a trial of CLTS found weak evidence of reductions in roundworm infection but found no differences in child development outcomes, though high risk of bias was identified [47]. Impact on child health from community-driven WASH was also not found, with moderate risk of bias reported [57,64].

Most trials observed improvements in terms of preventive health behaviours. Positive changes were reported for sanitation practices, including improved latrine access and use and open defecation [46–49,56,57]. There was strong evidence of an increase in ownership of improved latrines following the introduction of CLTS in Ethiopia [48]. A trial with moderate risk of bias also reported strong evidence of changes in improved latrine ownership and open defecation when training of opinion leaders was added to CLTS in Ghana [49]. Improvements in water and hygiene practices, such as handwashing, were also reported [46,48,56,57]. An exception was a trial in Malawi that compared CLTS inclusive of people with disabilities against standard CLTS [45]. The study did not report an effect on any WASH behaviours, citing poor engagement of people with disabilities as a target population, though high risk of bias was detected.

#### HIV.

Three cluster-randomised trials focused on HIV, with all reporting moderate risk of bias [43,50,58,59,66,85]. Two trials evaluated community-led diagnostic testing, which both had upper-moderate community participation scores. In Malawi, community groups and volunteers participated in workshops and trainings to prepare for 7-day HIV self-testing (HIVST) campaigns [50,66]. Provision of HIVST was fixed by external actors, but approaches for design, implementation, and monitoring and evaluation were decided on by community actors. Compared with the SOC, the study reported strong evidence of improved HIV testing coverage, including a 15.2% (95% CI 7.5% to 22.9%) increase in lifetime HIV testing among adolescents [50]. The study also reported weak evidence of an intervention effect on social cohesion and collective HIV concern [66]. A Zimbabwean trial evaluated 6-week community-led HIVST campaigns by unpaid community volunteers, who led on design and implementation and collaborated on monitoring and evaluation. Compared with community-based implementation by externally supported and paid distributors, the trial did not report an intervention effect on new HIV diagnosis and linkage to HIV prevention and care [58].

Another trial in Uganda assessed the impact of community mobilisation for HIV and intimate partner violence (IPV) prevention against the SOC [43,59,85]. The intervention showed upper-moderate levels of community participation, with groups of community activists leading design and implementation of education and outreach activities across 4 years. Mobilisation was done in tandem with externally planned activities, including mass media and health and social systems strengthening [59]. Monitoring and evaluation were also done in collaboration with external actors. The study reported statistically significant improvements in HIV testing for men but not women [85]. In terms of behavioural outcomes, the study reported no differences between arms in the primary outcomes of physical and sexual IPV, but did detect statistically significant reductions in other forms of IPV as well as changes in gender roles and norms, interpersonal dynamics, and HIV risk behaviours, including partner concurrency and condom use among men [43,59,85].

#### Malaria.

McCann et al. conducted a factorial cluster-randomised trial comparing community-driven strategies for larval source management and house improvements with the SOC, with the study showing high risk of bias [54,65]. The intervention had upper-moderate levels of community participation. For two years, village committees and health animators led community workshops and implementation of externally designed vector control activities. Monitoring and evaluation were done in collaboration with government community health workers. For the primary outcome of entomological inoculation rate and most secondary outcomes, including malaria prevalence, the study did not demonstrate evidence of an effect for any of the interventions.

#### Neglected tropical diseases.

Two cluster-randomised trials evaluated strategies for neglected tropical diseases [44,53,61,62,67–71]. Andersson et al. conducted a trial of community-led strategies for dengue control compared with the SOC in Mexico and Nicaragua, with low risk of bias found [44,61,62,67–70]. The intervention demonstrated upper-moderate levels of community participation, with community groups and volunteers leading the design and implementation of community-wide education and outreach activities across 1 year. Volunteers also conducted household education, as determined by external actors. The study reported moderate to weak evidence of changes in the primary outcomes, including dengue infection (relative risk reduction 29.5%, 95% CI 3.8% to 55.3%) and dengue-related vectors [44,61,62,67]. Preventive health behaviours, including knowledge and practice of dengue control, also improved based on moderate to weak evidence [44,68]. Changes in community-level outcomes, such as collective action and social capital, were not detected [61].

Massa et al. compared community-directed distribution of treatment for schistosomiasis and soil-transmitted helminthiasis, which had lower-moderate levels of community participation, with school-based delivery in Tanzania [53,71]. Community leaders and members decided on distribution activities in their villages and elected drug distributors, who led implementation across 1 year. The trial, which had high risk of bias, found strong to moderate evidence of reductions in some parasitological outcomes and improvements in treatment coverage.

#### Multiple diseases.

Two cluster-randomised trials evaluated participatory women’s groups for maternal and child health, which had upper-moderate and high levels of community participation [51,55]. Women’s groups were guided through a participatory learning and action cycle where they prioritised disease areas, decided on actions to address health priorities, and implemented and evaluated identified actions. In Malawi, a factorial trial compared 3-year participatory women’s groups with peer counselling for pregnant women and an enhanced SOC, with moderate risk of bias reported [51]. The study did not observe evidence of an intervention effect on the primary outcomes of maternal and infant mortality [51]. In India, a trial with moderate risk of bias compared participatory women’s groups against an enhanced SOC. The trial found weak evidence of improvements in the primary outcome of infant length-for-age (adjusted mean difference 0.107, 95% CI -0.011 to 0.226) but no changes in other child development outcomes [55].

In terms of health care access and utilisation, the Malawian study reported a statistically significant increase in uptake of infant immunisation, but no changes in other outcomes including HIV testing at antenatal care [51]. The study in India showed strong evidence of changes in WASH behaviours for infants [55]. Makaula et al. evaluated provision of community-directed primary care, which had upper-middle levels of community participation, compared with the SOC in Malawi [52]. The trial, which had high risk of bias, did not report differences in treatment uptake for malaria and schistosomiasis but found strong evidence of an increase in use of insecticide treated bed nets in women and children.

### Costs and cost-effectiveness

Tables E and F in S1 Text summarise estimates for costs and cost-effectiveness for each cluster-randomised trial. Seven of eight economic evaluations were trial based. All studies measured full economic costs, with seven studies adopting a provider perspective [51,52,55,58,77,78,80] and five studies adopting a societal perspective [73–76,79]. Community costs, including valuation of community time use and in-kind contributions, were captured in most studies, though were often incomplete in measurement.

Using cost-benefit analysis, Cha et al. reported that provision of CLTS yielded net societal benefits against the SOC in Ethiopia, with moderate risk of bias identified [74]. Benefits, which were valued based on premature diarrhoeal deaths and illness from diarrhoea cases averted, substantially outweighed costs over a 10-year period, including across different levels of uncertainty. Two trial-based economic evaluations of CLTS, which had high risk of bias, were also conducted from a societal perspective [73,75,76]. Crocker et al. evaluated the addition of opinion leaders to CLTS and reported an incremental cost of $1,205 per household with an improved latrine, with productivity loss included in cost estimations [75,76]. Comparing CLTS with the SOC, Briceño et al. estimated an incremental cost of $251 per household with an improved latrine, though time use estimations were excluded [73].

Trial-based economic evaluation of community-led HIVST compared with the SOC reported an incremental cost per additional person tested HIV positive of $365 from a provider perspective, with 45% probability of cost-effectiveness against a recommended threshold for diagnostics [77]. Results were sensitive to variation in the outcome estimate. In Zimbabwe, unit costs of community-led HIVST were lower compared with early costs of the community-based alternative but higher compared with later implementation costs [58]. In a trial-based comparison of community mobilisation for HIV and IPV prevention against the SOC, Michaels-Igbokwe et al. estimated a provider incremental cost per physical IPV case averted of $582 [78]. Cost measurements included time use associated with community implementation.

Tschampl et al. conducted a trial-based economic evaluation, which had high risk of bias, of community-led dengue control against the SOC from a provider perspective [80]. The analysis reported an incremental cost per disability-adjusted life year averted of $36,809 in Mexico and $36,284 in Nicaragua, with respectively 51% and 0% cost-effectiveness probability against a gross domestic product defined threshold. Low likelihood of cost-effectiveness was attributed to exclusion of societal benefits and costs and high costs of implementation within a randomised trial. Phiri et al. evaluated the societal costs of community-driven larval source management and house improvement for malaria and observed similar costs for both strategies, with costs sensitive to personnel inputs and population coverage [79].

For multi-disease studies, two trial-based economic evaluations assessed participatory women’s groups against an enhanced SOC, with a high risk of bias determined [51,55]. Provider incremental cost per life-year lost averted was $148 in Malawi and provider incremental cost per life-year saved was $1,125 in India. Determinants of costs and cost-effectiveness were not discussed. Makaula et al. assessed total costs of community-directed primary care, which had higher costs than the SOC due to community-level costs including volunteer allowance [52].

### Implementation, mechanisms of impact, and context

Table 2 and Table G in S1 Text summarises results on implementation processes, mechanisms of impact, and contextual factors influencing implementation and outcomes.

**Table 2:** Summary of implementation, mechanisms of impact, and context.

	Implementation		Mechanisms of impact	Context
	Community participation strategies	Communicable disease strategies		
**Facilitators**	Motivation by community actors to gain knowledge and skills [83,84] Nomination of community actors by wider community [52,88]	Variety of activities identified [55,89] Established trust with wider community [86,88] Availability, support, and influence of community actors [83,84,86,90] Proximity [91] Use of participatory and collective approaches [85,86,90,91] Availability, support, and influence of community leaders [52,87] Availability, support, and influence of health care providers [52,87,89] Monitoring and evaluation of outcomes [83,87] Targets and rewards for implementation [56]	Sufficient exposure to CD strategies [49,50,56,59,84,88,91] Sufficient coverage of CD strategies [50,56] Repeated engagement with CD strategies [45,81,87,91] Motivation to address CDs [83,87] Awareness of the benefits of CD strategies [83,88] Diffusion of messages and adoption of CD strategies through social networks [91] Attitudes and norms related to CDs and risk factors [60,66,85,86,90] Social capital [47,61] Community empowerment [66]	Male [50,58,59] Younger age group [50] Social cohesion [72] Female head of household [82] Willingness to change [86] Personal experience related to CDs or risk factors [90]
**Barriers**	Exclusion of marginalised and vulnerable groups [45]	Exclusion of marginalised and vulnerable groups [45] Inadequate engagement of subgroups [87] Labour, time, and costs of CD strategies [45,83,87]	Poor coverage of CD strategies [58]	Attitudes and norms around CDs [83]

CD, communicable disease.

#### Implementation.

Quantitative and qualitative studies reported high levels of involvement by community actors in participatory activities conducted by external actors [50,51,55,56,59]. Community actors leading larval source management and house improvements in Malawi stated in focus group discussions that they were motivated by their desire to gain knowledge and skills in delivering communicable disease strategies and to act as change agents [83,84]. In some studies, community actors were elected by the wider community [52,88]. In Tanzania, community members receiving community-directed treatment distribution for neglected tropical diseases qualitatively reported that such processes were critical to ensuring representation by trusted individuals [88].

Communicable disease strategies varied and were either externally defined and adapted by community actors or identified by community actors through participatory exercises. For example, dengue control strategies in Mexico and Nicaragua included activities, such as household education, that were predetermined by external actors [44]. Other studies involved strategies that were fully decided on by community actors. In Malawi, women’s groups prioritised disease areas and identified a range of maternal and child health activities through participatory meetings. As described in quantitative and qualitative studies, activities included health education, bicycle ambulances, distribution of health commodities, mobile clinics, garden cultivation, and income generation [89].

Support from health care providers and other stakeholders facilitated implementation and created an enabling environment for delivering communicable disease strategies [52,87,89]. In Malawi, women’s groups established linkages with nearby health facilities and collaborated on provision of mobile antenatal and under-5 clinics, as reported in a mixed-methods study [89]. In Mali, externally set targets and rewards were used to support community implementation, with communities receiving certification upon achievement of implementation targets for CLTS activities [56]. Another reported facilitator was trust between community actors and the wider community [86,88]. A qualitative study found that the established relationship between community activists and community members in Uganda was critical for building trust and facilitating uptake of knowledge and practices for HIV and IPV prevention [86]. Based on quantitative and qualitative evidence, availability and support of community activists and their use of participatory activities were also important for facilitating exposure and engagement with community members [85,86,90,91].

Communicable disease strategies that were costly, time consuming, or labour intensive, such as latrine construction or larval source management, were reported to be barriers to implementation [45,83,87]. Further, strategies that did not take into consideration the different needs of population subgroups also acted as barriers. In Malawi, individual interviews found that CLTS with inclusivity training had poor engagement of people with disabilities, meaning marginalised and vulnerable groups were less likely to be considered in sanitation strategies [45]. Another qualitative Malawian study observed that men were less likely to participate in malaria control activities due to time lost from income-generating activities as well as the perception that women were responsible for health care-related activities [87].

#### Mechanisms of impact.

As evidenced through quantitative and qualitative studies, sufficient exposure to and uptake of communicable disease strategies led by community actors were necessary to influence outcomes [49,50,56,59,84,88,91]. Community-led provision of HIVST in Malawi achieved 75% uptake, which likely had a subsequent impact on HIV testing outcomes [50]. In contrast, a similar study in Zimbabwe reported HIVST uptake of 22%, which was lower than uptake in the comparison arm, and thus did not measure an effect on HIV diagnosis and care [58]. Further, repeated exposure to communicable disease strategies was found to be an important mechanism of change [45,81,87,91]. In Uganda, there was strong evidence of a dose-response relationship between increasing exposure to multiple community mobilisation activities and positive changes in interpersonal relationships [91]. Other factors influencing outcomes included motivation to address communicable diseases [83,87] and awareness of the benefits of implemented strategies [83,88]. Based on qualitative data, a Malawian study reported that malaria was considered to be the largest threat to health and acted as a motivation for community members to engage in prevention activities [87].

Mediators of the impact of community-led approaches included attitudes and norms related to communicable diseases and their risk factors [60,66,85,86,90]. In quantitative and qualitative studies in Uganda, community mobilisation was found to contribute to shifting gender norms and power dynamics and enhancing communication and nonviolent conflict resolution between partners, which strengthened interpersonal relationships and reduced IPV risk [60,85,86,90]. There was also some quantitative evidence that changes in community and social-level measures had an impact on downstream outcomes [47,60,61,66]. Abramsky et al. found a statistically significant association between improved gender attitudes and norms at community level and reductions in physical IPV [60]. A Malawian study detected statistically significant associations between measures of community empowerment, such as social cohesion, shared concern, and collective problem solving, and HIV testing following participation in community-led HIVST, though there was no evidence of a mediation effect through community-level variables [66].

#### Context.

At an individual level, studies reported differences in the intervention effect by sex. In trial subgroup analysis, community-led HIVST resulted in greater improvements in coverage of HIV testing and linkage to HIV prevention and care among men compared with women [50,58]. In Indonesia, the intervention effect of CLTS on diarrhoeal prevalence was larger among female heads of households than male household heads [82]. Qualitative evidence from Uganda also showed that community members exposed to community mobilisation activities were more likely to change their behaviours based on personal experience with HIV and IPV [90]. In quantitative analysis, the effect of community-led HIVST on new HIV diagnosis was found to be higher among village groups with high levels of social cohesion in Zimbabwe at a community level, based on moderate evidence [72]. Other factors that impacted the intervention effect included prevailing attitudes and norms around communicable diseases, as evidenced in a qualitative study in Malawi [83]. The perception that larvicide posed health risks contributed to initial lack of trust in malaria control strategies [83].

## Discussion

The main findings of this systematic review were that community-led approaches can improve health behaviours including for diarrhoeal diseases, HIV, malaria, and neglected tropical diseases, based on evidence with moderate risk of bias. Evidence was strongest for diarrhoeal diseases, with multiple cluster-randomised trials reporting consistent improvements in water, hygiene, and sanitation practices. However, evidence for impact on mortality and morbidity, health care access and utilisation, and community and social outcomes was less conclusive, with fewer trials measuring these outcomes and results inconsistent among these studies. We also aimed to summarise evidence on pathways to impact and contexts as well as costs and cost-effectiveness. Process evaluations suggested that impact depended on the intensity of implementation by community actors, and that factors facilitating implementation included motivation to engage and implement communicable disease strategies, trust between community actors and the wider community, and engagement with stakeholders including health care providers. Contextual influences included demographic and social factors, such as social cohesion and attitudes and norms around communicable diseases. Economic studies were few and many omitted societal costs and consequences. Providing clearer operational guidance on how to define and identify strategies for meaningful community participation and capture relevant outcomes, costs, and processes will be critical to support rapid evidence generation in this important and promising area.

Our findings contribute to previous reviews that highlight the potential value of community participation in public health [10,21–27], but underscore difficulties in synthesis due to variability concerning the nature and extent of community participation and the adaptation and implementation of strategies by communities. We found more consistent evidence for positive impact on health behaviours in contrast with other outcome domains, including morbidity and mortality and health care. For example, most trials on diarrhoeal diseases reported consistent improvements in sanitation practices, water infrastructure, and hygiene behaviours, but showed weaker evidence for diarrhoeal disease burden and child development. Positive changes in health behaviours, such as gender roles and norms [59,85], sexual behaviours [59,85], and vector control measures [44], were reported for other disease areas but included fewer studies. Evidence on health care outcomes was difficult to interpret without understanding service-related barriers to provision and use of care. For instance, provision of HIVST was important for addressing supply and demand-side barriers to care and increasing HIV testing coverage [50]. To impact morbidity and mortality, some trials integrated more vertical elements to improve intensity of implementation. Reduction of dengue infection in Mexico and Nicaragua was achieved through a combination of community-driven mobilisation and externally prescribed household education [44]. However, we recognise that drawing conclusions on drivers of effect heterogeneity is challenging with limited studies.

Our synthesis aimed to understand processes underlying the effects of community-led approaches, which are characterised by multicomponent inputs and implementation, nonlinear mechanisms of impact, interactions with contexts, and synergies between outcomes [23]. Included studies reported high levels of community involvement, underscoring the acceptability of community-led strategies for communicable disease control. Desire to gain knowledge and skills and act collectively as change agents motivated implementation by community actors and has previously been described as important for community participation [92]. Other key implementation factors included support from health care providers and other stakeholders as well as trust between community actors and the wider population. In Malawi, women’s groups collaborated with nearby health facilities to provide antenatal care and under-5 services through mobile clinics [89].

Interventions included in our review varied in terms of the scope of community participation and communicable disease strategies. For example, some studies involved external actors predetermining the remit of disease strategies, such as latrine construction [45–49,56]. In other studies, community actors had broader input, such as women’s groups identifying prioritised disease areas and strategies for maternal and child health [51,55]. Choice of approach may vary according to the intended aims of the intervention. For example, biomedical and environmental strategies requiring immediate attention may be more amenable to community-driven implementation of solutions set by external actors. A review of community engagement approaches in high-income countries found that community-based implementation had larger effect sizes than empowerment-based approaches, potentially due to higher intervention intensity [23]. Alternatively, strategies aimed at addressing social and structural determinants of diseases might require more extensive engagement of community actors to impact upstream outcomes.

Reaching sufficient intensity of implementation by community actors was important to meet intended outcomes. For example, high levels of exposure to CLTS events likely facilitated improvements in latrine ownership in Mali [56]. Another hypothesised pathway for improving health outcomes is through modifying social and structural determinants of health [5,6,93]. Some studies reported quantitative and qualitative evidence for indirect effects through community and social outcomes, but data were limited. In Uganda, impact on physical IPV was found to be mediated by gender attitudes and norms at community level [60]. Our review also reported some evidence of population-level impact on community and social outcomes, but with few studies included and inconsistent findings among studies. While impact on upstream determinants of health has been reported in previous reviews [23], our inconclusive findings are not surprising given that community and social outcomes are products of complex systems, difficult to measure, and rarely included in evaluations [26]. Studies are also not often powered to measure these outcomes [26]. Further, impact might be more difficult to achieve if studies are targeting downstream health determinants, with direct intervention on community empowerment likely needed to impact community and social outcomes [94]. For example, changes in collective action and social capital were not observed following community-led environmental management for dengue prevention in Mexico and Nicaragua [61].

Evidence for costs and relative cost-effectiveness against facility and community alternatives was varied, largely due to differences in measurement of costs and outcomes. For example, economic costing of CLTS in Tanzania accounted for in-kind contributions but not volunteer time [73]. Most studies used a provider rather than societal perspective, meaning that direct and indirect costs incurred by communities were largely excluded from cost estimations. Few studies also measured generic or non-health consequences as well as long-term costs and outcomes, potentially underestimating benefits from community participation. When broader costs and benefits were modelled, interventions were found to generate net benefits [74]. These gaps underscore the need for standardised guidance for measuring costs and benefits in this methodologically challenging area [95]. Systematic capture of community costs is especially important given the potential for the benefits of community engagement to be offset by the time and financial burden of involvement [21]. Further, there is a risk that decentralisation of resource use will be exploited as an alternative to the substantial investment required for community-based strategies [22]. Therefore, it is important that funding for community-led responses appropriately account for community costs with systems in place to support financial sustainability, such as integrating social contracting into national and global health financing structures.

Our review underscores the need for evaluations of community-led interventions to adopt methodological approaches that effectively measure the nature and extent of community participation and its influence on health and non-health outcomes. The framework used in this review to classify community participation was adapted from an existing instrument that has been applied in another systematic review [14,18,21,23]. This framework can act as a practical tool for defining key concepts and practices underlying community participation and supporting more complete and consistent reporting. To understand process mechanisms, metrics should also capture levels of decision making, time spent on activities, degree of community ownership, representativeness of decision makers, and community satisfaction with the process of participation and achievement of goals [20]. Lastly, full costs incurred by community actors should be documented to understand the extent of resources contributed.

Reviews of community participation have previously highlighted the challenges of evidence synthesis. Interventions involving participatory approaches consist of multiple independent and interdependent components that seek to influence a complex system [96,97]. Community participation is fluid and can evolve over time, and implementation will differ based on the needs, resources, and conditions of communities [95,98]. Participation by communities can generate both health and non-health effects that can occur at individual and community levels, immediate and extended time horizons and through direct and indirect exposure that differ by context [95–97]. To address heterogeneity concerning community participation, we restricted our eligibility criteria to community-led approaches. However, there was still substantial variation in terms of the degree of community ownership. Ascertaining study eligibility required subjective interpretations and judgements due to differences in terminology used by authors. Not all studies reported implementation procedures in sufficient detail to understand how community actors were engaged and how strategies were developed by community actors. Mechanisms of impact and contextual factors that might support or hinder impact were also not consistently described and should be prioritised in reporting.

Our review had additional limitations, including the broad scope of disease areas and strategies for communicable diseases. As a result, meta-analysis was not appropriate due to heterogeneity in study characteristics [38]. Certainty of evidence and risk of publication bias were also not assessed. We attempted to address study variability by grouping interventions and outcomes into domains to assess evidence across disease areas. To improve methodological quality of effectiveness studies, we restricted our review to cluster-randomised trials, which have well-known limitations in terms of their application to complex interventions [97]. As a result, our conclusions are based on interventions done in controlled settings, with external actors potentially having a greater role than in pragmatic contexts. Comparators within trials varied, meaning the intervention effect may have captured other differences between arms besides community participation. For example, trials on HIVST used different facility and community comparators [50,58]. Our search was based on broad terms for ‘community’, potentially excluding studies that referred to specific groups. Most studies in our review include communities defined by spatial rather than social characteristics. Lastly, one of the included studies was conducted by authors of the current review, though we aimed to reduce bias with an independent second reviewer.

This systematic literature review of community-led communicable disease control strategies showed stronger evidence for impact on health behaviours, but less conclusive data for morbidity and mortality, health care access and utilisation, and community and social outcomes. Impact depended on the intensity of community implementation, with factors facilitating implementation including motivation by community actors, trust between community actors and the wider population, and engagement with the health system. Our synthesis highlights the need for consensus on and use of an operational framework for community-led approaches to define key concepts and practices, support more complete and consistent reporting, including on costs and processes, and enable lessons to be learnt across health and development. Further, this review supports community-led approaches as a potentially effective strategy to impact health behaviours and contribute to SDGs. Given the current global context of disruptive shocks to health, social, and economic systems, greater focus on generating evidence and establishing systems to support design and scale-up of community-led health responses should be considered a global priority.

## Supporting information

S1 PRISMA Checklist Checklist of information to include when reporting a systematic review. (DOCX)

S1 Text Supporting information. Supporting text, tables, and figures.
(DOCX)

S1 Table List of records reviewed at full text. List of records reviewed at full text with reasons for inclusion and exclusion.
(DOCX)

## References

[1] WHO. Health in 2015: from MDGs, Millennium Development Goals to SDGs, Sustainable Development Goals. Geneva: World Health Organization, 2015.

[2] Vos T, Lim SS, Abbafati C, Abbas KM, Abbasi M, Abbasifard M, et al.; GBD 2019 Diseases and Injuries Collaborators Global burden of 369 diseases and injuries in 204 countries and territories, 1990–2019: a systematic analysis for the Global Burden of Disease Study 2019. The Lancet. 2020;396(10258):1204–22. doi: 10.1016/s0140-6736(20)30925-9 PMC7567026 33069326

[3] WHO. Primary health care: Report of the International Conference on Primary Health Care Alma Ata, USSR, 6–12 September 1978. Geneva: World Health Organization (WHO), 1978.

[4] Lawn JE, Rohde J, Rifkin S, Were M, Paul VK, Chopra M. Alma-Ata 30 years on: revolutionary, relevant, and time to revitalise. The Lancet. 2008;372(9642):917–27. doi: 10.1016/s0140-6736(08)61402-6 18790315

[5] Rifkin SB. Paradigms lost: Toward a new understanding of community participation in health programmes. Acta Tropica. 1996;61(2):79–92. doi: 10.1016/0001-706x(95)00105-n 8740887

[6] Zakus JD, Lysack CL. Revisiting community participation. Health Policy Plan. 1998;13(1):1-12. doi: 10.1093/heapol/13.1.1 . 10178181

[7] UNDP. Understanding and acting on critical enablers and development synergies for strategic investments. New York: United Nations Development Programme (UNDP), 2012.

[8] UNAIDS. Fast-track commitments to end AIDS by 2030. Geneva: Joint United Nations Programme on HIV/AIDS (UNAIDS), 2016.

[9] McGowan CR, Takahashi E, Romig L, Bertram K, Kadir A, Cummings R, et al. Community-based surveillance of infectious diseases: a systematic review of drivers of success. BMJ Glob Health. 2022;7(8):e009934. doi: 10.1136/bmjgh-2022-009934 PMC9396156 35985697

[10] Rifkin SB. Examining the links between community participation and health outcomes: a review of the literature. Health Policy Plan. 2014;29(Suppl 2):ii98-106. doi: 10.1093/heapol/czu076 ; PMCID: PMC4202913 25274645 PMC4202913

[11] Rifkin SB. Alma Ata after 40 years: primary health care and health for All. From consensus to complexity. BMJ Glob Health. 2018;3(Suppl 3):e001188. doi: 10.1136/bmjgh-2018-001188 ; PMCID: PMC6307566 30622747 PMC6307566

[12] Woelk GB. Cultural and structural influences in the creation of and participation in community health programmes. Social Science & Medicine. 1992;35(4):419–24. doi: 10.1016/0277-9536(92)90334-m 1519094

[13] Arnstein SR. A Ladder Of Citizen Participation. Journal of the American Institute of Planners. 1969;35(4):216–24. doi: 10.1080/01944366908977225

[14] Draper A, Hewitt G, Rifkin S. Chasing the dragon: developing indicators for the assessment of community participation in health programmes. Soc Sci Med. 2010;71(6):1102–9. doi: 10.1016/j.socscimed.2010.05.016 20621405

[15] Labonte R. Health Promotion and Empowerment: Practice Frameworks. Toronto: Centre for Health Promotion, University of Toronto, 1993.

[16] Laverack G, Labonte R. A planning framework for community empowerment goals within health promotion. Health Policy and Planning. 2000;15(3):255–62. doi: 10.1093/heapol/15.3.255 11012399

[17] McLeroy K, Norton B, Kegler M, Burdine J, Sumaya C. Community-based interventions. Am J Public Health. 2003;93(4):529–33. doi: 10.2105/ajph.93.4.529 12660190 PMC1447783

[18] Rifkin S, Pridmore P. Partners in Planning: Information, Participation and Empowerment. 1st ed. London: Macmillan Education Ltd;. 2001.

[19] Rothman J, Erlich J, Tropman J. Strategies of Community Intervention. 1st ed. Itasca: F.E. Peacock Publishers; 2001.

[20] Butterfoss F. Process evaluation for community participation. Annual Review of Public Health. 2006;27(1):323–40. doi: 10.1146/annurev.publhealth.27.021405.102207 16533120

[21] Attree P, French B, Milton B, Povall S, Whitehead M, Popay J. The experience of community engagement for individuals: a rapid review of evidence. Health & Social Care in the Community. 2011;19(3):250–60. doi: 10.1111/j.1365-2524.2010.00976.x 21138495

[22] Atkinson JA, Vallely A, Fitzgerald L, Whittaker M, Tanner M. The architecture and effect of participation: a systematic review of community participation for communicable disease control and elimination. Implications for malaria elimination. Malar J. 2011;10:225. doi: 10.1186/1475-2875-10-225 ; PMCID: PMC3171376 21816085 PMC3171376

[23] O’Mara-Eves A, Brunton G, Oliver S, Kavanagh J, Jamal F, Thomas J. The effectiveness of community engagement in public health interventions for disadvantaged groups: a meta-analysis. BMC Public Health. 2015;15(1):129. doi: 10.1186/s12889-015-1352-y ; PMCID: PMC4374501 25885588 PMC4374501

[24] Questa K, Das M, King R, Everitt M, Rassi C, Cartwright C, et al. Community engagement interventions for communicable disease control in low- and lower-middle-income countries: evidence from a review of systematic reviews. International Journal for Equity in Health. 2020;19(1):51. doi: 10.1186/s12939-020-01169-5 32252778 PMC7137248

[25] Ayala G, Sprague L, van der Merwe LL, Thomas RM, Chang J, Arreola S, et al. Peer- and community-led responses to HIV: a scoping review. PLOS One. 2021;16(12):e0260555. doi: 10.1371/journal.pone.0260555 ; PMCID: PMC8635382 34852001 PMC8635382

[26] Cornish F, Priego-Hernandez J, Campbell C, Mburu G, McLean S. The impact of community mobilisation on HIV prevention in middle and low income countries: a systematic review and critique. AIDS Behav. 2014;18(11):2110-34. doi: 10.1007/s10461-014-0748-5 ; PMCID: PMC4196137 24659360 PMC4196137

[27] Kerrigan D, Kennedy C, Morgan-Thomas R, Reza-Paul S, Mwangi P, Win K, et al. A community empowerment approach to the HIV response among sex workers: effectiveness, challenges, and considerations for implementation and scale-up. Lancet. 2015;385(9963):172–85. doi: 10.1016/S0140-6736(14)60973-9 25059938 PMC7394498

[28] Singer M, Bulled N, Ostrach B, Mendenhall E. Syndemics and the biosocial conception of health. The Lancet. 2017;389(10072):941–50. doi: 10.1016/s0140-6736(17)30003-x 28271845

[29] Higgins J, Thomas J, Chandler J, Cumpston M, Li T, Page M, et al. Cochrane handbook for systematic reviews of interventions. 2022. [updated February 2022]. Available from: www.training.cochrane.org/handbook 10.1002/14651858.ED000142 PMC10284251 31643080

[30] Page MJ, McKenzie JE, Bossuyt PM, Boutron I, Hoffmann TC, Mulrow CD, et al. The PRISMA 2020 statement: an updated guideline for reporting systematic reviews. BMJ. 2021;372:n71. doi: 10.1136/bmj.n71 PMC8005924 33782057

[31] UNAIDS. Establishing community-led monitoring of HIV services. Geneva: Joint United Nations Programme on HIV/AIDS (UNAIDS), 2021.

[32] Minkler M, Wallerstein N. Improving Health Through Community Organization and Community Building. In: Glanz K, Rimer BK, Lewis FM, editors. Health Behavior and Health Education: Theory, Research, and Practice. 5th ed. San Francisco: Josey-Bass; 2015.

[33] WHO. Community-directed interventions for major health problems in Africa. Geneva: World Health Organization (WHO), 2008.

[34] WHO. WHO recommendation on community mobilisation through facilitated participatory learning and action cycles with women’s groups for maternal and newborn health. Geneva: World Health Organization (WHO), 2014. 25165805

[35] WHO. Community engagement: a health promotion guide for universal health coverage in the hands of the people. Geneva: World Health Organization (WHO), 2020.

[36] Drummond M, Jefferson T. Guidelines for authors and peer reviewers of economic submissions to the BMJ. BMJ. 1996;313(7052):275–83. doi: 10.1136/bmj.313.7052.275 8704542 PMC2351717

[37] Higgins JP, Altman DG, Gotzsche PC, Juni P, Moher D, Oxman AD, et al. The Cochrane Collaboration’s tool for assessing risk of bias in randomised trials. BMJ. 2011;343:d5928. doi: 10.1136/bmj.d5928 ; PMCID: PMC3196245 22008217 PMC3196245

[38] Campbell M, McKenzie JE, Sowden A, Katikireddi SV, Brennan SE, Ellis S, et al. Synthesis without meta-analysis (SWiM) in systematic reviews: reporting guideline. BMJ. 2020;368:l6890. doi: 10.1136/bmj.l6890 31948937 PMC7190266

[39] Ogilvie D, Fayter D, Petticrew M, Sowden A, Thomas S, Whitehead M, et al. The harvest plot: a method for synthesising evidence about the differential effects of interventions. BMC Med Res Methodol. 2008;8(1):8. doi: 10.1186/1471-2288-8-8 ; PMCID: PMC2270283 18298827 PMC2270283

[40] EPPI-Centre. CCEMG - EPPI-Centre Cost Converter 2019 [updated April 2019]. Available from: https://eppi.ioe.ac.uk/costconversion/

[41] Moore GF, Audrey S, Barker M, Bond L, Bonell C, Hardeman W, et al. Process evaluation of complex interventions: Medical Research Council guidance. BMJ. 2015;350:h1258. doi: 10.1136/bmj.h1258 ; PMCID: PMC4366184 25791983 PMC4366184

[42] Hong Q, Pluye P, Bujold M, Wassef M. Convergent and sequential synthesis designs: implications for conducting and reporting systematic reviews of qualitative and quantitative evidence. Systematic Reviews. 2017;6(1):61. doi: 10.1186/s13643-017-0454-2 28335799 PMC5364694

[43] Abramsky T, Devries K, Michau L, Nakuti J, Musuya T, Kyegombe N. The impact of SASA!, a community mobilisation intervention, on women’s experiences of intimate partner violence: secondary findings from a cluster randomised trial in Kampala, Uganda. J Epidemiol Community Health. 2016;70(8):818–25. doi: 10.1136/jech-2015-206665 26873948 PMC4975800

[44] Andersson N, Nava-Aguilera E, Arosteguí J, Morales-Perez A, Suazo-Laguna H, Legorreta-Soberanis J, et al. Evidence based community mobilization for dengue prevention in Nicaragua and Mexico (Camino Verde,the Green Way): cluster randomized controlled trial. BMJ. 2015;351:h3267. doi: 10.1136/bmj.h3267 26156323 PMC4495677

[45] Biran A, Danquah L, Chunga J, Schmidt WP, Holm R, Itimu-Phiri A, et al. A cluster-randomised trial to evaluate the impact of an inclusive, community-led total sanitation intervention on sanitation access for people with disabilities in Malawi. Am J Trop Med Hyg. 2018;98(4):984-94. doi: 10.4269/ajtmh.17-0435 ; PMCID: PMC5928815 29405106 PMC5928815

[46] Briceño B, Coville A, Gertler P, Martinez S. Are there synergies from combining hygiene and sanitation promotion campaigns: evidence from a large-scale cluster-randomised trial in rural Tanzania. PLOS One. 2017;12(11):e0186228. doi: 10.1371/journal.pone.0186228 29091726 PMC5665426

[47] Cameron L, Olivia S, Shah M. Scaling up sanitation: Evidence from an RCT in Indonesia. Journal of Development Economics. 2019;138(1):1–16. doi: 10.1016/j.jdeveco.2018.12.001 31057208 PMC6472610

[48] Cha S, Jung S, Bizuneh D, Abera T, Doh Y, Seong J, et al. Effect of a community-led total sanitation intervention on the incidence and prevalence of diarrhoea in children in rural Ethiopia: a cluster-randomised controlled trial. Am J Trop Med Hyg. 2021;105(2):532–43. doi: 10.4269/ajtmh.20-0014 34125700 PMC8437198

[49] Crocker J, Abodoo E, Asamani D, Domapielle W, Gyapong B, Bartram J. Impact evaluation of training natural leaders during a community-led total sanitation intervention: a cluster-randomised field trial in Ghana. Environ Sci Technol. 2016;50(16):8867-75. doi: 10.1021/acs.est.6b01557 ; PMCID: PMC4989246 27428399 PMC4989246

[50] Indravudh P, Fielding K, Kumwenda M, Nzawa R, Chilongosi R, Desmond N, et al. Effect of community-led delivery of HIV self-testing on HIV testing and antiretroviral therapy initiation in Malawi: a cluster-randomised trial. PLOS Medicine. 2021;18(5):e1003608. doi: 10.1371/journal.pmed.1003608 33974621 PMC8112698

[51] Lewycka S, Mwansambo C, Rosato M, Kazembe P, Phiri T, Mganga A, et al. Effect of women’s groups and volunteer peer counselling on rates of mortality, morbidity, and health behaviours in mothers and children in rural Malawi (MaiMwana): a factorial, cluster-randomised controlled trial. Lancet. 2013;381(9879):1721-35. doi: 10.1016/S0140-6736(12)61959-X ; PMCID: PMC3796349 23683639 PMC3796349

[52] Makaula P, Funsanani M, Mamba KC, Musaya J, Bloch P. Strengthening primary health care at district-level in Malawi - determining the coverage, costs, and benefits of community-directed interventions. BMC Health Serv Res. 2019;19(1):509. doi: 10.1186/s12913-019-4341-5 ; PMCID: PMC6647329 31331346 PMC6647329

[53] Massa K, Magnussen P, Sheshe A, Ntakamulenga R, Ndawi B, Olsen A. The effect of the community-directed treatment approach versus the school-based treatment approach on the prevalence and intensity of schistosomiasis and soil-transmitted helminthiasis among schoolchildren in Tanzania. Trans R Soc Trop Med Hyg. 2009;103(1):31–7. doi: 10.1016/j.trstmh.2008.07.009 18771789

[54] McCann RS, Kabaghe AN, Moraga P, Gowelo S, Mburu MM, Tizifa T, et al. The effect of community-driven larval source management and house improvement on malaria transmission when added to the standard malaria control strategies in Malawi: a cluster-randomised controlled trial. Malar J. 2021;20(1):232. doi: 10.1186/s12936-021-03769-0 ; PMCID: PMC8140568 34022912 PMC8140568

[55] Nair N, Tripathy P, Sachdev H, Pradhan H, Bhattacharyya S, Gope R, et al. Effect of participatory women’s groups and counselling through home visits on children’s linear growth in rural eastern India (CARING trial): a cluster-randomised controlled trial. Lancet Glob Health. 2017;5(10):e1004–16. doi: 10.1016/S2214-109X(17)30339-X 28911749 PMC5640793

[56] Pickering AJ, Djebbari H, Lopez C, Coulibaly M, Alzua ML. Effect of a community-led sanitation intervention on child diarrhoea and child growth in rural Mali: a cluster-randomised controlled trial. The Lancet Global Health. 2015;3(11):e701–11. doi: 10.1016/s2214-109x(15)00144-8 26475017

[57] Quattrochi JP, Coville A, Mvukiyehe E, Dohou CJ, Esu F, Cohen B, et al. Effects of a community-driven water, sanitation and hygiene intervention on water and sanitation infrastructure, access, behaviour, and governance: a cluster-randomised controlled trial in rural Democratic Republic of Congo. BMJ Glob Health. 2021;6(5):e005030. doi: 10.1136/bmjgh-2021-005030 PMC8130731 34001519

[58] Sibanda E, Mangenah C, Neuman M, Tumushime M, Watadzaushe C, Mutseta M, et al. Comparison of community-led distribution of HIV self-tests kits with distribution by paid distributors: a cluster randomised trial in rural Zimbabwean communities. BMJ Global Health. 2021;6(Suppl 4):. doi: 10.1136/bmjgh-2021-005000 34275872 PMC8287604

[59] Abramsky T, Devries K, Kiss L, Nakuti J, Kyegombe N, Starmann E, et al. Findings from the SASA! Study: a cluster randomised controlled trial to assess the impact of a community mobilisation intervention to prevent violence against women and reduce HIV risk in Kampala, Uganda. BMC Med. 2014;12:122. doi: 10.1186/s12916-014-0122-5 ; PMCID: PMC4243194 25248996 PMC4243194

[60] Abramsky T, Devries KM, Michau L, Nakuti J, Musuya T, Kiss L, et al. Ecological pathways to prevention: How does the SASA! community mobilisation model work to prevent physical intimate partner violence against women?. BMC Public Health. 2016;16(1):. doi: 10.1186/s12889-016-3018-9 PMC4833941 27084116

[61] Alvarado-Castro V, Paredes-Solis S, Nava-Aguilera E, Morales-Perez A, Flores-Moreno M, Legorreta-Soberanis J, et al. Social capital is associated with lower mosquito vector indices: secondary analysis from a cluster randomised controlled trial of community mobilisation for dengue prevention in Mexico. Popul Health Metr. 2019;17(1):18. doi: 10.1186/s12963-019-0199-3 ; PMCID: PMC6902442 31823786 PMC6902442

[62] Carcamo A, Arostegui J, Coloma J, Harris E, Ledogar R, Andersson N. Informed community mobilisation for dengue prevention in households with and without a regular water supply: secondary analysis from the Camino Verde trial in Nicaragua. BMC Public Health. 2017;17(Suppl 1):395. doi: 10.1186/s12889-017-4295-7 28699544 PMC5506562

[63] Crocker J, Saywell D, Bartram J. Sustainability of community-led total sanitation outcomes: Evidence from Ethiopia and Ghana. Int J Hyg Environ Health. 2017;220(3):551-7. doi: 10.1016/j.ijheh.2017.02.011 ; PMCID: PMC5475437 28522255 PMC5475437

[64] Croke K, Coville A, Mvukiyehe E, Dohou CJ, Zibika JP, Stanus Ghib L, et al. Effects of a community-driven water, sanitation, and hygiene programme on COVID-19 symptoms, vaccine acceptance and non-COVID illnesses: a cluster-randomised controlled trial in rural Democratic Republic of Congo. Trop Med Int Health. 2022;27(9):795-802. doi: 10.1111/tmi.13799 ; PMCID: PMC9349788 35832019 PMC9349788

[65] Gowelo S, Meijer P, Tizifa T, Malenga T, Mburu M, Kabaghe A, et al. Community participation in habitat management and larviciding for the control of malaria vectors in southern Malawi. American Journal of Tropical Medicine and Hygiene. 2023;108(1):51–60. doi: 10.4269/ajtmh.21-1127 36410320 PMC9833073

[66] Indravudh PP, Terris-Prestholt F, Neuman M, Kumwenda MK, Chilongosi R, Johnson CC, et al. Understanding mechanisms of impact from community-led delivery of HIV self-testing: mediation analysis of a cluster-randomised trial in Malawi. PLOS Glob Public Health. 2022;2(10):e0001129. doi: 10.1371/journal.pgph.0001129 ; PMCID: PMC10021599 36962622 PMC10021599

[67] Jimenez-Alejo A, Morales-Perez A, Nava-Aguilera E, Flores-Moreno M, Apreza-Aguilar S, Carranza-Alcaraz W. Pupal productivity in rainy and dry seasons: findings from the impact survey of a randomised controlled trial of dengue prevention in Guerrero, Mexico. BMC Public Health. 2017;17(Suppl 1):428. doi: 10.1186/s12889-017-4294-8 28699555 PMC5506597

[68] Legorreta-Soberanis J, Paredes-Solís S, Morales-Pérez A, Nava-Aguilera E, de los Santos FRS, Sánchez-Gervacio BM, et al. Coverage and beliefs about temephos application for control of dengue vectors and impact of a community-based prevention intervention: secondary analysis from the Camino Verde trial in Mexico. BMC Public Health. 2017;17(S1):426. doi: 10.1186/s12889-017-4297-5 28699554 PMC5506576

[69] Legorreta-Soberanis J, Paredes-Solis S, Morales-Perez A, Nava-Aguilera E, Serrano-de Los Santos F, Dimas-Garcia D, et al. Household costs of dengue illness: secondary outcomes from a randomised controlled trial of dengue prevention in Guerrero state, Mexico. BMC Public Health. 2017;17(Suppl 1):411. doi: 10.1186/s12889-017-4304-x 28699565 PMC5506602

[70] Legorreta-Soberanis J, Paredes-Solís S, Morales-Pérez A, Nava-Aguilera E, Serrano-de los Santos FR, Sánchez-Gervacio BM, et al. Household costs for personal protection against mosquitoes: secondary outcomes from a randomised controlled trial of dengue prevention in Guerrero state, Mexico. BMC Public Health. 2017;17(S1):399. doi: 10.1186/s12889-017-4303-y 28699550 PMC5506592

[71] Massa K, Olsen A, Sheshe A, Ntakamulenga R, Ndawi B, Magnussen P. Can coverage of schistosomiasis and soil transmitted helminthiasis control programmes targeting school-aged children be improved? New approaches. Parasitology. 2009;136(13):1781–8. doi: 10.1017/s0031182008000474 19178756

[72] Thomas KA, Sibanda EL, Johnson C, Watadzaushe C, Ncube G, Hatzold K, et al. Do community measures impact the effectiveness of a community led HIV testing intervention. Secondary analysis of an HIV self-testing intervention in rural communities in Zimbabwe. BMC Infect Dis. 2023;22(Suppl 1):974. doi: 10.1186/s12879-023-08695-x ; PMCID: PMC10617038 37907871 PMC10617038

[73] Briceño B, Chase C. Cost-efficiency of rural sanitation promotion: activity-based costing and experimental evidence from Tanzania. Journal of Development Effectiveness. 2015 1–12. doi: 10.1080/19439342.2015.1105848

[74] Cha S, Jung S, Bizuneh D, Abera T, Doh Y, Seong J, et al. Benefits and costs of a community-led total sanitation intervention in rural Ethiopia: a trial-based ex post economic evaluation. International Journal of Environmental Research and Public Health. 2020;17(14):. doi: 10.3390/ijerph17145068 32674392 PMC7399893

[75] Crocker J, Saywell D, Shields K, Kolsky P, Bartram J. The true costs of participatory sanitation: Evidence from community-led total sanitation studies in Ghana and Ethiopia. Science of the Total Environment. 2017;601–602:1075–83. doi: 10.1016/j.scitotenv.2017.05.279 28599364 PMC5536257

[76] Crocker J, Fuente D, Bartram J. Cost-effectiveness of community-led total sanitation in Ethiopia and Ghana. International Journal of Hygiene and Environmental Health. 2021;232:113682. doi: 10.1016/j.ijheh.2020.113682 33360500 PMC7873587

[77] Indravudh PP, Fielding K, Sande LA, Maheswaran H, Mphande S, Kumwenda MK, et al. Pragmatic economic evaluation of community-led delivery of HIV self-testing in Malawi. BMJ Glob Health. 2021;6(Suppl 4):e004593. doi: 10.1136/bmjgh-2020-004593 PMC8287609 34275869

[78] Michaels-Igbokwe C, Abramsky T, Devries K, Michau L, Musuya T, Watts C. Cost and cost-effectiveness analysis of a community mobilisation intervention to reduce intimate partner violence in Kampala, Uganda. BMC Public Health. 2016;16(1):. doi: 10.1186/s12889-016-2883-6 PMC4770522 26924488

[79] Phiri MD, McCann RS, Kabaghe AN, van den Berg H, Malenga T, Gowelo S, et al. Cost of community-led larval source management and house improvement for malaria control: a cost analysis within a cluster-randomised trial in a rural district in Malawi. Malar J. 2021;20(1):268. doi: 10.1186/s12936-021-03800-4 ; PMCID: PMC8200285 34120608 PMC8200285

[80] Tschampl CA, Undurraga EA, Ledogar RJ, Coloma J, Legorreta-Soberanis J, Paredes-Solis S, et al. Cost-effectiveness of community mobilisation (Camino Verde) for dengue prevention in Nicaragua and Mexico: a cluster randomised controlled trial. Int J Infect Dis. 2020;94:59-67. doi: 10.1016/j.ijid.2020.03.026 PMID: 32179138. 32179138

[81] Abramsky T, Musuya T, Namy S, Watts C, Michau L. Changing the norms that drive intimate partner violence: findings from a cluster randomised trial on what predisposes bystanders to take action in Kampala, Uganda. BMJ Glob Health. 2018;3(6):e001109. doi: 10.1136/bmjgh-2018-001109 ; PMCID: PMC6304103 30613427 PMC6304103

[82] Christian Borja-Vega CB-V. The effects of the Total Sanitation and Sanitation Marketing programme on gender and ethnic groups in Indonesia. Waterlines. 2014;33(1):55–70. doi: 10.3362/1756-3488.2014.007

[83] Gowelo S, McCann R, Koenraadt C, Takken W, van den Berg H, Manda-Taylor L. Community factors affecting participation in larval source management for malaria control in Chikwawa district, southern Malawi. Malaria Journal. 2020;19(1):195. doi: 10.1186/s12936-020-03268-8 32487233 PMC7265157

[84] Kaunda-Khangamwa BN, van den Berg H, McCann RS, Kabaghe A, Takken W, Phiri K, et al. The role of health animators in malaria control: a qualitative study of the health animator approach within the Majete malaria project in Chikwawa District, Malawi. BMC Health Serv Res. 2019;19(1):478. doi: 10.1186/s12913-019-4320-x ; PMCID: PMC6624973 31299974 PMC6624973

[85] Kyegombe N, Abramsky T, Devries K, Starmann E, Michau L, Nakuti J. The impact of SASA!, a community mobilisation intervention, on reported HIV-related risk behaviours and relationship dynamics in Kampala, Uganda. Journal of the International AIDS Society. 2014;17(1):19232. doi: 10.7448/IAS.17.1.19232 ; PMCID: PMC4223282 25377588 PMC4223282

[86] Kyegombe N, Starmann E, Devries KM, Michau L, Nakuti J, Musuya T, et al. ‘SASA! is the medicine that treats violence’. Qualitative findings on how a community mobilisation intervention to prevent violence against women created change in Kampala, Uganda. Global Health Action. 2014;7(1):25082. doi: 10.3402/gha.v7.25082 25226421 PMC4165071

[87] Malenga T, Kabaghe A, Manda-Taylor L, Kadama A, McCann R, Phiri K, et al. Malaria control in rural Malawi: implementing peer health education for behaviour change. Global Health. 2017;13(1):84. doi: 10.1186/s12992-017-0309-6 29157284 PMC5694909

[88] Massa K, Magnussen P, Sheshe A, Ntakamulenga R, Ndawi B, Olsen A. Community perceptions on the community-directed treatment and school-based approaches for the control of schistosomiasis and soil-transmitted helminthiasis among school-age children in Lushoto District, Tanzania. J Biosoc Sci. 2009;41(1):89-105. doi: 10.1017/s0021932008002964 18647439

[89] Rosato M, Malamba F, Kunyenge B, Phiri T, Mwansambo C, Kazembe P, et al. Strategies developed and implemented by women’s groups to improve mother and infant health and reduce mortality in rural Malawi. International Health. 2012;4(3):176–84. doi: 10.1016/j.inhe.2012.03.007 24029397

[90] Starmann E, Collumbien M, Kyegombe N, Devries K, Michau L, Musuya T, et al. Exploring couples’ processes of change in the context of SASA!, a violence against women and HIV prevention intervention in Uganda. Prevention Science. 2017;18(2):233–44. doi: 10.1007/s11121-016-0716-6 27682273 PMC5243896

[91] Starmann E, Heise L, Kyegombe N, Devries K, Abramsky T, Michau L, et al. Examining diffusion to understand the how of SASA!, a violence against women and HIV prevention intervention in Uganda. BMC Public Health. 2018;18(1):616. doi: 10.1186/s12889-018-5508-4 ; PMCID: PMC5948738 29751754 PMC5948738

[92] De Weger E, Van Vooren N, Luijkx K, Baan C, Drewes H. Achieving successful community engagement: a rapid realist review. BMC Health Services Research. 2018;18(1):285. doi: 10.1186/s12913-018-3090-1 29653537 PMC5899371

[93] Shiell A, Hawe P. Health promotion community development and the tyranny of individualism. Health Econ. 1996;5(3):241–7. doi: 10.1002/(sici)1099-1050(199605)5:3<241::aid-hec197>3.0.co;2-g 8817298

[94] Lippman SA, Maman S, MacPhail C, Twine R, Peacock D, Kahn K, et al. Conceptualising community mobilisation for HIV prevention: implications for HIV prevention programming in the African context. PLOS One. 2013;8(10):e78208. doi: 10.1371/journal.pone.0078208 ; PMCID: PMC3795620 24147121 PMC3795620

[95] Pronyk P, Shchaefer J, Somers M, Heise L. Evaluating structural interventions in public health: challenges, options and global best practice. Structural Approaches in Public Health. 1st ed. New York: Routledge; 2013.

[96] Shiell A, Hawe P, Gold L. Complex interventions or complex systems? Implications for health economic evaluation. BMJ. 2008;336(7656):1281–3. doi: 10.1136/bmj.39569.510521.AD 18535071 PMC2413333

[97] Campbell M, Fitzpatrick R, Haines A, Kinmonth A, Sandercock P, Spiegelhalter D, et al. Framework for design and evaluation of complex interventions to improve health. BMJ. 2000;321(7262):694–6. doi: 10.1136/bmj.321.7262.694 10987780 PMC1118564

[98] Bonell C, Hargreaves J, Strange V, Pronyk P, Porter J. Should structural interventions be evaluated using RCTs? The case of HIV prevention. Social Science & Medicine. 2006;63(5):1135–42. doi: 10.1016/j.socscimed.2006.03.026 16697512

