# Supplementary material for: Community-led strategies for communicable disease prevention and management in low- and middle- income countries: A mixed-methods systematic review of health, social, and economic impact
Source: PLOS Glob Public Health. 2025 Apr 2;5(4):e0004304. doi: 10.1371/journal.pgph.0004304 (PMC11964228; doi:10.1371/journal.pgph.0004304)
Supplement: S1 Text — Supporting text, tables, and figures. (DOCX) [file pgph.0004304.s002.docx]

**Supporting information**

Table of Contents

[Text A. Frameworks for community participation 2](#_Toc133495269)

[Text B. Eligibility criteria and search strategy for database searches 3](#_Toc133495270)

[Text C. Data extraction form 7](#_Toc133495271)

[Text D. Adapted framework for community participation 10](#_Toc133495272)

[Table A. Results for community participation scores 11](#_Toc133495273)

[Table B. Risk of bias assessment for cluster-randomised trials 15](#_Toc133495274)

[Table C. Risk of bias assessment for economic evaluations 16](#_Toc133495275)

[Table D. Results from cluster-randomised trials 17](#_Toc133495276)

[Table E. Results from costing studies 28](#_Toc133495277)

[Table F. Results from economic evaluations 31](#_Toc133495278)

[Table G. Results from process evaluations 33](#_Toc133495279)

[Figure A. Radar graph of community participation scores 39](#_Toc133495280)

## Text A. Frameworks for community participation

| **Author** | **Description** |
| --- | --- |
| Arnstein [1] | Ranks citizen participation in public planning based on the level of power held by citizens. **‘Non-participation’** excludes citizens from planning and includes sub-categories ‘manipulation’ and ‘therapy’. In **‘tokenism’**, the have-nots “hear and have a voice” but lack the power to ensure meaningful adoption by the haves. Sub-categories including ‘informing’, ‘consultation’, and ‘placation’. In **‘citizen power’**, citizens have increasing decision making power in planning, from ‘partnership’ to ‘delegated power’ to ‘citizen control’. |
| Labonte [2] | Differentiates between community-based and community development approaches based on “who sets the agenda and who names the issue or problem”. **‘Community-based’** involves external actors defining community problems and solutions, with assistance from the community. **‘Community development’** involves the process of supporting the community to identify priority concerns and issues and plan and implement strategies in response, aiming to shift power relations between external actors and the community towards greater equity. |
| Laverack and Labonte [3] | Describes frameworks for community participation in health promotion. A **‘top-down’** approach involves external actors following a predetermined cycle of programme design, implementation, and evaluation. In a **‘bottom-up’** approach, programme cycles are negotiated, with external actors supporting the community “in the identification of issues which are important and relevant to their lives and enable them to develop strategies to resolve these issues”. |
| McLeroy et al. [4] | Conceptualises the community as either a ‘setting’, ‘target’, ‘resource’, or ‘agent’ of community-based health interventions. As a **‘setting’**, the community is the geographic location in which interventions are implemented by external actors. The community as a **‘target’** refers to externally led interventions that aim to change behaviours at the community-level rather than the individual-level. Interventions with the community as a **‘resource’** aim to channel resources from the community towards priority health strategies, with external actors working through community institutions and resources. As an **‘agent’**, the community is a “unit of solution” that functions to meet the needs of community members. The role of the external actor is to strengthen the capacity of the community to respond to these needs. |
| Rothman et al. [5] | Specifies three models of community organisation. **‘Planning and policy’** is task oriented, whereby empirical data are used to understand and solve community problems, often by an external actor. **‘Community capacity development’** is process oriented. External actors aim to enable the community to understand their own problems and implement their own solutions. **‘Social advocacy’** is task and process oriented, with external actors further galvanising the community to redress systematic power imbalances in pursuit of equity and justice. |
| Rifkin and Pridmore [6]  Draper et al. [7] | Differentiates community participation based on the perspective on health and respective role of the community in health programmes. **‘Information giving’** and **‘consultation’** or **‘mobilisation’** views health as the absence of disease and external actors provide advice to the community as experts. **‘Collaboration’** incorporates a broader perspective of health as physical, mental, and social wellbeing, with the community contributing time and resources towards externally defined health programmes. **‘Empowerment’** further defines health as the human condition. The community plans and implement health programmes and external actors function as facilitators. |

## Text B. Eligibility criteria and search strategy for database searches

**Eligibility criteria**

**Table. Inclusion and exclusion criteria**

|  | **Inclusion** | **Exclusion** |
| --- | --- | --- |
| Article | Full text, peer-reviewed articles | Abstracts |
| Language | English | Non-English |
| Study design | Cluster RCT or economic evaluations using RCT data | Commentaries, meta-analyses, observational studies, non-randomised intervention studies, economic evaluations using observational data, protocols, reviews, meta-analyses  Interim or pilot RCTs, individual RCTs, RCTs with 1 group per arm, RCTs with interventions added post-randomisation |
| Disease area | CDs or determinants of CDs | Other diseases (e.g., non-CDs, diseases caused by infectious agents but not spread from person-to-person)  Oher diseases among people living with chronic CDs  Determinants of CDs but disease not described or described generally (e.g., infection) |
| Outcome | Effects, costs, and cost-effectiveness related to CDs or determinants of CDs | Effects, costs, and cost-effectiveness related to other diseases |
| Population | Any population | Not applicable |
| Intervention, setting | Outside of standard health facilities | Standard health facilities, laboratories, pharmacies |
| Intervention, group | Groups, organisations, or networks with shared spatial or social characteristics or collective interests | Non-groups, organisations, or networks  Groups, organisations, or networks without shared spatial or social characteristics or collective interests, or not specified |
| Intervention, participation | The community is an agent. Communities define problems and implement and evaluate solutions, with external actors providing support as facilitators. Decisions are mostly made by the community. | The community is a setting. External actors define problems and implement and evaluate solutions, with communities excluded from making contributions and decisions.  The community is a target. Communities input into defining problems and implementing and evaluating solutions, but decisions are mostly made by external actors.  The community is a resource. Communities define problems and implement and evaluate solutions in partnership with external actors. Decisions are made jointly or shared. |
| Comparator | Any comparator | Not applicable |

CD, communicable disease; RCT, randomised controlled trial.

**Search strategy**

Seven electronic databases were searched on 11 October, 2021. Searches were updated through 31 December, 2023.

**Cochrane Trials**

|  | **Search** | **N** |
| --- | --- | --- |
| 1 | (community consultation OR community collaboration OR community directed OR community-directed OR community driven OR community-driven OR community empowerment OR community led OR community-led OR community mobili$ation OR community action OR community capacity building OR community development OR community engagement OR community initiative OR community involvement OR community organi$ation OR community outreach OR community participation) in All Text | 48,780 |
| 2 | (coronavirus OR covid OR hepatitis OR human immunodeficiency virus OR HIV OR sexually transmitted OR sexually-transmitted OR STIs OR STDs OR tuberculosis OR TB OR vector* OR mmunee* OR malaria OR dengue OR chikungunya OR zika OR neglected tropical diseases OR NTDs OR lymphatic filariasis OR onchocerciasis OR schistosomiasis OR trachoma OR soil transmitted helminth* OR soil-transmitted helminth* OR STHs OR immune$ation OR infect* OR transmit* OR communicable OR viral OR virus* OR bacteri*) in All Text | 303,242 |
| 3 | (random* OR trial* OR experiment* OR cost*) in Abstract | 1,308,765 |
| 4 | #1 AND #2 AND #3 | 10,863 |

**Econlit**

|  | **Search** | **N** |
| --- | --- | --- |
| 1 | (community consultation or community collaboration or community directed or community-directed or community driven or community-driven or community empowerment or community led or community-led or community mobili$ation or community action or community capacity building or community development or community engagement or community initiative or community involvement or community organi$ation or community outreach or community participation).af. | 2,996 |
| 2 | (coronavirus or covid or hepatitis or human immunodeficiency virus or HIV or sexually transmitted or sexually-transmitted or STIs or STDs or tuberculosis or TB or vector* or parasit* or malaria or dengue or chikungunya or zika or neglected tropical diseases or NTDs or lymphatic filariasis or onchocerciasis or schistosomiasis or trachoma or soil transmitted helminth* or soil-transmitted helminth* or STHs or immuni$ation or infect* or transmit* or communicable or viral or virus* or bacteri*).af. | 41,726 |
| 3 | (random* or trial* or experiment* or cost*).ab. | 234,649 |
| 4 | 1 and 2 and 3 | 11 |

**EMBASE**

|  | **Search** | **N** |
| --- | --- | --- |
| 1 | (community consultation or community collaboration or community directed or community-directed or community driven or community-driven or community empowerment or community led or community-led or community mobili$ation or community action or community capacity building or community development or community engagement or community initiative or community involvement or community organi$ation or community outreach or community participation).af. | 30,449 |
| 2 | (coronavirus or covid or hepatitis or human immunodeficiency virus or HIV or sexually transmitted or sexually-transmitted or STIs or STDs or tuberculosis or TB or vector* or parasit* or malaria or dengue or chikungunya or zika or neglected tropical diseases or NTDs or lymphatic filariasis or onchocerciasis or schistosomiasis or trachoma or soil transmitted helminth* or soil-transmitted helminth* or STHs or immuni$ation or infect* or transmit* or communicable or viral or virus* or bacteri*).af. | 7,286,087 |
| 3 | (random* or trial* or experiment* or cost*).ab. | 6,275,599 |
| 4 | 1 and 2 and 3 | 1,888 |

**Global Health**

|  | **Search** | **N** |
| --- | --- | --- |
| 1 | (community consultation or community collaboration or community directed or community-directed or community driven or community-driven or community empowerment or community led or community-led or community mobili$ation or community action or community capacity building or community development or community engagement or community initiative or community involvement or community organi$ation or community outreach or community participation).af. | 22,721 |
| 2 | (coronavirus or covid or hepatitis or human immunodeficiency virus or HIV or sexually transmitted or sexually-transmitted or STIs or STDs or tuberculosis or TB or vector* or parasit* or malaria or dengue or chikungunya or zika or neglected tropical diseases or NTDs or lymphatic filariasis or onchocerciasis or schistosomiasis or trachoma or soil transmitted helminth* or soil-transmitted helminth* or STHs or immuni$ation or infect* or transmit* or communicable or viral or virus* or bacteri*).af. | 3,248,655 |
| 3 | (random* or trial* or experiment* or cost*).ab. | 864,213 |
| 4 | 1 and 2 and 3 | 2,064 |

**Medline**

|  | **Search** | **N** |
| --- | --- | --- |
| 1 | (community consultation or community collaboration or community directed or community-directed or community driven or community-driven or community empowerment or community led or community-led or community mobili$ation or community action or community capacity building or community development or community engagement or community initiative or community involvement or community organi$ation or community outreach or community participation).af. | 38,146 |
| 2 | (coronavirus or covid or hepatitis or human immunodeficiency virus or HIV or sexually transmitted or sexually-transmitted or STIs or STDs or tuberculosis or TB or vector* or parasit* or malaria or dengue or chikungunya or zika or neglected tropical diseases or NTDs or lymphatic filariasis or onchocerciasis or schistosomiasis or trachoma or soil transmitted helminth* or soil-transmitted helminth* or STHs or immuni$ation or infect* or transmit* or communicable or viral or virus* or bacteri*).af. | 5,710,325 |
| 3 | (random* or trial* or experiment* or cost*).ab. | 4,766,352 |
| 4 | 1 and 2 and 3 | 1,595 |

**Pub Med**

|  | **Search** | **N** |
| --- | --- | --- |
| 1 | (“community consultation” OR “community collaboration” OR “community directed” OR “community-directed” OR “community driven” OR “community-driven” OR “community empowerment” OR “community led” OR “community-led” OR “community mobilization” OR “community mobilisation” OR “community action” OR “community capacity building” OR “community development” OR “community engagement” OR “community initiative” OR “community involvement” OR “community organization” OR “community organisation” OR “community outreach” OR “community participation”) in All Fields | 42,738 |
| 2 | (coronavirus OR covid OR hepatitis OR “human immunodeficiency virus” OR HIV OR “sexually transmitted” OR “sexually-transmitted” OR STIs OR STDs OR tuberculosis OR TB OR vector* OR parasit* OR malaria OR dengue OR chikungunya OR zika OR “neglected tropical diseases” OR NTDs OR “lymphatic filariasis” OR onchocerciasis OR schistosomiasis OR trachoma OR “soil transmitted helminth*” OR “soil-transmitted helminth*” OR STHs OR immunization OR immunisation OR infect* OR transmit* OR communicable OR viral OR virus* OR bacteri*) in All Fields | 6,635,605 |
| 3 | (random* OR trial* OR experiment* OR cost*) in Title/Abstract | 5,274,395 |
| 4 | #1 AND #2 AND #3 | 1,969 |

**Web of Science**

|  | **Search** | **N** |
| --- | --- | --- |
| 1 | ALL=(“community consultation” OR “community collaboration” OR “community directed” OR “community-directed” OR “community driven” OR “community-driven” OR “community empowerment” OR “community led” OR “community-led” OR “community mobili?ation” OR “community action” OR “community capacity building” OR “community development” OR “community engagement” OR “community initiative” OR “community involvement” OR “community organi?ation” OR “community outreach” OR “community participation”) | 52,557 |
| 2 | ALL=(coronavirus OR covid OR hepatitis OR “human immunodeficiency virus” OR HIV OR “sexually transmitted” OR “sexually-transmitted” OR STIs OR STDs OR tuberculosis OR TB OR vector* OR parasit* OR malaria OR dengue OR chikungunya OR zika OR “neglected tropical diseases” OR NTDs OR “lymphatic filariasis” OR onchocerciasis OR schistosomiasis OR trachoma OR “soil transmitted helminth*” OR “soil-transmitted helminth*” OR STHs OR immuni?ation OR infect* OR transmit* OR communicable OR viral OR virus* OR bacteri*) | 6,944,012 |
| 3 | AB=(random* OR trial* OR experiment* OR cost*) | 10,911,482 |
| 4 | #1 AND #2 AND #3 | 1,691 |

## Text C. Data extraction form

| **Group** | **Category** | **Field** |
| --- | --- | --- |
| Record information | Record characteristics | Author |
|  |  | Year |
|  |  | Record category (primary/secondary/economic) |
|  |  | Record title |
| Parent | Parent characteristics | Country |
|  |  | Region |
|  |  | Population |
|  |  | Disease areas - communicable diseases |
|  |  | Disease areas - other |
|  | Parent design | Randomised trial design |
|  |  | Randomisation unit |
|  |  | Number of units |
|  |  | Unit eligibility |
|  |  | Intervention details |
|  |  | Control details |
| Intervention | Intervention and control characteristics | External actors |
|  |  | Community actors |
|  |  | Other actors |
|  |  | Intervention setting |
|  |  | Intervention eligibility |
|  |  | Intervention period |
|  |  | Strategies for community participation |
|  |  | Strategies for communicable diseases |
|  | Community participation | Design - score (information giving / consultation / collaboration / empowerment) |
|  |  | Design - details |
|  |  | Implementation - score (information giving / consultation / collaboration / empowerment) |
|  |  | Implementation - details |
|  |  | Monitoring and evaluation - score (information giving / consultation / collaboration / empowerment) |
|  |  | Monitoring and evaluation - details |
|  |  | Post-implementation - score (information giving / consultation / collaboration / empowerment) |
|  |  | Post-implementation - details |
| Outcome evaluation | Study design | Data sources |
|  |  | Measurement timepoints |
|  |  | Measurement period |
|  |  | Sampling approach |
|  |  | Sample |
|  |  | Sample size |
|  |  | Analytical approach |
|  | Results | Primary outcomes - summary |
|  |  | Primary outcomes - measure |
|  |  | Primary outcomes - estimate |
|  |  | Secondary health / health care outcomes - summary |
|  |  | Secondary health / health care outcomes - measure |
|  |  | Secondary health / health care outcomes - estimate |
|  |  | Other secondary outcomes - summary |
|  |  | Other secondary outcomes - measure |
|  |  | Other secondary outcomes - estimate |
| Economic evaluation | Study design | Study design |
|  |  | Perspective |
|  |  | Time horizon |
|  |  | Analytical approach |
|  |  | Sensitivity analysis |
|  |  | Discount rate |
|  |  | Inflation rate |
|  | Results | Currency |
|  |  | Currency year |
|  |  | Incremental cost-effectiveness ratio estimate |
|  |  | Incremental cost-effectiveness ratio estimate (adjusted) |
|  |  | Cost-effectiveness probability |
| Cost | Study design | Study design |
|  |  | Perspective |
|  |  | Prospective or retrospective |
|  |  | Economic or financial |
|  |  | Real world or per protocol |
|  |  | Full or incremental |
|  |  | Scope (above service delivery / service delivery / community / patient) |
|  |  | Inputs |
|  |  | Data sources |
|  |  | Measurement period |
|  |  | Sampling approach |
|  |  | Sample size |
|  | Results | Currency |
|  |  | Currency year |
|  |  | Total cost estimate |
|  |  | Output estimate |
|  |  | Unit cost estimate |
|  |  | Unit cost estimate (adjusted) |
|  |  | Direct costs (%) |
|  |  | Indirect costs (%) |
|  |  | Start-up (%) |
|  |  | Capital (%) |
|  |  | Personnel (%) |
|  |  | Other recurrent (%) |
|  |  | Other (%) |
| Process evaluation | Study design | Study design |
|  |  | Sampling approach |
|  |  | Sample |
|  |  | Sample size |
|  |  | Analytical approach |
|  | Results | Implementation details |
|  |  | Mechanism of impact details |
|  |  | Context details |
| Risk of bias | Risk of bias tool for cluster randomised trials [8] | Randomisation |
|  |  | Timing of identification or recruitment of participants |
|  |  | Deviations from intended interventions (assignment) |
|  |  | Missing outcome data |
|  |  | Measurement of the outcome |
|  |  | Selection of the reported result |
|  |  | Overall (low / moderate / high) |
|  | Drummond checklist for critique of economic evaluations [9] | Was a well-defined question posed in answerable form? |
|  |  | Was a comprehensive description of the competing alternatives given (i.e., can you tell who did what to whom, where, and how often)? |
|  |  | Was the effectiveness of the programme or services established? |
|  |  | Were all the important and relevant costs and consequences for each alternative identified? |
|  |  | Were costs and consequences measured accurately in appropriate physical units (e.g., hours of nursing time, number of physician visits, lost workdays, gained life years)? |
|  |  | Were the cost and consequences valued credibly? |
|  |  | Were costs and consequences adjusted for differential timing? |
|  |  | Was an incremental analysis of costs and consequences of alternatives performed? |
|  |  | Was allowance made for uncertainty in the estimates of costs and consequences? |
|  |  | Did the presentation and discussion of study results include all issues of concern to users? |
|  |  | Overall (low / moderate / high) |

## Text D. Adapted framework for community participation

|  | **Information giving** | **Consultation** | **Collaboration** | **Empowerment** |
| --- | --- | --- | --- | --- |
| **Overall** | The community is a setting. External actors define problems and implement and evaluate solutions, with communities excluded from making contributions and decisions. | The community is a target. Communities input into defining problems and implementing and evaluating solutions, but contributions and decisions are mostly made by external actors. | The community is a resource. Communities define problems and implement and evaluate solutions in partnership with external actors. Contributions and decisions are made jointly or shared. | The community is an agent. Communities define problems and implement and evaluate solutions, with external actors providing support as facilitators. Contributions and decisions are mostly made by the community. |
| **Design** | External actors identify priorities, design strategies, and prepare funds and resources, with communities excluded from making contributions and decisions. | Communities input into identifying priorities, designing strategies, and preparing funds and resources, but contributions and decisions are mostly made by external actors. | Communities identify priorities, design strategies, and prepare funds and resources in partnership with external actors. Contributions and decisions are made jointly or shared. | Communities identify priorities, design strategies, and prepare funds and resources, with external actors providing support as facilitators. Contributions and decisions are mostly made by the community. |
| **Implementation** | External actors mobilise funds and resources and implement strategies, with communities excluded from making contributions and decisions. | Communities contribute towards mobilising funds and resources and implementing strategies, but contributions and decisions are mostly made by external actors. | Communities mobilise funds and resources and implement strategies in partnership with external actors. Contributions and decisions are made jointly or shared. | Communities mobilise funds and resources and implement strategies, with external actors providing support as facilitators. Contributions and decisions are mostly made by the community. |
| **Monitoring and evaluation** | External actors define data indicators, collect and analyse data, and discuss learnings, with communities excluded from making contributions and decisions. | Communities contribute towards defining data indicators, collecting and analysing data, and discussing learnings, but contributions and decisions are mostly made by external actors. | Communities define data indicators, collect and analyse data, and discuss learnings in partnership with external actors. Contributions and decisions are made jointly or shared. | Communities define data indicators, collect and analyse data, and discuss learnings, with external actors providing support as facilitators. Contributions and decisions are mostly made by the community. |
| **Post-implementation** | External actors define long-term priorities, strategies, and funds and resources, with communities excluded from making contributions and decisions. | Communities contribute towards defining long-term priorities, strategies, and funds and resources, but contributions and decisions are mostly made by external actors. | Communities define long-term priorities, strategies, and funds and resources in partnership with external actors. Contributions and decisions are made jointly or shared, | Communities define long-term priorities, strategies, and funds and resources, with external actors providing support as facilitators. Contributions and decisions are mostly made by the community. |

Adapted from Rifkin and Pridmore (2001) and Draper (2010) [6, 7].

## Table A. Results for community participation scores

| **Article** | **Setting** | **Intervention** | **Community unit** | **Main strategies for community participation** | **Main strategies for communicable diseases** | **Intervention period** | **Design** | **Implementation** | **M&E** | **Post-implementation** | **Overall** |  |
| --- | --- | --- | --- | --- | --- | --- | --- | --- | --- | --- | --- | --- |
| **Diarrhoeal diseases** | |  |  |  |  |  |  |  |  |  |  |  |
| Biran (2018) [10] | Malawi | CLTS inclusive of people with disabilities | Sanitation committees | Coalition building  Situational assessment  Action planning | Awareness raising  Education  Environmental alterations | 7 months | 3 | 4 | 3 | 0 | 10 |  |
| Briceño (2017) [11] | Tanzania | CLTS | Sanitation committees | Coalition building  Situational assessment  Action planning | Awareness raising  Education  Environmental alterations | 2 years | 3 | 4 | 4 | 0 | 11 |  |
| Cameron (2019) [12] | Indonesia | CLTS | Community members | Coalition building  Situational assessment  Action planning | Awareness raising  Education  Environmental alterations | NR | 3 | 4 | 3 | 0 | 10 |  |
| Cha (2021) [13] | Ethiopia | CLTS | WASH promoters | Coalition building  Situational assessment  Action planning  Skills development | Awareness raising  Education  Environmental alterations | 1 year | 3 | 4 | 3 | 0 | 10 |  |
| Crocker (2016) [14] | Ghana | CLTS with training of natural leaders | Natural leaders, community members | Coalition building  Situational assessment  Action planning  Skills development | Awareness raising  Education  Environmental alterations | 1.5 years | 3 | 4 | 0 | 0 | 7 |  |
| Pickering (2015) [15] | Mali | CLTS | Sanitation committees | Coalition building  Situational assessment | Awareness raising  Education  Environmental | 2 years | 3 | 4 | 3 | 0 | 10 |  |
|  |  |  |  | Action planning | alterations |  |  |  |  |  |  |  |
| Quattrochi (2018) [16] | Democratic Republic of Congo | Community-led WASH | WASH committees, WASH volunteers | Coalition building  Problem assessment and solving  Action planning  Skills development | Awareness raising  Education  Environmental alterations | 6 months | 4 | 4 | 3 | 3 | 14 |  |
| **HIV** |  |  |  |  |  |  |  |  |  |  |  |  |
| Abramsky (2014) [17] | Uganda | Community mobilisation for HIV and IPV prevention | Community activists | Coalition building  Problem assessment and solving  Action planning  Skills development | Awareness raising  Education  Peer support | 4 years | 4 | 4 | 0 | 3 | 11 |  |
| Indravudh (2021) [18] | Malawi | Community-led HIVST | Community health action groups and volunteers | Coalition building  Problem assessment and solving  Action planning  Goal setting and review  Skills development | Demand creation  Diagnostic testing | 2 weeks | 4 | 4 | 4 | 0 | 12 |  |
| Sibanda (2021) [19] | Malawi | Community-led HIVST | Community leaders, distributors, and members | Coalition building  Problem assessment and solving  Action planning  Skills development | Demand creation  Diagnostic testing | 6 weeks | 4 | 4 | 3 | 0 | 11 |  |
| **Malaria** |  |  |  |  |  |  |  |  |  |  |  |  |
| McCann (2021) [20] | Malawi | Community-driven larval source management and house improvement. | Village committees, health animators | Coalition building  Action planning  Goal setting and review  Skills development | Vector control | 2.5 years | 3 | 4 | 3 | 0 | 10 |  |
| **Neglected tropical diseases** | |  |  |  |  |  |  |  |  |  |  | |
| Andersson (2015) [21] | Mexico, Nicaragua | Community-led dengue control | Community groups, volunteers | Coalition building  Problem assessment and solving  Action planning  Skills development | Education  Environmental alterations  Vector control | 1.5 years | 4 | 4 | 3 | 0 | 11 | |
| Massa (2009) [22] | Tanzania | Community-directed distribution of treatment for schistosomiasis and soil-transmitted helminthiasis | Community drug distributors, members | Coalition building  Action planning  Skills development | Treatment | 1 year | 4 | 4 | 0 | 0 | 8 | |
| **Multiple diseases** | |  |  |  |  |  |  |  |  |  |  | |
| Lewycka (2013) [23] | Malawi | Participatory women’s groups for maternal and child health | Women’s groups | Coalition building  Problem assessment and solving  Action planning  Goal setting and review  Skills development | Education  Access to services  Income generation  Nutrition  Vector control | 3.5 years | 4 | 4 | 4 | 4 | 16 | |
| Makaula (2019) [24] | Malawi | Community-directed primary health care | Community volunteers, members | Coalition building  Problem assessment and solving  Action planning  Skills development | Vector control  Drugs for prevention  Drugs for treatment  Nutrition | 1 year | 4 | 4 | 3 | 0 | 11 | |
| Nair (2017) [25] | India | Participatory women’s groups  for maternal and  child health | Women’s groups | Coalition building  Problem assessment and solving  Action planning  Goal setting and review  Skills development | Education  Peer support  Advocacy  Access to services  Nutrition | 2 years | 4 | 4 | 4 | 0 | 12 | |

CLTS, community-led total sanitation; HIVST, HIV self-testing; IPV, intimate partner violence; NR, not reported; WASH, water, sanitation, and hygiene. Scores use a 0–4 scale: 0=not reported, 1=information giving, 2=consultation, 3=collaboration, 4=empowerment.

## Table B. Risk of bias assessment for cluster-randomised trials

|  | **1a. Randomisation** | **1b. Timing of identification or**  **recruitment of participants** | **2. Deviations from intended**  **interventions** | **3. Missing outcome data** | **4. Measurement of outcome** | **5. Selection of reported result** | **Overall** |
| --- | --- | --- | --- | --- | --- | --- | --- |
| **Diarrhoeal diseases** |  |  |  |  |  |  |  |
| Biran (2018) [10] | × | + | – | × | + | – | × |
| Briceño (2017) [11] | – | + | – | + | – | + | – |
| Cameron (2019) [12] | – | + | – | × | – | – | × |
| Cha (2021) [13] | – | + | – | + | – | + | – |
| Crocker (2016) [14] | + | + | – | + | – | – | – |
| Pickering (2015) [15] | – | + | – | + | – | + | – |
| Quattrochi (2018) [16] | + | + | – | + | – | + | – |
| **HIV** |  |  |  |  |  |  |  |
| Abramsky (2014) [17] | + | + | – | + | – | + | – |
| Indravudh (2021) [18] | + | + | – | + | – | + | – |
| Sibanda (2021) [19] | + | + | – | + | – | + | – |
| **Malaria** |  |  |  |  |  |  |  |
| McCann (2021) [20] | – | + | – | × | + | + | × |
| **Neglected tropical diseases** | |  |  |  |  |  |  |
| Andersson (2015) [21] | + | + | + | + | + | + | + |
| Massa (2009) [22] | – | + | – | × | + | – | × |
| **Multiple diseases** |  |  |  |  |  |  |  |
| Lewycka (2013) [23] | + | + | – | + | + | + | – |
| Makaula (2019) [24] | – | + | – | × | – | – | × |
| Nair (2017) [25] | + | + | – | + | + | + | – |

+, low risk; –, moderate risk; ×, high risk. Risk of bias assessment used the Revised Chochrane Risk-of-Bias Tool for cluster-randomised trials [8].

## Table C. Risk of bias assessment for economic evaluations

|  | **1. Was a well-defined question posed in answerable form?** | **2. Was a comprehensive description of the competing alternatives given?** | **3. Was the effectiveness of the programme or services established?** | **4. Were all the important and relevant costs and consequences for each alternative identified?** | **5. Were costs and consequences measured accurately in appropriate .physical units?** | **6. Were the cost and consequences valued credibly?** | **7. Were costs and consequences adjusted for**  **differential timing?** | **8. Was an incremental analysis of costs and consequences of alternatives performed?** | **9. Was allowance made for uncertainty in the estimates of costs and consequences?** | **10. Did the presentation and discussion of study results include all issues of concern to users?** | **Overall** |
| --- | --- | --- | --- | --- | --- | --- | --- | --- | --- | --- | --- |
| **Diarrhoeal diseases** |  |  |  |  |  |  |  |  |  |  |  |
| Briceño (2017) [11, 26] | + | + | + | + | + | – | – | + | × | × | × |
| Cha (2021) [13, 27] | + | + | + | + | – | + | – | + | + | – | – |
| Crocker (2016) | + | + | + | + | + | × | + | + | – | – | × |
| **HIV** |  |  |  |  |  |  |  |  |  |  |  |
| Indravudh (2021)  [18, 28] | + | + | + | × | + | – | – | + | – | – | × |
| **Neglected tropical diseases** | |  |  |  |  |  |  |  |  |  |  |
| Andersson (2015)  [21, 29] | + | – | + | × | + | – | – | + | – | – | × |
| **Multiple diseases** |  |  |  |  |  |  |  |  |  |  |  |
| Lewycka (2013) [23] | + | – | + | × | – | + | – | + | × | × | × |
| Nair (2017) [25] | + | – | + | × | + | – | – | + | × | × | × |

+, low risk; –, moderate risk; ×, high risk. Risk of bias assessment used the Drummond checklist for economic evaluations [9].

## Table D. Results from cluster-randomised trials

| **Article** | **Study design** | **Setting** | **Intervention** | **Control** | **Sample size** | **Effect estimates** |
| --- | --- | --- | --- | --- | --- | --- |
| **Diarrhoeal diseases** | |  |  |  |  |  |
| Biran (2018) [10] | CRT of group village head units | Malawi | CLTS inclusive of people with disabilities | CLTS | Household, with people with disabilities,  *N* = 171 | **Behaviour**  **Null:** latrine construction*, improved latrine construction, time to travel to latrine, able to use latrine as required, able to use latrine without assistance, water near latrine for handwashing, able to use latrine without coming into contact with faeces, improved latrine access for people with disabilities, easy latrine access, wants changes to latrine  **Other**  **Positive:** meeting attendance, meeting attendance among people with disabilities, discussed sanitation, discussed sanitation among people with disabilities, discussed how to make latrine access easier, discussed how to make latrine access easier among people with disabilities, invited to participate in sanitation activities |
| Briceño (2017) [11] | Factorial CRT of wards | Tanzania | CLTS  CLTS and handwashing promotion | Handwashing promotion  No intervention | Household, with children <5 years,  *N* = 3,619 | **Mortality and morbidity**  **Negative:** 14-day diarrhoeal prevalence (CLTS+HW vs. C), haemoglobin levels (CLTS+HW vs. C), weight-for-age (CLTS+HW vs. C)  **Null:** 7-day diarrhoeal prevalence (CLTS vs. C)*, 7-day diarrhoeal prevalence (CLTS+HW vs. C)*, 14-day diarrhoeal prevalence (CLTS vs. C), health index (CLTS vs. C), health index (CLTS+HW vs. C), haemoglobin levels (CLTS vs. C), weight-for-age (CLTS vs. C), height-for-age (CLTS vs. C), height-for-age (CLTS+HW vs. C), head circumference (CLTS vs. C), head circumference (CLTS+HW vs. C)  **Behaviour**  **Positive:** sanitation index (CLTS vs. C), sanitation index (CLTS+HW vs. C), latrine construction (CLTS vs. C), latrine construction (CLTS+HW vs. C), improved latrine use (CLTS vs. C), improved latrine use (CLTS+HW vs. C), safe removal of child faeces (CLTS vs. C), safe removal of child faeces |
|  |  |  |  |  |  | (CLTS+HW vs. C), open defecation free village (CLTS vs. C), open defecation free village (CLTS+HW vs. C), hygiene index (CLTS+HW vs. C), knowledge of handwashing (CLTS+HW vs. C), has a fixed  handwashing device (CLTS+HW vs. C), handwashing before handling food (CLTS+HW vs. C), caregiver hand cleanliness index (CLTS+HW vs. C), child cleanliness index (CLTS+HW vs. C)  **Negative:** open defecation (CLTS vs. C), open defecation (CLTS+HW vs. C), handwashing after faecal contact (CLTS vs. C)  **Null:** hygiene index (CLTS vs. C), knowledge of handwashing (CLTS vs. C), has a handwashing device (CLTS vs. C), has a handwashing device (CLTS+HW vs. C), has a fixed handwashing device (CLTS vs. C), handwashing after faecal contact (CLTS+HW vs. C), handwashing before handling food (CLTS vs. C), caregiver hand cleanliness index (CLTS vs. C), child cleanliness index (CLTS vs. C) |
| Cameron (2019) [12] | CRT of villages | Indonesia | CLTS | No intervention | Household,  *N* *=* 1,858 | **Mortality and morbidity**  **Negative:** roundworm density  **Null:** haemoglobin levels, weight z-score, height z-score, health index  **Behaviour**  **Positive:** latrine construction, open defecation intolerance  **Null:** diarrhoeal knowledge |
| Cha (2021) [13] | CRT of villages | Ethiopia | CLTS | SOC | Household, with children <5 years, *N* = 842 | **Mortality and morbidity**  **Negative:** diarrhoeal incidence*,100-day diarrhoeal prevalence*  **Null:** diarrhoeal duration*, 7-day diarrhoeal prevalence*  **Behaviour**  **Positive:** own toilet, own improved toilet, own partially improved toilet or better, own hand washing facility, self-reported toilet use  **Negative:** faeces in compound, faeces outside compound, presence of flies  **Null:** observed toilet use, faeces around pit hole, child faeces disposal, handwashing |
| Crocker (2016) [14, 30] | CRT of villages | Ghana | CLTS with training of natural leaders | CLTS | Households, *N* = 1,708 | **Behaviour**  **Positive:** shared latrine ownership*, private latrine ownership*, latrine use  **Negative:** open defecation*  **Null:** communal latrine ownership* |
| Pickering (2015) [15] | CRT of villages | Mali | CLTS | No intervention | Household, with children <10 years, *N* = 4,031 | **Mortality and morbidity**  **Positive:** Height-for-age z-score among children <5 years, height-for-age z-score among children <2 years, height-for-age z-score among children <1 year, weight-for-age z-score among children <2 years, weight-for-age z-score among children <1 year  **Negative:** stunted among children <5 years, stunted among children <2 years, stunted among children <1 year, severely stunted among children <5 years, severely stunted among children <2 years, severely stunted among children <1 year, underweight among children <2 years, underweight among children <1 year, severely underweight among children <5 years, severely underweight among children <2 years, severely underweight among children <1 year, blood in stool at 2-week recall, difficulty breathing at 2-day recall, difficulty breathing at 2-week recall, diarrhoea-related mortality  **Null:** diarrhoea at 2-day recall*, diarrhoea at 2-week recall*, weight-for-age z-score among children <5 years, underweight among children <5 years, loose stool at 2-day recall, loose stool at 2-week recall, blood in stool at 2-day recall, vomiting at 2-day recall, vomiting at 2-week recall, fever at 2-day recall, fever at 2-week recall, congestion at 2-day recall, congestion at 2-week recall, cough at 2-day recall, cough at 2-week recall, earache at 2-day recall, bruising at 2-day recall, mortality  **Behaviour**  **Positive:** access to own latrine, child uses potty, satisfied with sanitation, women have privacy, women feel safe at night, potty in latrine, soap in latrine, water in latrine, latrine hole covered,  stored water reported treated, daily handwashes with soap, reports handwashing important after using toilet  **Negative:** share latrine with other households, flies in latrine, human faeces in compound, animal faeces in compound, open |
|  |  |  |  |  |  | defecation among women, open defecation among men, open defecation among children 5–10 years, open defecation among children <5 years  **Null:** latrine has concrete slab, faeces on latrine floor, water or urine on latrine floor, clear path to latrine, latrine appears used, mother has clean palms  **Environment**  **Null:** E. coli per 100 mL in stored water, E. coli per 100 mL in source water |
| Quattrochi (2018) [16, 31] | CRT of village groups | Democratic Republic of Congo | Community-led WASH | No intervention | Individual, women, *N* = 1,312 | **Mortality and morbidity**  **Null:** child health index, people with COVID-19 symptoms, people with non COVID-19 symptoms, psychological well-being index, quantity of household members with illnesses  **Health care access and utilisation**  **Null:** quantity of hospital visits, forgone visits for health care  **Behaviour**  **Positive:** improved water source*, improved sanitation facility*, handwashing index, sanitation index, water satisfaction index, vaccine acceptance, household COVID prevention index, perception of COVID prevention index, COVID knowledge index  **Null:** time to collect water*, quantity of water collected*, water storage index  **Community and social**  **Positive:** village COVID prevention index, vaccine acceptance by village leaders, vaccine advice by village leader  **Other**  **Null:** school attendance index, financial cost of water, water quality and access index, water governance index, governance index, livelihood index, food insecurity, approval of President, approval of National Assembly, approval of National Ministry of Health, approval of Provincial Government, approval of international NGOs, approval of local NGOs, approval of |
|  |  |  |  |  |  | traditional leaders, approval of health zone officials, approval of health area officials, approval of village chief |
| **HIV** |  |  |  |  |  |  |
| Abramsky (2014) [17, 32, 33] | Pair-matched CRT of administrative parishes | Uganda | Community mobilisation for HIV and IPV prevention | Enhanced SOC | Individual, 18–49 years, *N* = 2,532 | **Health care access and utilisation**  **Positive:** HIV testing among men  **Null:** HIV testing among women  **Behaviour**  **Positive:** acceptance of refusal to have sex among women*, acceptance of refusal to have sex among men*, discussed HIV testing with partner among men, ability to refuse sex with partner, discussed condom use among men, condom use at last sex among men, joint decision making with partner among women, joint decision making with partner among men, helps partner with housework among men, helps partner look after children among men, appreciation for work partner does inside home among men, appreciation for work partner does outside home among men, discussed planning for children with partner among men, discussed partner's sexual preferences among men, discussed sexual preferences with partner among men, discussed feelings with partner among men  **Negative:** acceptance of physical IPV among women*, concurrency of sexual partners among men*, continued physical IPV, continued sexual IPV, continued physical/sexual IPV, any emotional IPV, high intensity emotional IPV, continued emotional aggression, high-intensity controlling behaviours, new controlling behaviours, continued fear of partner  **Null:** acceptance of physical IPV among men*, physical IPV*, sexual IPV*, discussed condom use among women, condom use among women, condom use among men, condom use at last sex among women, concurrency of sexual partners among women, discussed HIV testing with partner among women, partner helps with housework among women, partner helps look after children among women, appreciation for work partner does inside home among women, appreciation for work partner does outside home among women, discussed planning for children with partner among women, discussed partner's sexual preferences among women, discussed sexual preferences with partner among |
|  |  |  |  |  |  | women, discussed day with partner among women, discussed day with partner among men, discussed feelings with partner among women, at least one episode of severe physical IPV or more than one occurrence of less severe act, new onset physical IPV, new sexual IPV, physical/sexual IPV, injury from physical/sexual IPV, new physical/sexual IPV, new emotional aggression, any controlling behaviours, continued controlling behaviours, fear of partner, new fear of partner  **Community and social**  **Null:** community response to women experiencing IPV* |
| Indravudh (2021) [18, 34] | CRT of group village head units | Malawi | Community-led HIVST | SOC | Individual, ≥15 years,  *N* = 7,880  Population, ≥15 years,  *N* = 84,349^1^ | **Health care access and utilisation**  **Positive:** lifetime HIV testing among adolescents*, HIV testing among adults ≥40 years, HIV testing among men  **Null:** antiretroviral therapy initiation  **Behaviour**  **Null:** knowledge of HIV treatment benefits  **Community and social**  **Positive:** social cohesion, shared concern for HIV  **Null:** HIV testing stigma, community HIV stigma, critical consciousness |
| Sibanda (2021) [19, 35] | CRT of village headman units | Zimbabwe | Community-led HIVST | Community-based HIVST | Individual, ≥16 years, *N* = 11,510 | **Mortality and morbidity**  **Null:** New HIV diagnosis*  **Health care access and utilization**  **Null:** Linkage to confirmatory testing, pre-exposure prophylaxis, and voluntary medical male circumcision*, |
| **Malaria** |  |  |  |  |  |  |
| McCann (2021) [20, 36] | Factorial CRT of village groups | Malawi | Community-driven house improvement  Community-driven larval source management | SOC | Household,  *N* = 1,844 | **Mortality and morbidity**  **Null:** malaria prevalence without symptoms among women (HI vs. C), malaria prevalence without symptoms among women (LSM vs. C), malaria prevalence without symptoms among women (HI+LSM vs. C), malaria prevalence without symptoms among children 6–23 months (HI vs. C), malaria prevalence without symptoms among children 6–23 months (LSM vs. C), malaria prevalence without symptoms among children 6–23 |
|  |  |  | Community-driven house improvement and larval source management |  |  | months (HI+LSM vs. C), malaria prevalence without symptoms among children 6-59 months (HI vs. C), malaria prevalence without symptoms among children 6–59 months (LSM vs. C), malaria prevalence without symptoms among children 6–59 months (HI+LSM vs. C), malaria prevalence with symptoms among women (HI vs. C), malaria prevalence with symptoms among women (LSM vs. C), malaria prevalence with symptoms among women (HI+LSM vs. C), malaria prevalence with symptoms among children 6–23 months (HI vs. C), malaria prevalence with symptoms among children 6–23 months (LSM vs. C), malaria prevalence with symptoms among children 6–23 months (HI+LSM vs. C), malaria prevalence with symptoms among children 6–59 months (HI vs. C), malaria prevalence with symptoms among children 6–59 months (LSM vs. C), malaria prevalence with symptoms among children 6–59 months (HI+LSM vs. C), haemoglobin levels among women (HI vs. C), haemoglobin levels among women (LSM vs. C), haemoglobin levels among women (HI+LSM vs. C), haemoglobin levels among children 6–23 months (HI vs. C), haemoglobin levels among children 6–23 months (LSM vs. C), haemoglobin levels among children 6–23 months (HI+LSM vs. C), haemoglobin levels among children 6–59 months (HI vs. C), haemoglobin levels among children 6–59 months (LSM vs. C), haemoglobin levels among children 6–59 months (HI+LSM vs. C)  **Environment**  **Positive:** indoor A.arabiensis mosquito density (LSM vs. C), indoor A.arabiensis mosquito density (HI+LSM vs. C)  **Negative:** outdoor A.arabiensis mosquito density (HI+LSM vs. C)  **Null:** entomological inoculation rate (HI vs. C)*, entomological inoculation rate (LSM vs. C)*, entomological inoculation rate (LSM+HI vs. C)*, indoor anopheles mosquito density (HI vs. C), indoor anopheles mosquito density (LSM vs. C), indoor anopheles mosquito density (HI+LSM vs. C), indoor A.arabiensis mosquito density (HI vs. C), indoor A.funestus mosquito density (HI vs. C) , indoor A.funestus mosquito density (LSM vs. C), indoor A.funestus mosquito density |
|  |  |  |  |  |  | (HI+LSM vs. C), outdoor anopheles mosquito density (HI vs. C), outdoor anopheles mosquito density (LSM vs. C), outdoor anopheles mosquito density (HI+LSM vs. C), outdoor A.arabiensis mosquito density (HI vs. C), outdoor A.arabiensis mosquito density (LSM vs. C), outdoor A.funestus mosquito density (HI vs. C), outdoor A.funestus mosquito density (LSM vs. C), outdoor A.funestus mosquito density (HI+LSM vs. C), anopheles larval density (LSM vs. non-LSM) |
| **Neglected tropical diseases** | |  |  |  |  |  |
| Andersson (2015) [21, 37-42] | CRT of census enumeration areas | Mexico, Nicaragua | Community-led dengue control | SOC | Household,  *N* = 18,838 | **Mortality and morbidity**  **Negative:** self-reported dengue infection*, dengue infection based on serology*  **Behaviour**  **Positive:** agree bathing in water with temephos is harmful  **Negative:** agree with pesticide use, agree temephos and fumigation is the best way to control mosquitos, purchased pesticide, temephos present in at least one water container, households that purchased anti-mosquito products, households that spent >USD 3.25 on anti-mosquito products  **Null:** recognise larvae and know its relevance, intention to eliminate breeding sites, agree drinking or cooking with water with temephos is harmful, temephos placed in water, temephos removed after 1 month or no temephos  **Community and social**  **Positive:** agree communities can control dengue  **Null:** neighbours agree it is worthwhile to eliminate breeding site, discuss with neighbours about dengue control, agree neighbours help one another, social capital  **Environment**  **Positive:** absence of larvae or pupae in households, absence of pupae in households  **Negative:** households with larvae or pupae*, containers with larvae or purpae*, containers with larvae or pupae (among households)*, pupae per person*, larvae or pupae among households with regular water supply, larvae or pupae among |
|  |  |  |  |  |  | households with irregular water supply, pupae among households with regular water supply, pupae among households with irregular water supply, pupal productivity in rainy season, pupal productivity in dry season, pupae per household in rainy season, pupae per household in dry season, pupae per person in rainy season, pupae per person in dry season, households with larvae or pupae in rainy season, households with larvae or pupae in dry season, containers with larvae or pupae in rainy season, containers with larvae or pupae in dry season  **Other**  **Null:** visits by temephos government programme, work/school days lost by the dengue patients, work/school days lost by caregivers of dengue patients |
| Massa (2009) [22, 43] | CRT of school catchment areas | Tanzania | Community-directed distribution of treatment for schistosomiasis and soil-transmitted helminthiasis | School-based treatment for schistosomiasis and soil-transmitted helminthiasis | Individual, 6–15 years, *N* = 1,143 | **Mortality and morbidity**  **Negative:** S. haematobium prevalence*, ascaris lumbricoides prevalence*, hookworm prevalence*, ascaris lumbricoides intensity, hookworm intensity  **Null:** S. mansoni prevalence*, trichuris trichiura prevalence*, S. mansoni intensity, S. haematobium intensity, trichuris trichiura intensity  **Health care access and utilisation**  **Positive:** treatment coverage among non-enrolled children at first round  **Null:** treatment coverage among enrolled children at first round |
| **Multiple diseases** | |  |  |  |  |  |
| Lewycka (2013) [23] | Factorial CRT of census enumeration areas | Malawi | Participatory women’s groups for maternal and child health  Participatory women’s groups and peer counselling | Peer counselling  Enhanced SOC | Individual, pregnant women,  *N* = 3,033 | **Mortality and morbidity**^†^  **Null:** maternal mortality rate*, perinatal mortality rate*, neonatal mortality rate*, infant mortality rate*, any perceived antenatal, delivery, or postnatal maternal problem, any perceived infant problem (cough, fever, or diarrhoea)  **Health care access and utilisation**^†^  **Positive:** any antenatal care at a health facility, infant received BCG, OPV3, and DTP3 by 6 months  **Negative:** birth attended by a traditional birth attendant  **Null:** four or more antenatal care visits, any iron and folate, iron |
|  |  |  |  |  |  | or folate given for more than 90 days, any tetanus toxoid immunisation, adequate tetanus toxoid immunisation, any sulfadoxine-pyrimethamine, two or more doses of sulfadoxine-pyrimethamine, any HIV testing at antenatal care, institutional delivery, birth attended by skilled provider, attendant washed hands or wore gloves, infant wrapped within 30 minutes, infant bathed after 24 hours, postnatal care at a health facility,  **Behaviour**^†^  **Positive:** infant exclusively breastfed to 6 months  **Null:** bed net used every night during pregnancy, breastfeeding initiated within 1 hour of birth, use of prelacteal feeds, any breastfeeding problem |
| Makaula (2019) [24] | CRT of health facility catchment areas | Malawi | Community-directed primary health care | SOC | Household,  *N* = 4,511 | **Health care access and utilisation**  **Null:** antimalarial drug use, vitamin A use, praziquantel use  **Behaviour**  **Positive:** long-lasting insecticide treated net use among pregnant women, long-lasting insecticide treated net use among children  **Null:** long-lasting insecticide treated bed net use among households |
| Nair (2017) [25] | CRT of villages and adjoining hamlets | India | Participatory women’s groups for maternal and child health | Enhanced SOC | Individual, pregnant women, *N* = 3,001 | **Mortality and morbidity**  **Positive:** length-for-age z-score*  **Negative:** underweight at 18 months  **Null:** maternal mid-upper arm circumference in third trimester of pregnancy, maternal body mass index at 9 months postpartum, birthweight, change in length-for-age from birth to 18 months, weight-for-height at 18 months, weight-for-age at 18 months, mid-upper arm circumference at 18 months, stunting at 18 months, wasting at 18 months, infant diarrhoea, cough, fever in past 2 weeks, infant mortality  **Health care access and utilisation**  **Null:** infant received appropriate home care during illness episode, care sought for infant from a nurse or doctor, infant received BCG, OPV3, DTP3, measles, hepatitis B vaccine  **Behaviour** |
|  |  |  |  |  |  | **Positive:** minimum dietary diversity, infant with minimum dietary diversity, infant given minimum meal frequency, infant hand washed before feeding, infant hand washed after helping with defecation, infant hand washed after defecation  **Null:** ate more than three times in last day, infant exclusively breastfed until 6 months, infant started complementary foods at 6 months |

C, control; CLTS, community-led total sanitation; CRT, cluster randomised trial; HI, house improvement; HIVST, HIV self-testing; HW, handwashing; IPV, intimate partner violence; LSM, larval source management; NGO; non-governmental organisation; SOC, standard of care; USD, US dollars.

* Primary outcomes.

^†^ Comparison of participatory women’s groups (alone and combined with peer counselling) versus the SOC (alone and combined with peer counselling)

## Table E. Results from costing studies

| **Article** | **Study design** | **Setting** | **Intervention** | **Perspective** | **Economic or financial** | **Full or incremental** | **Cost scope** | **Unit cost estimates** |
| --- | --- | --- | --- | --- | --- | --- | --- | --- |
| **Diarrhoeal diseases** | |  |  |  |  |  |  |  |
| Briceño (2017) [11, 26] | Micro-costing | Tanzania | CLTS | Societal | Economic | Full | Above service delivery  Service delivery  Community | Cost of sanitation per person reached, $7.01 (*N* = NR)  Cost of sanitation and handwashing per person reached, $11.69 (*N* = NR) |
| Cha (2021) [13, 27] | Gross and micro-costing | Ethiopia | CLTS | Societal | Economic | Full | Above service delivery  Service delivery  Community | NR |
| Crocker (2016) [14, 44, 45] | Micro-costing | Ghana | CLTS with training of natural leaders | Societal | Economic | Full | Above service delivery  Service delivery  Community | Cost of sanitation with natural leaders per household*, $103.92 (*N* = NR)  Cost of sanitation per household*, $38.28 (*N* = NR) |
| **HIV** |  |  |  |  |  |  |  |  |
| Abramsky (2014) [17, 46] | Gross and micro-costing | Uganda | Community mobilisation for HIV and IPV prevention | Provider | Economic | Full | Above service delivery  Service delivery  Community | Cost per person reached, $27  (*N* = 10,333)  Cost per activist supported, $1,996 (*N* = 351)  Cost per activity, $59  (*N* = 11,877) |
| Indravudh (2021) [18, 28] | Gross and micro-costing | Malawi | Community-led HIVST | Provider | Economic | Full | Above service delivery  Service delivery  Community | Cost per HIV self-test distributed, $6.42 (*N* = 24,316)  Cost per person HIV self-tested, $6.45 (*N* = 24,219) |
| Sibanda (2021) [19] | Gross and micro-costing | Zimbabwe | Community-led HIVST | Provider | Economic | Full | Above service delivery  Service delivery  Community | Cost per HIV self-test distributed, $11.11 (*N* = 27,812) |
| **Malaria** |  |  |  |  |  |  |  |  |
| McCann (2021) [20, 47] | Gross and micro-costing | Malawi | Community-driven larval source management and house improvement | Societal | Economic | Full | Above service delivery  Service delivery  Community | Annual cost of larval source management per household, $129.05 (*N* = 1,520)  Annual cost of larval source management per person, $28.85 (*N* = 6,801)  Annual cost of house improvement per household, $138.05 (*N* = 1,030)  Annual cost of house improvement per person, $31.13 (*N* = 4,568) |
| **Neglected tropical diseases** | |  |  |  |  |  |  |  |
| Andersson (2015) [21, 29] | Micro-costing | Mexico, Nicaragua | Community-led dengue control | Provider | Economic | Full | Above service delivery  Service delivery | Annual cost per capita in Mexico, $20.78 (*N* = NR)  Annual cost per capita in Nicaragua, $9.29 (*N* = NR) |
| **Multiple diseases** | |  |  |  |  |  |  |  |
| Lewycka (2013) [23] | Gross and micro-costing | Malawi | Participatory women’s groups for maternal and child health | Provider | Economic | Full | Above service delivery  Service delivery  Community | Cost per year per woman of childbearing age, $7.09 (*N* = NR)  Cost per year per infant, $21.03  (*N* = NR) |
| Makaula (2019) [24] | Micro-costing | Malawi | Community-directed primary health care | Provider | Economic | Full | Service delivery  Community | NR |
| Nair (2017) [25] | Gross and micro-costing | India | Participatory women’s groups for maternal and child health | Provider | Economic | Full | Above service delivery  Service delivery | Annual cost per livebirth, $340  (*N* = NR)  Annual cost per pregnant woman, 18 (*N* = NR) |

CLTS, community-led total sanitation; HIVST, HIV self-testing; IPV, intimate partner violence; NR, not reported. Costs are reported in 2023 US dollars.

* Costs not reported in 2023 US dollars due to unknown currency year.

## Table F. Results from economic evaluations

| **Article** | **Study design** | **Setting** | **Intervention** | **Control** | **Perspective** | **Time horizon** | **CE estimate** | **CE probability** |
| --- | --- | --- | --- | --- | --- | --- | --- | --- |
| **Diarrhoeal diseases** | |  |  |  |  |  |  |  |
| Briceño (2017) [11, 26] | Trial-based | Tanzania | CLTS  CLTS and handwashing promotion | Handwashing promotion  No intervention | Societal | 4 years | Incremental cost per household accessed improved latrine, SN vs C: $251 (95% CI $176–$436)  Incremental cost per household accessed improved latrine, HW+SN vs C: $635 (95% CI $399–$1551)  Incremental cost per person accessed improved latrine, SN vs C: $46 (95% CI $31–$78)  Incremental cost per person accessed improved latrine, HW+SN vs C: $113 (95% CI $71–$277) | NR |
| Cha (2021) [13, 27] | Modelled | Ethiopia | CLTS | SOC | Societal | 10 years | Benefit-cost ratio, 4.6  Net present value, $1,346,629 | 100% |
| Crocker (2016) [14, 44, 45] | Trial-based | Ghana | CLTS with training of natural leaders | CLTS | Societal | 2.5 years | Incremental cost per household stopped open defecation*, $505  Incremental cost per household accessed improved latrine*, $1,205 | NR |
| **HIV** |  |  |  |  |  |  |  |  |
| Abramsky (2014) [17, 46] | Trial-based | Uganda | Community mobilisation for HIV and IPV prevention | Enhanced SOC | Provider | 1 year | Incremental cost per physical IPV case averted, $582 | NR |
| Indravudh (2021) [18, 28] | Trial-based | Malawi | Community-led HIV self-testing | SOC | Provider | 1 year | Incremental cost per additional person tested HIV positive, $365 | 45% |
| **Neglected tropical diseases** | |  |  |  |  |  |  |  |
| Andersson (2015) [21, 29] | Trial-based | Mexico, Nicaragua | Community-led dengue control | SOC | Provider | 1 year | Incremental cost per DALY averted in Mexico, $36,809 (95% CI $17,236–$83,139)  Incremental cost per DALY averted in Nicaragua, $36,284 (95% CI $17,764–$89,704) | Mexico, 51%  Nicaragua, 0% |
| **Multiple diseases** | |  |  |  |  |  |  |  |
| Lewycka (2013) [23] | Trial-based | Malawi | Participatory women’s groups for maternal and child health  Participatory women’s groups and peer counselling | Peer counselling  Enhanced SOC | Provider | 4.5 years | Incremental cost per life-year lost averted^†^, $148 | NR |
| Nair (2017) [25] | Trial-based | India | Participatory women’s groups for maternal and child health | Enhanced SOC | Provider | NR | Incremental cost per infant death averted, $34,680  Incremental cost per life-year saved, $1,125 | NR |

C, control; CE, cost-effectiveness; CLTS, community-led total sanitation; DALY, disability-adjusted life years, HW, handwashing, ICER, incremental cost-effectiveness ratio; NR, not reported; SOC, standard of care. Costs are reported in 2023 US dollars.

* Costs not reported in 2022 US dollars due to unknown currency year.

^†^ Comparison of participatory women’s groups (alone and combined with peer counselling) versus the SOC (alone and combined with peer counselling).

## Table G. Results from process evaluations

| **Article** | **Study design** | **Setting** | **Intervention** | **Implementation** | **Mechanisms of impact** | **Context** |
| --- | --- | --- | --- | --- | --- | --- |
| **Diarrhoeal diseases** | |  |  |  |  |  |
| Biran (2018) [10] | IDIs | Malawi | CLTS inclusive of people with disabilities | External actors and community health workers inconsistently conveyed messages on inclusive sanitation and promotion of constructing or adapting latrines. Activities did not achieve full participation from people with disabilities, including attendance at community participation due to accessibility of meetings and representation on sanitation committees. Barriers to change included the perceived cost and physical ability to construct or adapt a latrine. | Community members were more likely to change sanitation behaviours if they were exposed to more activities. | NR |
| Cameron (2019) [12, 48] | Panel surveys | Indonesia | CLTS | Intervention villages exposed to resource agencies compared with government agencies were more likely to have greater engagement with community health officers, intensity of implementation, and participation by community members. | In the intervention arm, households in villages with higher levels of social capital were more likely to have constructed a toilet, potentially by imposing social sanctions. | The intervention effect on diarrhoeal prevalence varied by sex of the head of household, with further reductions among female household heads, and ethnic groups. |
| Crocker (2016) [14] | Cross-sectional surveys | Ghana | CLTS | Participation from community members was similar across arms, with one-third attending any WASH meetings and one-third discussing WASH topics with a neighbour. | The intensity of WASH activities, including time spent by natural leaders and community members, was higher in the intervention arm. | NR |
| Pickering (2015) [15] | Panel surveys | Mali | CLTS | Among households, 85% attended triggering events. | Among households, 95% were exposed to promotion of latrine building. Open defecation free | NR |
| Quattrochi (2018) [16, 31] | Panel surveys | Democratic Republic of Congo | Community-led WASH | NR | The intervention effect on water and sanitation outcomes persisted over time, but confidence in government and traditional leaders was not found to be a mechanism of impact. | NR |
| **HIV** |  |  |  |  |  |  |
| Abramsky (2014) [17, 33, 49-53] | Cross-sectional surveys, monitoring forms, IDIs | Uganda | Community mobilisation for HIV and IPV prevention | External actors supported over 400 community activists, who led more than 11,000 activities that reached 260,000 community members. Factors facilitating implementation and uptake included participatory and collective activities, proximity of activities, established trust between community activists and community members, and availability and support of community activists. | In quantitative analysis, the intervention achieved high coverage, with 91% of men and 68% of women reporting exposure to activities, communication materials or multi-media. A dose-response relationship was observed between intervention exposure and changes in interpersonal relationships and willingness to intervene to prevent IPV. Norms related to IPV were the most important mediators of the intervention effect on physical IPV at the community level, followed by norms related to gender roles and power dynamics. Trust and attitudes around violence mediated the intervention effect at the relationship and individual levels, respectively. In qualitative analysis, participatory community activities facilitated a strong sense of collective engagement and contributed to changing norms and perceptions around IPV. Community and | Men compared with women reported higher intervention exposure. Changes in outcomes also varied based on willingness to change gender norms and relationship dynamics and personal experience with IPV. |
|  |  |  |  |  | household conversations contributed to shifting gender norms and power dynamics, enhancing communication, and introducing skills for conflict resolution, which strengthened interpersonal relationships and reduced IPV and HIV risk.  Ongoing availability and support by community activists bolstered change by establishing trust, reinforcing messages, and providing accountability. Social networks facilitated diffusion of intervention messages and encouraged participation in activities. |  |
| Indravudh (2021) [18, 34] | Cross-sectional surveys, monitoring forms | Malawi | Community-led HIVST | External actors supported 157 community health group members and 190 community volunteers, who distributed 24,316 HIV self-tests. Implementation strategies involved sensitisation and distribution of HIV self-testing kits at village head-led community meetings, homes, and fixed locations and social hotspots. Strategies to support linkage to routine HIV services included active post-test follow-up, phone referrals to health facilities, and material assistance. | HIV self-testing awareness and uptake was 95% and 75%, respectively. In the intervention arm, social cohesion, community concern, and critical consciousness had a non-linear association with HIV testing. However, community measures were not found to be mediators of impact. | The intervention effect on HIV testing among adolescents was higher among younger age groups and boys. |
| Sibanda (2021) [19, 35] | Cross-sectional surveys, monitoring forms | Zimbabwe | Community-led HIVST | Community distributors provided 27,812 HIV self-tests. | HIV self-testing uptake was 21.6%. | The intervention effect on linkage to HIV care was higher among men. The intervention effect also varied by community measures. For example, the intervention effect on new HIV diagnosis was higher among village groups with high social cohesion. |
| **Malaria** |  |  |  |  |  |  |
| McCann (2021) [20, 36, 54-56] | Monitoring forms, IDIs, FGDs | Malawi | Community-driven larval source management and house improvement | Village committees and health animators valued knowledge and skills gained from trainings, which they were able to transfer to community members through group workshops. Health animators and village committee members conducted 172 group workshops, with factors facilitating attendance including the presence of community leaders and community health workers. Implementation factors facilitating uptake included observing and evaluating changes in malaria outcomes. Barriers included labour intensiveness, time requirements, and lack of financial incentives or material support. | Community members were aware of the role and work of village committees and health animators. Through group workshops, community members were aware of malaria as a health problem, its sources of transmission, and methods of control, which motivated actions to prevent malaria. Attendance at group workshops was varied. Motivation to manage malaria acted as a facilitator, while time availability acted as a barrier, with attendance lower among men and young people. Factors facilitating individual behaviour change included repeat attendance at workshops. Engagement with village committees and health animators also facilitated attitudes and practices for malaria prevention, especially for novel strategies such as larval source management. | Barriers included lack of trust in larvicides. |
| **Neglected tropical diseases** | |  |  |  |  |  |
| Andersson (2015) [21, 37] | Cross-sectional surveys | Mexico, Nicaragua | Community-led dengue control | NR | In the intervention arm, households with higher social capital were more likely to have larvae and/or pupae absent. | NR |
| Massa (2009) [22, 57] | IDIs, FGDs | Tanzania | Community-directed distribution of treatment for schistosomiasis and soil-transmitted helminthiasis | Community leaders and members selected community drug distributors based on their reputation and level of education. Sensitisation was done at village meetings. Community distributors delivered drugs at homes and fixed locations. Factors facilitating implementation included trust between community drug distributors and community members. | Community members were aware of drug distribution and the importance of treatment. | NR |
| **Multiple diseases** | |  |  |  |  |  |
| Lewycka (2013) [23, 58] | Cross-sectional surveys, monitoring forms | Malawi | Participatory women’s groups for maternal and child health | Community facilitators established 207 women’s groups, with more than 12,000 people attending at least once. Among women, 59% attended 1–5 times, 30% attended 6–10 times, and 11% attended more than 10 times. Women's groups identified a breadth of strategies, which were implemented by groups alone or in collaboration with health care providers. Strategies included health education, bicycle ambulances, distribution of health commodities, mobile clinics, garden cultivation, and income generation. Resources were raised through advocacy, fundraising, or partnerships with stakeholders. | NR | NR |
| Makaula (2019) [24] | IDIs, FGDs | Malawi | Community-directed | Selection of qualified community volunteers and | NR | NR |
|  |  |  | primary health care | engagement with multi-level stakeholders facilitated implementation. |  |  |
| Nair (2017) [25] | Cross-sectional surveys, monitoring forms | India | Participatory women’s groups for maternal and child health | Community-based workers established 163 women's groups, with common strategies including a combination of home-based preventive actions, care-seeking, and community-level activities (e.g., kitchen gardens, campaigning). Among women, 56% attended meetings at least once and 80% received visits from community-based workers. | NR | NR |

CLTS, community-led total sanitation; FGD, focus group discussion, HIVST, HIV self-testing; IDI, in-depth interview, IPV, intimate partner violence; NR, not reported; WASH, water, sanitation, and hygiene.

## Figure A. Radar graph of community participation scores

Radar graph illustrating the level of community participation for stages of design, implementation, monitoring and evaluation, and post-implementation. Each line represents a single study, with each stage of the intervention for that study scored from 0 to 4 (0=no information, 1=information giving, 2=consultation, 3=collaboration, 4=empowerment). Points closer to the centre indicate lower scores of community participation, while points further from the centre indicates higher scores of community participation. M&E, monitoring and evaluation.

**References**

1. Arnstein SR. A ladder Of citizen participation. JAPA. 1969;35(4):216-24. doi: 10.1080/01944366908977225.

2. Labonte R. Health Promotion and Empowerment: Practice Frameworks. Toronto: Centre for Health Promotion, University of Toronto, 1993.

3. Laverack G, Labonte R. A planning framework for community empowerment goals within health promotion. Health Policy Plan. 2000;15(3):255-62. doi: 10.1093/heapol/15.3.255. PubMed PMID: 11012399.

4. McLeroy KR, Norton BL, Kegler MC, Burdine JN, Sumaya CV. Community-based interventions. Am J Public Health. 2003;93(4):529-33. doi: 10.2105/ajph.93.4.529. PubMed PMID: 12660190; PubMed Central PMCID: PMCPMC1447783.

5. Rothman J, Erlich J, Tropman JE. Strategies of Community Intervention. 1st ed. Itasca: F.E. Peacock Publishers; 2001.

6. Rifkin SB, Pridmore P. Partners in Planning: Information, Participation and Empowerment. 1st ed. London: Macmillan Education Ltd; 2001.

7. Draper AK, Hewitt G, Rifkin S. Chasing the dragon: developing indicators for the assessment of community participation in health programmes. Soc Sci Med. 2010;71(6):1102-9. Epub 20100619. doi: 10.1016/j.socscimed.2010.05.016. PubMed PMID: 20621405.

8. Higgins J, Thomas J, Chandler J, Cumpston M, Li T, Page M, et al. Cochrane handbook for systematic reviews of interventions 2022 [updated February 2022]. Available from: <www.training.cochrane.org/handbook>.

9. Drummond MF, Jefferson TO. Guidelines for authors and peer reviewers of economic submissions to the BMJ. BMJ. 1996;313(7052):275-83. doi: 10.1136/bmj.313.7052.275. PubMed PMID: 8704542; PubMed Central PMCID: PMCPMC2351717.

10. Biran A, Danquah L, Chunga J, Schmidt WP, Holm R, Itimu-Phiri A, et al. A cluster-randomised trial to evaluate the impact of an inclusive, community-led total sanitation intervention on sanitation access for people with disabilities in Malawi. Am J Trop Med Hyg. 2018;98(4):984-94. Epub 20180201. doi: 10.4269/ajtmh.17-0435. PubMed PMID: 29405106; PubMed Central PMCID: PMCPMC5928815.

11. Briceño B, Coville A, Gertler P, Martinez S. Are there synergies from combining hygiene and sanitation promotion campaigns: evidence from a large-scale cluster-randomised trial in rural Tanzania. PLOS One. 2017;12(11):e0186228. Epub 20171101. doi: 10.1371/journal.pone.0186228. PubMed PMID: 29091726; PubMed Central PMCID: PMCPMC5665426.

12. Cameron L, Olivia S, Shah M. Scaling up sanitation: Evidence from an RCT in Indonesia. J Dev Econ. 2019;138:1-16. Epub 2019/05/07. doi: 10.1016/j.jdeveco.2018.12.001. PubMed PMID: 31057208; PubMed Central PMCID: PMCPMC6472610.

13. Cha S, Jung S, Bizuneh DB, Abera T, Doh YA, Seong J, et al. Effect of a community-led total sanitation intervention on the incidence and prevalence of diarrhoea in children in rural Ethiopia: a cluster-randomised controlled trial. Am J Trop Med Hyg. 2021;105(2):532-43. Epub 20210624. doi: 10.4269/ajtmh.20-0014. PubMed PMID: 34125700; PubMed Central PMCID: PMCPMC8437198.

14. Crocker J, Abodoo E, Asamani D, Domapielle W, Gyapong B, Bartram J. Impact evaluation of training natural leaders during a community-led total sanitation intervention: a cluster-randomised field trial in Ghana. Environ Sci Technol. 2016;50(16):8867-75. Epub 20160729. doi: 10.1021/acs.est.6b01557. PubMed PMID: 27428399; PubMed Central PMCID: PMCPMC4989246.

15. Pickering AJ, Djebbari H, Lopez C, Coulibaly M, Alzua ML. Effect of a community-led sanitation intervention on child diarrhoea and child growth in rural Mali: a cluster-randomised controlled trial. Lancet Glob Health. 2015;3(11):e701-11. Epub 2015/10/18. doi: 10.1016/S2214-109X(15)00144-8. PubMed PMID: 26475017.

16. Quattrochi JP, Coville A, Mvukiyehe E, Dohou CJ, Esu F, Cohen B, et al. Effects of a community-driven water, sanitation and hygiene intervention on water and sanitation infrastructure, access, behaviour, and governance: a cluster-randomised controlled trial in rural Democratic Republic of Congo. BMJ Glob Health. 2021;6(5):e005030. Epub 2021/05/19. doi: 10.1136/bmjgh-2021-005030. PubMed PMID: 34001519; PubMed Central PMCID: PMCPMC8130731.

17. Abramsky T, Devries K, Kiss L, Nakuti J, Kyegombe N, Starmann E, et al. Findings from the SASA! Study: a cluster randomised controlled trial to assess the impact of a community mobilisation intervention to prevent violence against women and reduce HIV risk in Kampala, Uganda. BMC Med. 2014;12:122. Epub 20140731. doi: 10.1186/s12916-014-0122-5. PubMed PMID: 25248996; PubMed Central PMCID: PMCPMC4243194.

18. Indravudh PP, Fielding K, Kumwenda MK, Nzawa R, Chilongosi R, Desmond N, et al. Effect of community-led delivery of HIV self-testing on HIV testing and antiretroviral therapy initiation in Malawi: a cluster-randomised trial. PLOS Med. 2021;18(5):e1003608. Epub 20210511. doi: 10.1371/journal.pmed.1003608. PubMed PMID: 33974621; PubMed Central PMCID: PMCPMC8112698.

19. Sibanda EL, Mangenah C, Neuman M, Tumushime M, Watadzaushe C, Mutseta MN, et al. Comparison of community-led distribution of HIV self-tests kits with distribution by paid distributors: a cluster randomised trial in rural Zimbabwean communities. BMJ Glob Health. 2021;6(Suppl 4). doi: 10.1136/bmjgh-2021-005000. PubMed PMID: 34275872; PubMed Central PMCID: PMCPMC8287604.

20. McCann RS, Kabaghe AN, Moraga P, Gowelo S, Mburu MM, Tizifa T, et al. The effect of community-driven larval source management and house improvement on malaria transmission when added to the standard malaria control strategies in Malawi: a cluster-randomised controlled trial. Malar J. 2021;20(1):232. Epub 20210522. doi: 10.1186/s12936-021-03769-0. PubMed PMID: 34022912; PubMed Central PMCID: PMCPMC8140568.

21. Andersson N, Nava-Aguilera E, Arostegui J, Morales-Perez A, Suazo-Laguna H, Legorreta-Soberanis J, et al. Evidence based community mobilisation for dengue prevention in Nicaragua and Mexico (Camino Verde, the Green Way): cluster randomised controlled trial. BMJ. 2015;351:h3267. Epub 20150708. doi: 10.1136/bmj.h3267. PubMed PMID: 26156323; PubMed Central PMCID: PMCPMC4495677.

22. Massa K, Magnussen P, Sheshe A, Ntakamulenga R, Ndawi B, Olsen A. The effect of the community-directed treatment approach versus the school-based treatment approach on the prevalence and intensity of schistosomiasis and soil-transmitted helminthiasis among schoolchildren in Tanzania. Trans R Soc Trop Med Hyg. 2009;103(1):31-7. Epub 20080903. doi: 10.1016/j.trstmh.2008.07.009. PubMed PMID: 18771789.

23. Lewycka S, Mwansambo C, Rosato M, Kazembe P, Phiri T, Mganga A, et al. Effect of women's groups and volunteer peer counselling on rates of mortality, morbidity, and health behaviours in mothers and children in rural Malawi (MaiMwana): a factorial, cluster-randomised controlled trial. Lancet. 2013;381(9879):1721-35. Epub 2013/05/21. doi: 10.1016/S0140-6736(12)61959-X. PubMed PMID: 23683639; PubMed Central PMCID: PMCPMC3796349.

24. Makaula P, Funsanani M, Mamba KC, Musaya J, Bloch P. Strengthening primary health care at district-level in Malawi - determining the coverage, costs, and benefits of community-directed interventions. BMC Health Serv Res. 2019;19(1):509. Epub 20190722. doi: 10.1186/s12913-019-4341-5. PubMed PMID: 31331346; PubMed Central PMCID: PMCPMC6647329.

25. Nair N, Tripathy P, Sachdev HS, Pradhan H, Bhattacharyya S, Gope R, et al. Effect of participatory women's groups and counselling through home visits on children's linear growth in rural eastern India (CARING trial): a cluster-randomised controlled trial. Lancet Glob Health. 2017;5(10):e1004-e16. doi: 10.1016/S2214-109X(17)30339-X. PubMed PMID: 28911749; PubMed Central PMCID: PMCPMC5640793.

26. Briceño B, Chase C. Cost-efficiency of rural sanitation promotion: activity-based costing and experimental evidence from Tanzania. J Dev Effect. 2015;7(4):1-12. doi: 10.1080/19439342.2015.1105848.

27. Cha S, Jung S, Bizuneh DB, Abera T, Doh YA, Seong J, et al. Benefits and costs of a community-led total sanitation intervention in rural Ethiopia: a trial-based ex post economic evaluation. Int J Environ Res Public Health. 2020;17(14). Epub 20200714. doi: 10.3390/ijerph17145068. PubMed PMID: 32674392; PubMed Central PMCID: PMCPMC7399893.

28. Indravudh PP, Fielding K, Sande LA, Maheswaran H, Mphande S, Kumwenda MK, et al. Pragmatic economic evaluation of community-led delivery of HIV self-testing in Malawi. BMJ Glob Health. 2021;6(Suppl 4):e004593. Epub 2021/07/20. doi: 10.1136/bmjgh-2020-004593. PubMed PMID: 34275869; PubMed Central PMCID: PMCPMC8287609.

29. Tschampl CA, Undurraga EA, Ledogar RJ, Coloma J, Legorreta-Soberanis J, Paredes-Solis S, et al. Cost-effectiveness of community mobilisation (Camino Verde) for dengue prevention in Nicaragua and Mexico: a cluster randomised controlled trial. Int J Infect Dis. 2020;94:59-67. Epub 20200314. doi: 10.1016/j.ijid.2020.03.026. PubMed PMID: 32179138.

30. Crocker J, Saywell D, Bartram J. Sustainability of community-led total sanitation outcomes: Evidence from Ethiopia and Ghana. Int J Hyg Environ Health. 2017;220(3):551-7. Epub 20170509. doi: 10.1016/j.ijheh.2017.02.011. PubMed PMID: 28522255; PubMed Central PMCID: PMCPMC5475437.

31. Croke K, Coville A, Mvukiyehe E, Dohou CJ, Zibika JP, Stanus Ghib L, et al. Effects of a community-driven water, sanitation, and hygiene programme on COVID-19 symptoms, vaccine acceptance and non-COVID illnesses: a cluster-randomised controlled trial in rural Democratic Republic of Congo. Trop Med Int Health. 2022;27(9):795-802. Epub 20220814. doi: 10.1111/tmi.13799. PubMed PMID: 35832019; PubMed Central PMCID: PMCPMC9349788.

32. Abramsky T, Devries KM, Michau L, Nakuti J, Musuya T, Kyegombe N, et al. The impact of SASA!, a community mobilisation intervention, on women's experiences of intimate partner violence: secondary findings from a cluster randomised trial in Kampala, Uganda. J Epidemiol Community Health. 2016;70(8):818-25. Epub 2016/02/14. doi: 10.1136/jech-2015-206665. PubMed PMID: 26873948; PubMed Central PMCID: PMCPMC4975800.

33. Kyegombe N, Abramsky T, Devries KM, Starmann E, Michau L, Nakuti J, et al. The impact of SASA!, a community mobilisation intervention, on reported HIV-related risk behaviours and relationship dynamics in Kampala, Uganda. J Int AIDS Soc. 2014;17(1):19232. Epub 20141105. doi: 10.7448/IAS.17.1.19232

19232. PubMed PMID: 25377588; PubMed Central PMCID: PMCPMC4223282.

34. Indravudh PP, Terris-Prestholt F, Neuman M, Kumwenda MK, Chilongosi R, Johnson CC, et al. Understanding mechanisms of impact from community-led delivery of HIV self-testing: mediation analysis of a cluster-randomised trial in Malawi. PLOS Glob Public Health. 2022;2(10):e0001129. Epub 20221027. doi: 10.1371/journal.pgph.0001129. PubMed PMID: 36962622; PubMed Central PMCID: PMCPMC10021599.

35. Thomas KA, Sibanda EL, Johnson C, Watadzaushe C, Ncube G, Hatzold K, et al. Do community measures impact the effectiveness of a community led HIV testing intervention. Secondary analysis of an HIV self-testing intervention in rural communities in Zimbabwe. BMC Infect Dis. 2023;22(Suppl 1):974. Epub 20231031. doi: 10.1186/s12879-023-08695-x. PubMed PMID: 37907871; PubMed Central PMCID: PMCPMC10617038.

36. Gowelo S, Meijer P, Tizifa T, Malenga T, Mburu MM, Kabaghe AN, et al. Community participation in habitat management and larviciding for the control of malaria vectors in southern Malawi. Am J Trop Med Hyg. 2023;108(1):51-60. Epub 20221121. doi: 10.4269/ajtmh.21-1127. PubMed PMID: 36410320; PubMed Central PMCID: PMCPMC9833073.

37. Alvarado-Castro V, Paredes-Solis S, Nava-Aguilera E, Morales-Perez A, Flores-Moreno M, Legorreta-Soberanis J, et al. Social capital is associated with lower mosquito vector indices: secondary analysis from a cluster randomised controlled trial of community mobilisation for dengue prevention in Mexico. Popul Health Metr. 2019;17(1):18. Epub 20191210. doi: 10.1186/s12963-019-0199-3. PubMed PMID: 31823786; PubMed Central PMCID: PMCPMC6902442.

38. Carcamo A, Arostegui J, Coloma J, Harris E, Ledogar RJ, Andersson N. Informed community mobilisation for dengue prevention in households with and without a regular water supply: secondary analysis from the Camino Verde trial in Nicaragua. BMC Public Health. 2017;17(Suppl 1):395. Epub 20170530. doi: 10.1186/s12889-017-4295-7. PubMed PMID: 28699544; PubMed Central PMCID: PMCPMC5506562.

39. Jimenez-Alejo A, Morales-Perez A, Nava-Aguilera E, Flores-Moreno M, Apreza-Aguilar S, Carranza-Alcaraz W, et al. Pupal productivity in rainy and dry seasons: findings from the impact survey of a randomised controlled trial of dengue prevention in Guerrero, Mexico. BMC Public Health. 2017;17(Suppl 1):428. Epub 20170530. doi: 10.1186/s12889-017-4294-8. PubMed PMID: 28699555; PubMed Central PMCID: PMCPMC5506597.

40. Legorreta-Soberanis J, Paredes-Solis S, Morales-Perez A, Nava-Aguilera E, de Los Santos FRS, Sanchez-Gervacio BM, et al. Coverage and beliefs about temephos application for control of dengue vectors and impact of a community-based prevention intervention: secondary analysis from the Camino Verde trial in Mexico. BMC Public Health. 2017;17(Suppl 1):426. Epub 20170530. doi: 10.1186/s12889-017-4297-5. PubMed PMID: 28699554; PubMed Central PMCID: PMCPMC5506576.

41. Legorreta-Soberanis J, Paredes-Solis S, Morales-Perez A, Nava-Aguilera E, Serrano-de Los Santos FR, Dimas-Garcia DL, et al. Household costs of dengue illness: secondary outcomes from a randomised controlled trial of dengue prevention in Guerrero state, Mexico. BMC Public Health. 2017;17(Suppl 1):411. Epub 20170530. doi: 10.1186/s12889-017-4304-x. PubMed PMID: 28699565; PubMed Central PMCID: PMCPMC5506602.

42. Legorreta-Soberanis J, Paredes-Solis S, Morales-Perez A, Nava-Aguilera E, Serrano-de Los Santos FR, Sanchez-Gervacio BM, et al. Household costs for personal protection against mosquitoes: secondary outcomes from a randomised controlled trial of dengue prevention in Guerrero state, Mexico. BMC Public Health. 2017;17(Suppl 1):399. Epub 20170530. doi: 10.1186/s12889-017-4303-y. PubMed PMID: 28699550; PubMed Central PMCID: PMCPMC5506592.

43. Massa K, Olsen A, Sheshe A, Ntakamulenga R, Ndawi B, Magnussen P. Can coverage of schistosomiasis and soil transmitted helminthiasis control programmes targeting school-aged children be improved? New approaches. Parasitology. 2009;136(13):1781-8. Epub 20090130. doi: 10.1017/s0031182008000474. PubMed PMID: 19178756.

44. Crocker J, Saywell D, Shields KF, Kolsky P, Bartram J. The true costs of participatory sanitation: Evidence from community-led total sanitation studies in Ghana and Ethiopia. Sci Total Environ. 2017;601-602:1075-83. Epub 20170609. doi: 10.1016/j.scitotenv.2017.05.279. PubMed PMID: 28599364; PubMed Central PMCID: PMCPMC5536257.

45. Crocker J, Fuente D, Bartram J. Cost-effectiveness of community-led total sanitation in Ethiopia and Ghana. Int J Hyg Environ Health. 2021;232:113682. Epub 20201224. doi: 10.1016/j.ijheh.2020.113682. PubMed PMID: 33360500; PubMed Central PMCID: PMCPMC7873587.

46. Michaels-Igbokwe C, Abramsky T, Devries K, Michau L, Musuya T, Watts C. Cost and cost-effectiveness analysis of a community mobilisation intervention to reduce intimate partner violence in Kampala, Uganda. BMC Public Health. 2016;16:196. Epub 2016/03/01. doi: 10.1186/s12889-016-2883-6. PubMed PMID: 26924488; PubMed Central PMCID: PMCPMC4770522.

47. Phiri MD, McCann RS, Kabaghe AN, van den Berg H, Malenga T, Gowelo S, et al. Cost of community-led larval source management and house improvement for malaria control: a cost analysis within a cluster-randomised trial in a rural district in Malawi. Malar J. 2021;20(1):268. Epub 20210613. doi: 10.1186/s12936-021-03800-4. PubMed PMID: 34120608; PubMed Central PMCID: PMCPMC8200285.

48. Borja-Vega C. The effects of the Total Sanitation and Sanitation Marketing programme on gender and ethnic groups in Indonesia. Waterlines. 2014;33:55-70.

49. Abramsky T, Devries KM, Michau L, Nakuti J, Musuya T, Kiss L, et al. Ecological pathways to prevention: How does the SASA! community mobilisation model work to prevent physical intimate partner violence against women? BMC Public Health. 2016;16(1):339. Epub 2016/04/17. doi: 10.1186/s12889-016-3018-9. PubMed PMID: 27084116; PubMed Central PMCID: PMCPMC4833941.

50. Abramsky T, Musuya T, Namy S, Watts C, Michau L. Changing the norms that drive intimate partner violence: findings from a cluster randomised trial on what predisposes bystanders to take action in Kampala, Uganda. BMJ Glob Health. 2018;3(6):e001109. Epub 20181214. doi: 10.1136/bmjgh-2018-001109. PubMed PMID: 30613427; PubMed Central PMCID: PMCPMC6304103.

51. Kyegombe N, Starmann E, Devries KM, Michau L, Nakuti J, Musuya T, et al. “SASA! is the medicine that treats violence”. Qualitative findings on how a community mobilisation intervention to prevent violence against women created change in Kampala, Uganda. Glob Health Action. 2014;7:25082. Epub 20140912. doi: 10.3402/gha.v7.25082. PubMed PMID: 25226421; PubMed Central PMCID: PMCPMC4165071.

52. Starmann E, Collumbien M, Kyegombe N, Devries K, Michau L, Musuya T, et al. Exploring Couples' Processes of Change in the Context of SASA!, a Violence Against Women and HIV Prevention Intervention in Uganda. Prev Sci. 2017;18(2):233-44. doi: 10.1007/s11121-016-0716-6. PubMed PMID: 27682273; PubMed Central PMCID: PMCPMC5243896.

53. Starmann E, Heise L, Kyegombe N, Devries K, Abramsky T, Michau L, et al. Examining diffusion to understand the how of SASA!, a violence against women and HIV prevention intervention in Uganda. BMC Public Health. 2018;18(1):616. Epub 20180511. doi: 10.1186/s12889-018-5508-4. PubMed PMID: 29751754; PubMed Central PMCID: PMCPMC5948738.

54. Gowelo S, McCann RS, Koenraadt CJM, Takken W, van den Berg H, Manda-Taylor L. Community factors affecting participation in larval source management for malaria control in Chikwawa district, southern Malawi. Malar J. 2020;19(1):195. Epub 20200602. doi: 10.1186/s12936-020-03268-8. PubMed PMID: 32487233; PubMed Central PMCID: PMCPMC7265157.

55. Kaunda-Khangamwa BN, van den Berg H, McCann RS, Kabaghe A, Takken W, Phiri K, et al. The role of health animators in malaria control: a qualitative study of the health animator approach within the Majete malaria project in Chikwawa District, Malawi. BMC Health Serv Res. 2019;19(1):478. Epub 20190712. doi: 10.1186/s12913-019-4320-x. PubMed PMID: 31299974; PubMed Central PMCID: PMCPMC6624973.

56. Malenga T, Kabaghe AN, Manda-Taylor L, Kadama A, McCann RS, Phiri KS, et al. Malaria control in rural Malawi: implementing peer health education for behaviour change. Global Health. 2017;13(1):84. Epub 20171120. doi: 10.1186/s12992-017-0309-6. PubMed PMID: 29157284; PubMed Central PMCID: PMCPMC5694909.

57. Massa K, Magnussen P, Sheshe A, Ntakamulenga R, Ndawi B, Olsen A. Community perceptions on the community-directed treatment and school-based approaches for the control of schistosomiasis and soil-transmitted helminthiasis among school-age children in Lushoto District, Tanzania. J Biosoc Sci. 2009;41(1):89-105. Epub 20080723. doi: 10.1017/s0021932008002964. PubMed PMID: 18647439.

58. Rosato M, Malamba F, Kunyenge B, Phiri T, Mwansambo C, Kazembe P, et al. Strategies developed and implemented by women's groups to improve mother and infant health and reduce mortality in rural Malawi. Int Health. 2012;4(3):176-84. doi: 10.1016/j.inhe.2012.03.007. PubMed PMID: 24029397.
