# Supplementary material for: Community-led strategies for communicable disease prevention and management in low- and middle- income countries: A mixed-methods systematic review of health, social, and economic impact
Source: PLOS Glob Public Health. 2025 Apr 2;5(4):e0004304. doi: 10.1371/journal.pgph.0004304 (PMC11964228; doi:10.1371/journal.pgph.0004304)
Supplement: S1 Table — List of records reviewed at full text with reasons for inclusion and exclusion. (DOCX) [file pgph.0004304.s003.docx]

**S1 Table. List of records reviewed at full text**

| **Author** | **Year** | **Journal** | **Title** | **Eligible** | **Reason** | **Reason detailed** |
| --- | --- | --- | --- | --- | --- | --- |
| Abdulahi et al. | 2021 | Nutrients | Breastfeeding education and support to improve early initiation and exclusive breastfeeding practices and infant growth: a cluster randomised controlled trial from a rural Ethiopian setting | No | No CD outcome reported |  |
| Abeyewickreme et al. | 2012 | Pathog Glob Health | Community mobilisation and household level waste management for dengue vector control in Gampaha district of Sri Lanka: an intervention study | No | Community collaboration | Community volunteers organised activities and acted as liaisons between community members and external actors. |
| Abramsky et al. | 2014 | BMC Med | Findings from the SASA! study: a cluster randomised controlled trial to assess the impact of a community mobilisation intervention to prevent violence against women and reduce HIV risk in Kampala, Uganda | Yes | Community empowerment |  |
| Abramsky et al. | 2016 | BMC Public Health | Ecological pathways to prevention: how does the SASA! community mobilisation model work to prevent physical intimate partner violence against women? | Yes | Community empowerment |  |
| Abramsky et al. | 2016 | J Epidemiol Community Health | The impact of SASA!, a community mobilisation intervention, on women's experiences of intimate partner violence: secondary findings from a cluster randomised trial in Kampala, Uganda | Yes | Community empowerment |  |
| Abramsky et al. | 2018 | BMJ Glob Health | Changing the norms that drive intimate partner violence: findings from a cluster randomised trial on what predisposes bystanders to take action in Kampala, Uganda | Yes | Community empowerment |  |
| Accrombessi et al. | 2023 | Lancet | Efficacy of pyriproxyfen-pyrethroid long-lasting insecticidal nets (LLINs) and chlorfenapyr-pyrethroid LLINs compared with pyrethroid-only LLINs for malaria control in Benin: a cluster-randomised, superiority trial | No | Community collaboration | Community leaders were involved in sensitisation campaigns. |
| Adam et al. | 2021 | PLOS Med | Evaluation of a community-based mobile video breastfeeding intervention in Khayelitsha, South Africa: the Philani MOVIE cluster-randomised controlled trial | No | No CD outcome reported |  |
| Ahmed et al. | 1993 | Soc Sci Med | A longitudinal study of the impact of behavioural change intervention on cleanliness, diarrhoeal morbidity and growth of children in rural Bangladesh | No | Not cluster-randomised |  |
| Aiello et al. | 2012 | PLOS One | Facemasks, hand hygiene, and influenza among young adults: a randomised intervention trial | No | Community information-giving or consultation |  |
| Aikins et al. | 1998 | Soc Sci Med | The Gambian national impregnated bednet programme: costs, consequences and net cost-effectiveness | No | Not cluster-randomised |  |
| Alegbeleye et al. | 2019 | J Glob Health | Community engagement and mobilisation of local resources to support integrated community case management of childhood illnesses in Niger State, Nigeria | No | Not cluster-randomised |  |
| Alfonso et al. | 2016 | J Acquir Immune Defic Syndr | Trends in the marginal cost of male circumcision in rural Rakai, Uganda | No | Not cluster-randomised |  |
| Alhassan et al. | 2019 | BMC Public Health | Impact of a bottom-up community engagement intervention on maternal and child health services utilisation in Ghana: a cluster randomised trial | No | Community collaboration | Community quality care champions were trained by external actors, facilitated meetings with community groups to assess quality of services at health facilities, facilitated meetings with facility staff to validate assessments and develop action plans, and ensured actions plans were implemented by facility staff. |
| Alvarado-Castro et al. | 2019 | Popul Health Metr | Social capital is associated with lower mosquito vector indices: secondary analysis from a cluster randomised controlled trial of community mobilisation for dengue prevention in Mexico | Yes | Community empowerment |  |
| Amirkhanian et al. | 2015 | AIDS | Effects of a social network HIV/STD prevention intervention for MSM in Russia and Hungary: a randomised controlled trial | No | Community information-giving or consultation |  |
| Amouzou et al. | 2016 | Am J Trop Med Hyg | Effects of the integrated community case management of childhood illness strategy on child mortality in Ethiopia: a cluster randomised trial | No | Community information-giving or consultation |  |
| Amstutz et al. | 2021 | PLOS Med | Offering ART refill through community health workers versus clinic-based follow-up after home-based same-day ART initiation in rural Lesotho: the VIBRA cluster-randomised clinical trial | No | Community information-giving or consultation |  |
| Andersson et al. | 2015 | BMJ | Evidence based community mobilisation for dengue prevention in Nicaragua and Mexico (Camino Verde, the Green Way): cluster randomised controlled trial | Yes | Community empowerment |  |
| Antunes et al. | 1997 | AIDS | Evaluating an AIDS sexual risk reduction programme for young adults in public night schools in Sao Paulo, Brazil | No | Community information-giving or consultation |  |
| Aragie et al. | 2021 | Am J Trop Med Hyg | Community hand-dug wells for trachoma: a cluster-randomised trial | No | Community information-giving or consultation |  |
| Arnold et al. | 2012 | Afr J Reprod Health | Evaluation of school- and community-based HIV prevention interventions with junior secondary school students in Edo State, Nigeria | No | Not cluster-randomised |  |
| Arunachalam et al. | 2012 | Pathog Glob Health | Community-based control of Aedes aegypti by adoption of eco-health methods in Chennai City, India | No | Community collaboration | Community stakeholders were consulted to understand problems and develop strategies. Women's groups helped external actors to organise meetings, distribute water container covers and health education materials, and implement clean-up campaigns. Neighbourhood associations partnered |
|  |  |  |  |  |  | with external actors for waste disposal and recycling. |
| Atkinson et al. | 2009 | Malar J | A cluster randomised controlled cross-over bed net acceptability and preference trial in Solomon Islands: community participation in shaping policy for malaria elimination | No | Not cluster-randomised |  |
| Ayles et al. | 2013 | Lancet | Effect of household and community interventions on the burden of tuberculosis in southern Africa: the ZAMSTAR community-randomised trial | No | Community information-giving or consultation |  |
| Baqui et al. | 2008 | Lancet | Effect of community-based newborn-care intervention package implemented through two service-delivery strategies in Sylhet district, Bangladesh: a cluster-randomised controlled trial | No | Community information-giving or consultation |  |
| Baqui et al. | 2016 | J Perinatol | Effect of community-based newborn care on cause-specific neonatal mortality in Sylhet district, Bangladesh: findings of a cluster-randomised controlled trial | No | Community information-giving or consultation |  |
| Barzgar et al. | 1997 | World Health Forum | Female health workers boost primary care | No | Not cluster-randomised |  |
| Basso et al. | 2017 | Am J Trop Med Hyg | Scaling up of an innovative intervention to reduce risk of dengue, chikungunya, and zika transmission in Uruguay in the framework of an intersectoral approach with and without community participation | No | Community information-giving or consultation |  |
| Beatty et al. | 2024 | Matern Child Nutr | A cluster randomised controlled trial of a community-based initiative to reduce stunting in rural Indonesia | No | Community collaboration | Community volunteers were trained on community sanitation alongside 'Sanitarians', who were health care providers based at subdistrict clinics. |
| Becker et al. | 2022 | Parasit Vectors | Integrated control of Aedes albopictus in southwest Germany supported by the sterile insect technique | No | Not cluster-randomised |  |
| Bello et al. | 2017 | PLOS One | The effect of engaging unpaid informal providers on case detection and treatment initiation rates for TB and HIV in rural | No | Community information-giving or consultation |  |
|  |  |  | Malawi (Triage Plus): A cluster randomised health system intervention trial |  |  |  |
| Benade et al. | 2023 | PLOS Glob Public Health | Economic evaluation of a cluster randomised, non-inferiority trial of differentiated service delivery models of HIV treatment in Zimbabwe | No | Community information-giving or consultation |  |
| Berkley-Patton et al. | 2019 | AIDS Behav | Feasibility and outcomes of an HIV testing intervention in African American churches | No | Community collaboration | External actors partnered with a faith-based organisation, church leaders, and health agencies to design and implement an HIV testing programme. Church liaisons were trained and coordinated events, using procedures outlined by partners and submitted to health agencies. |
| Bhatia et al. | 2023 | SSM Popul Health | Effects of community youth teams facilitating participatory adolescent groups, youth leadership activities and livelihood promotion to improve school attendance, dietary diversity and mental health among adolescent girls in rural eastern India (JIAH trial): a cluster-randomised controlled trial | No | No CD outcome reported |  |
| Bhatt et al. | 2012 | Malar J | Effectiveness and durability of Interceptor(R) long-lasting insecticidal nets in a malaria endemic area of central India | No | Community information-giving or consultation |  |
| Bigio et al. | 2022 | PLOS Negl Trop Dis | Entomological outcomes of cluster-randomised, community-driven dengue vector-suppression interventions in Kampong Cham province, Cambodia | No | Community collaboration | External actors co-created maps with community members to inform vector control efforts. Education and training were also provided to teachers and community health workers. |
| Binka et al. | 1996 | Trop Med Int Health | Impact of permethrin impregnated bednets on child mortality in Kassena-Nankana district, Ghana: a randomised controlled trial | No | Community information-giving or consultation |  |
| Biran et al. | 2018 | Am J Trop Med Hyg | A cluster-randomised trial to evaluate the impact of an inclusive, community-led total sanitation intervention on sanitation access for people with disabilities in Malawi | Yes | Community empowerment |  |
| Boivin et al. | 2017 | J Dev Behav Pediatr | Effect of caregiver training on the neurodevelopment of HIV-exposed uninfected children and caregiver mental health: a Ugandan cluster-randomised controlled trial | No | No CD outcome reported |  |
| Bonnet et al. | 2023 | Lancet Glob Health | Effectiveness of a community-based approach for the investigation and management of children with household tuberculosis contact in Cameroon and Uganda: a cluster-randomised trial | No | Community information-giving or consultation |  |
| Boone et al. | 2016 | Lancet Glob Health | Effects of community health interventions on under-5 mortality in rural Guinea-Bissau (EPICS): a cluster-randomised controlled trial | No | Community information-giving or consultation |  |
| Borja-Vega et al. | 2014 | Waterlines | The effects of the Total Sanitation and Sanitation Marketing programme on gender and ethnic groups in Indonesia | Yes | Community empowerment |  |
| Bousema et al. | 2016 | PLOS Med | The impact of hotspot-targeted interventions on malaria transmission in Rachuonyo South district in the western Kenyan highlands: a cluster-randomised controlled trial | No | Community information-giving or consultation |  |
| Briceño et al. | 2015 | J Dev Effect | Cost-efficiency of rural sanitation promotion: activity-based costing and experimental evidence from Tanzania | Yes | Community empowerment |  |
| Briceño et al. | 2017 | PLOS One | Are there synergies from combining hygiene and sanitation promotion campaigns? Evidence from a large-scale cluster-randomised trial in rural Tanzania | Yes | Community empowerment |  |
| C.D.I. Study Group | 2010 | Bull World Health Organ | Community-directed interventions for priority health problems in Africa: results of a multicountry study | No | Not cluster-randomised |  |
| Cameron et al. | 2019 | J Dev Econ | Scaling up sanitation: evidence from an RCT in Indonesia | Yes | Community empowerment |  |
| Campos et al. | 2013 | PLOS One | Reaching the unreachable: providing STI control services to female sex workers via mobile team outreach | No | Community information- giving or |  |
|  |  |  |  |  | consultation |  |
| Caprara et al. | 2015 | Trans R Soc Trop Med Hyg | Entomological impact and social participation in dengue control: a cluster randomised trial in Fortaleza, Brazil | No | Community collaboration | Partners, including government agencies, community leaders, and community members, participated in workshops, discussed results of a situational analysis led by external actors, and planned and implemented environmental management activities. |
| Carabin et al. | 2018 | Lancet Glob Health | Effectiveness of a community-based educational programme in reducing the cumulative incidence and prevalence of human Taenia solium cysticercosis in Burkina Faso in 2011-14 (EFECAB): a cluster-randomised controlled trial | No | Community collaboration | Community members attended participatory sessions by external actors that were aimed at improving self-efficacy related to controlling human cysticercosis. Sessions included a movie screening with structured discussion. |
| Carcamo et al. | 2017 | BMC Public Health | Informed community mobilisation for dengue prevention in households with and without a regular water supply: secondary analysis from the Camino Verde trial in Nicaragua | Yes | Community empowerment |  |
| Carlson et al. | 2012 | Soc Sci Med | Enhancing adolescent self-efficacy and collective efficacy through public engagement around HIV/AIDS competence: a multilevel, cluster randomised-controlled trial | No | Community information-giving or consultation |  |
| Caruso et al. | 2022 | Lancet Planet Health | Effect of a low-cost, behaviour-change intervention on latrine use and safe disposal of child faeces in rural Odisha, India: a cluster-randomised controlled trial | No | Community information-giving or consultation |  |
| Castel et al. | 2015 | PLOS One | Comparing cost-effectiveness of HIV testing strategies: targeted and routine testing in Washington, DC | No | Not cluster-randomised |  |
| Castro et al. | 2012 | Trans R Soc Trop Med Hyg | A community empowerment strategy embedded in a routine dengue vector control programme: a cluster randomised controlled trial | No | Community collaboration | Partners, including government agencies, doctors and nurses, and community leaders, formed community working groups for dengue control and conducted needs assessments, developed action plans, implemented |
|  |  |  |  |  |  | strategies, and evaluated implementation. |
| Cha et al. | 2020 | Int J Environ Res Public Health | Benefits and costs of a community-led total sanitation intervention in rural Ethiopia-a trial-based ex post economic evaluation | Yes | Community empowerment |  |
| Cha et al. | 2021 | Am J Trop Med Hyg | Effect of a community-led total sanitation intervention on the incidence and prevalence of diarrhoea in children in rural Ethiopia: a cluster-randomised controlled trial | Yes | Community empowerment |  |
| Chaki et al. | 2012 | Malar J | An affordable, quality-assured community-based system for high-resolution entomological surveillance of vector mosquitoes that reflects human malaria infection risk patterns | No | Not cluster-randomised |  |
| Cham et al. | 2019 | PLOS One | Methods, outcomes, and costs of a 2.5 year comprehensive facility-and community-based HIV testing intervention in Bukoba municipal council, Tanzania, 2014-2017 | No | Not cluster-randomised |  |
| Chanda et al. | 2017 | PLOS Med | HIV self-testing among female sex workers in Zambia: a cluster randomised controlled trial | No | Community information-giving or consultation |  |
| Chandrashekar et al. | 2014 | PLOS One | The costs of scaling up HIV prevention for high risk groups: lessons learned from the Avahan programme in India | No | Not cluster-randomised |  |
| Chang et al. | 2016 | J Acquir Immune Defic Syndr | Implementation and operational research: cost and efficiency of a hybrid mobile multidisease testing approach with high HIV testing coverage in east Africa | No | Community information-giving or consultation |  |
| Chang et al. | 2021 | PLOS Med | Novel community health worker strategy for HIV service engagement in a hyperendemic community in Rakai, Uganda: a pragmatic, cluster-randomised trial | No | Community information-giving or consultation |  |
| Che-Mendoza et al. | 2015 | Trans R Soc Trop Med Hyg | Long-lasting insecticide-treated house screens and targeted treatment of | No | Community information- |  |
|  |  |  | productive breeding-sites for dengue vector control in Acapulco, Mexico |  | giving or consultation |  |
| Chen et al. | 2013 | Cancer Epidemiol Biomarkers Prev | Increasing hepatitis B screening for hmong adults: results from a randomised controlled community-based study | No | Not cluster-randomised |  |
| Choi et al. | 2013 | Can J Infect Dis Med Microbiol | Economic evaluation of community-based HIV prevention programs in Ontario: evidence of effectiveness to reduce HIV infection cases and save costs to medical system | No | Not cluster-randomised |  |
| Clarke et al. | 2017 | BMJ Glob Health | Impact of a malaria intervention package in schools on Plasmodium infection, anaemia and cognitive function in schoolchildren in Mali: a pragmatic cluster-randomised trial | No | Community collaboration | Teachers were trained by external actors to incorporate malaria education into schooling and organised an insecticide treated bednet distribution event. External actors then provided mass treatment to reduce malaria infections and anaemia. |
| Coates et al. | 2014 | Lancet Glob Health | Effect of community-based voluntary counselling and testing on HIV incidence and social and behavioural outcomes (NIMH Project Accept; HPTN 043): a cluster-randomised trial | No | Community collaboration | Community working groups and volunteers liased between external actors and community members, providing input on materials and activities, and implementing community engagement around HIV testing and care. |
| Conteh et al. | 2004 | Trop Med Int Health | The cost and cost-effectiveness of malaria vector control by residual insecticide house-spraying in southern Mozambique: a rural and urban analysis | No | Not cluster-randomised |  |
| Coulibaly et al. | 2020 | Am J Trop Med Hyg | Impact of different intervention strategies on helminth and intestinal protozoa in central Cote D'ivoire | No | Full text not found |  |
| Cowan et al. | 2018 | Lancet HIV | Targeted combination prevention to support female sex workers in Zimbabwe accessing and adhering to antiretrovirals for treatment and prevention of HIV (SAPPH-IRe): a cluster-randomised trial | No | Community information-giving or consultation |  |
| Crocker et al. | 2016 | Environ Sci Technol | Impact evaluation of training natural leaders during a community-led total  sanitation intervention: a cluster-randomised field trial in Ghana | Yes | Community empowerment |  |
| Crocker et al. | 2017 | Int J Hyg Environ Health | Sustainability of community-led total sanitation outcomes: evidence from Ethiopia and Ghana | Yes | Community empowerment |  |
| Crocker et al. | 2017 | Sci Total Environ | The true costs of participatory sanitation: evidence from community-led total sanitation studies in Ghana and Ethiopia | Yes | Community empowerment |  |
| Crocker et al. | 2021 | Int J Hyg Environ Health | Cost effectiveness of community led total sanitation in Ethiopia and Ghana | Yes | Community empowerment |  |
| Croke et al. | 2022 | Trop Med Int Health | Effects of a community-driven water, sanitation, and hygiene programme on COVID-19 symptoms, vaccine acceptance, and non-COVID illnesses: a cluster-randomised controlled trial in rural Democratic Republic of Congo | Yes | Community empowerment |  |
| Dalaba et al. | 2018 | PLOS One | Cost of malaria treatment and health seeking behaviour of children under-five years in the Upper West region of Ghana | No | Not cluster-randomised |  |
| Darmstadt et al. | 2013 | JAMA | behaviour change and community participation: assessing causal pathways affecting neonatal mortality | No | Not research article |  |
| Das et al. | 2014 | Malar J | Strengthening malaria service delivery through supportive supervision and community mobilisation in an endemic Indian setting: an evaluation of nested delivery models | No | Community information-giving or consultation |  |
| Datiko et al. | 2009 | PLOS One | Health extension workers improve tuberculosis case detection and treatment success in southern Ethiopia: a community randomised trial | No | Community information-giving or consultation |  |
| Davis et al. | 2023 | SSM Popul Health | The effect of universal testing and treatment for HIV on health-related quality of life: an analysis of data from the HPTN 071 (PopART) cluster randomised trial | No | Community information-giving or consultation |  |
| De La Cruz et al. | 2009 | Trans R Soc Trop Med Hyg | Microfinance against malaria: impact of Freedom from Hunger's malaria education when delivered by rural banks in Ghana | No | Community information-giving or consultation |  |
| DeGarmo et al. | 2022 | JAMA Netw Open | Effectiveness of a COVID-19 testing outreach intervention for Latinx communities: a cluster randomised trial | No | Community collaboration | External actors partnered and attended meetings with health agencies and community-based organisations to exchange information, plan COVID testing events, and problem solve and share outreach strategies. Community volunteers were recruited and trained by community-based organisations. |
| Deribew et al. | 2012 | Malar J | The effect of household heads training about the use of treated bed nets on the burden of malaria and anaemia in under-five children: a cluster randomised trial in Ethiopia | No | Community collaboration | Community network systems were formed in collaboration with external actors. Networks, which included health agencies and trained community members, attended monthly meetings, monitored and followed-up with household use of insecticide treated bed nets, and addressed implementation challenges. |
| Desrochers et al. | 2014 | Malar J | Effectiveness of post-campaign, door-to-door, hang-up, and communication interventions to increase long-lasting, insecticidal bed net utilisation in Togo (2011-2012): a cluster randomised, controlled trial | No | Community information-giving or consultation |  |
| Deuson et al. | 2001 | Arch Pediatr Adolesc Med | Economic analysis of a child vaccination project among Asian Americans in Philadelphia, PA | No | Not cluster-randomised |  |
| Diallo et al. | 2023 | Epidemiology | Effectiveness of trachomatous trichiasis case-identification approaches in Ethiopia | No | Not cluster-randomised |  |
| Doherty et al. | 2013 | BMJ | Effect of home based HIV counselling and testing intervention in rural South Africa: cluster randomised trial | No | Community information-giving or consultation |  |
| Dolcini et al. | 2010 | Health Educ Behav | Project ORE: a friendship-based intervention to prevent HIV/STI in urban African American adolescent females | no | Community information-giving or consultation |  |
| Doyle et al. | 2010 | PLOS Med | Long-term biological and behavioural impact of an adolescent sexual health intervention in Tanzania: follow-up survey of the community-based MEMA kwa Vijana trial | No | Community collaboration | Teachers were trained by external actors and delivered structured sexual health education. |
| Duby et al. | 2020 | Glob Health Action | Effect of an integrated neonatal care kit on cause-specific neonatal mortality in rural Pakistan | No | Community information-giving or consultation |  |
| Enbiale et al. | 2020 | J Infect Dev Ctries | Stopping the itch: mass drug administration for scabies outbreak control covered for over nine million people in Ethiopia | No | Not cluster-randomised |  |
| Eriksen et al. | 2010 | Afr Health Sci | Effectiveness of a community intervention on malaria in rural Tanzania: a randomised controlled trial | No | Community information-giving or consultation |  |
| Ezeanolue et al. | 2015 | Lancet Glob Health | Effect of a congregation-based intervention on uptake of HIV testing and linkage to care in pregnant women in Nigeria (Baby Shower): a cluster randomised trial | No | Community information-giving or consultation |  |
| Ezeanolue et al. | 2017 | AIDS Behav | What do you need to get male partners of pregnant women tested for HIV in resource limited settings? The baby shower cluster randomised trial | No | Community information-giving or consultation |  |
| Fang et al. | 2019 | J Community Health | Community-based cervical cancer education: changes in knowledge and beliefs among Vietnamese American women | No | NCD |  |
| Favre et al. | 2015 | Acta Trop | School-based and community-based actions for scaling-up diagnosis and treatment of schistosomiasis toward its elimination in an endemic area of Brazil | No | Community information-giving or consultation |  |
| Feasey et al. | 2023 | PLOS Glob Public Health | Impact of active case-finding for tuberculosis on case-notifications in Blantyre, Malawi: a community-based cluster-randomised trial (SCALE) | No | Community information-giving or consultation |  |
| Feldblum et al. | 2001 | AIDS | Female condom introduction and sexually transmitted infection prevalence: results of a community intervention trial in Kenya | No | Community information-giving or consultation |  |
| Fernandez et al. | 1998 | Acta Trop | Trial of a community-based intervention to decrease infestation of Aedes aegypti mosquitoes in cement washbasins in El Progreso, Honduras | No | Not cluster-randomised |  |
| Ferral et al. | 2010 | Am J Trop Med Hyg | Comparative field trial of alternative vector control strategies for non-domiciliated Triatoma dimidiata | No | Not cluster-randomised |  |
| Figueroa et al. | 2010 | Trop Med Int Health | The challenge of promoting safe sex at sites where persons meet new sex partners in Jamaica: results of the Kingston PLACE randomised controlled trial | No | Community information-giving or consultation |  |
| Fisher et al. | 2023 | Lancet Child Adolesc Health | Structured, multicomponent, community-based programme for women's health and infant health and development in rural Vietnam: a parallel-group cluster randomised controlled trial | No | No CD outcome reported |  |
| Forsyth et al. | 2022 | PLOS Negl Trop Dis | Larval source reduction with a purpose: designing and evaluating a household- and school-based intervention in coastal Kenya | No | Community collaboration | External actors held stakeholder workshops to collaboratively design the intervention, including developing a logic model, identifying larval source reduction recommendations, and developing activities to generate awareness and motivate adoption of source-reduction behaviours. |
| Forsythe et al. | 2019 | Afr J AIDS Res | HIV treatment support services in Tanzania: a cost and efficiency analysis at facility and community levels | No | Not cluster-randomised |  |
| Fottrell et al. | 2013 | JAMA Pediatr | The effect of increased coverage of participatory women's groups on neonatal | No | No CD outcome reported |  |
|  |  |  | mortality in Bangladesh: A cluster randomised trial |  |  |  |
| Galai et al. | 2018 | Soc Sci Med | A cluster randomised trial of community mobilisation to reduce methamphetamine use and HIV risk among youth in Thailand: design, implementation and results | No | Community collaboration | Partners, including government agencies, police officials, religious leaders, and community representatives, formed community coalitions for methamphetamine use and HIV and sexually transmitted infection risk. External actors facilitated meetings with coalitions to define objectives and design and implement strategies. |
| Garcia et al. | 2012 | Lancet | Prevention of sexually transmitted infections in urban communities (Peru PREVEN): a multicomponent community-randomised controlled trial | No | Community information-giving or consultation |  |
| Garcia-Zapata et al. | 1988 | Rev Argent Microbiol | Epidemiological vigilance with community participation in the control of the vectors of Chagas' disease in Goias, Central Brazil | No | Full text not found |  |
| Geldsetzer et al. | 2019 | PLOS Med | Community health workers to improve uptake of maternal health care services: a cluster-randomised pragmatic trial in Dar es Salaam, Tanzania | No | Community information-giving or consultation |  |
| Gladstone et al. | 2019 | PLOS Med | Independent and combined effects of improved water, sanitation, and hygiene and improved complementary feeding on early neurodevelopment among children born to HIV-negative mothers in rural Zimbabwe: substudy of a cluster-randomised trial | No | Community information-giving or consultation |  |
| Goel et al. | 2021 | J Vector Borne Dis | Community sensitisation to improve acceptability of indoor residual spraying in Mewat district of Haryana, India: a community-based interventional study | No | Community information-giving or consultation |  |
| Goodman et al. | 2006 | Health Policy Plan | The cost-effectiveness of improving malaria home management: shopkeeper training in rural Kenya | No | Not cluster-randomised |  |
| Gottschlich et al. | 2023 | Nat Med | Community-integrated self-collected HPV-based cervix screening in a low-resource rural setting: a pragmatic, cluster-randomised trial | No | Community information-giving or consultation |  |
| Gouteux et al. | 1990 | Trop Med Parasitol | Community participation in the control of tsetse flies. Large scale trials using the pyramid trap in the Congo | No | Not cluster-randomised |  |
| Gowelo et al. | 2023 | Am J Trop Med Hyg | Community participation in habitat management and larviciding for the control of malaria vectors in southern Nalawi | Yes | Community empowerment |  |
| Gowelo et al. | 2020 | Malar J | Community factors affecting participation in larval source management for malaria control in Chikwawa district, southern Malawi | Yes | Community empowerment |  |
| Gupta et al. | 1992 | Indian J Malariol | Intradomestic mosquito breeding sources and their management | No | Not cluster-randomised |  |
| Habib et al. | 2017 | Lancet Glob Health | Community engagement and integrated health and polio immunisation campaigns in conflict-affected areas of Pakistan: a cluster randomised controlled trial | No | Community information-giving or consultation |  |
| Habib et al. | 2023 | Vaccines | Does IPV boost intestinal immunity among children under five years of age? An experience from Pakistan | No | Community information-giving or consultation |  |
| Haggerty et al. | 1994 | Int J Epidemiol | Community-based hygiene education to reduce diarrhoeal disease in rural Zaire: impact of the intervention on diarrhoeal morbidity | No | Community information-giving or consultation |  |
| Hall et al. | 2022 | J Viral Hepat | Evaluating the cost-effectiveness of hepatitis B vaccination strategies in high-impact settings for adults | No | Not cluster-randomised |  |
| Halliday et al. | 2020 | BMJ Glob Health | Impact of school-based malaria case management on school attendance, health and education outcomes: a cluster randomised trial in southern Malawi | No | Community information-giving or consultation |  |
| Halwindi et al. | 2011 | Parasitol | Impact of community-directed treatment on soil transmitted helminth infections in | No | Not cluster-randomised |  |
|  |  |  | children aged 12 to 59 months in Mazabuka district, Zambia |  |  |  |
| Hamainza et al. | 2014 | Malar J | Monitoring, characterization and control of chronic, symptomatic malaria infections in rural Zambia through monthly household visits by paid community health workers | No | Not cluster-randomised |  |
| Han et al. | 2013 | J Epidemiol Community Health | Family economic empowerment and mental health among AIDS-affected children living in AIDS-impacted communities: evidence from a randomised evaluation in southwestern Uganda | No | No CD outcome reported |  |
| Harding-Esch et al. | 2013 | PLOS Negl Trop Dis | Mass treatment with azithromycin for trachoma: when is one round enough? Results from the PRET trial in the Gambia | No | Community information-giving or consultation |  |
| Harris-Fry et al. | 2016 | J Epidemiol Community Health | Formative evaluation of a participatory women's group intervention to improve reproductive and women's health outcomes in rural Bangladesh: a controlled before and after study | No | Not cluster-randomised |  |
| Hensen et al. | 2023 | PLOS Med | The impact of community-based, peer-led sexual and reproductive health services on knowledge of HIV status among adolescents and young people aged 15 to 24 in Lusaka, Zambia: the Yathu Yathu cluster-randomised trial | No | Community information-giving or consultation |  |
| Hensen et al. | 2023 | Reprod Health | Does distribution of menstrual products through community-based, peer-led sexual and reproductive health services increase use of appropriate menstrual products? Findings from the Yathu Yathu trial | No | Community information-giving or consultation |  |
| Hii et al. | 1995 | Southeast Asian J Trop Med Public Health | Lambdacyhalothrin impregnated bednets control malaria in Sabah, Malaysia | No | Not cluster-randomised |  |
| Hislop et al. | 2006 | Trends Cancer | An intervention to promote cervical cancer screening among Chinese women in North America | No | Full text not found |  |
| Huchko et al. | 2018 | Int J Gynaecol Obstet | Cervical cancer screening through human papillomavirus testing in community health campaigns versus health facilities in rural western Kenya | No | Community information-giving or consultation |  |
| Humphrey et al. | 2019 | Lancet Glob Health | Independent and combined effects of improved water, sanitation, and hygiene, and improved complementary feeding, on child stunting and anaemia in rural Zimbabwe: a cluster-randomised trial | No | Community information-giving or consultation |  |
| Hustedt et al. | 2021 | Am J Trop Med Hyg | Field efficacy of larvivorous fish and pyriproxyfen combined with community engagement on dengue vectors in Cambodia: a randomised controlled trial | No | Community collaboration | External actors co-created communication materials with community members to inform vector control efforts. Education and training were also provided to community health workers. |
| Indravudh et al. | 2021 | BMJ Glob Health | Pragmatic economic evaluation of community-led delivery of HIV self-testing in Malawi | Yes | Community empowerment |  |
| Indravudh et al. | 2021 | PLOS Med | Effect of community-led delivery of HIV self-testing on HIV testing and antiretroviral therapy initiation in Malawi: a cluster-randomised trial | Yes | Community empowerment |  |
| Indravudh et al. | 2022 | PLOS Glob Public Health | Understanding mechanisms of impact from community-led delivery of HIV self-testing: mediation analysis of a cluster-randomised trial in Malawi | Yes | Community empowerment |  |
| Jimenez-Alejo et al. | 2017 | BMC Public Health | Pupal productivity in rainy and dry seasons: findings from the impact survey of a randomised controlled trial of dengue prevention in Guerrero, Mexico | Yes | Community empowerment |  |
| Johnson et al. | 2019 | Sci Rep | Optimal HIV testing strategies for South Africa: a model-based evaluation of population-level impact and cost-effectiveness | No | Not cluster-randomised |  |
| Jurgensen et al. | 2013 | Soc Sci Med | Effects of home-based voluntary counselling and testing on HIV-related stigma: findings from a cluster-randomised trial in Zambia | No | Community information-giving or consultation |  |
| Kakai et al. | 2009 | J Infect Dev Ctries | Supporting formal education to improve quality of health care provided by mothers of children with malaria in rural western Kenya | No | Not cluster-randomised |  |
| Kalichman et al. | 2008 | Ann Behav Med | Randomised trial of a community-based alcohol-related HIV risk-reduction intervention for men and women in Cape Town, South Africa | No | Not cluster-randomised |  |
| Kalichman et al. | 2014 | Eur J Public Health | Randomised community-level HIV prevention intervention trial for men who drink in South African alcohol-serving venues | No | Community information-giving or consultation |  |
| Kamali et al. | 2003 | Lancet | Syndromic management of sexually-transmitted infections and behaviour change interventions on transmission of HIV-1 in rural Uganda: a community randomised trial | No | Community information-giving or consultation |  |
| Katabarwa et al. | 1999 | Ann Trop Med Parasitol | The community-directed, ivermectin-treatment programme for onchocerciasis control in Uganda - an evaluative study (1993-1997) | No | Full text not found |  |
| Kaufman et al. | 2014 | Am J Public Health | Effectiveness of Circle of Life, an HIV-preventive intervention for American Indian middle school youths: a group randomised trial in a Northern Plains tribe | No | Community information-giving or consultation |  |
| Kaunda-Khangamwa et al. | 2019 | BMC Health Serv Res | The role of health animators in malaria control: a qualitative study of the health animator approach within the Majete malaria project in Chikwawa district, Malawi | Yes | Community empowerment |  |
| Kay et al. | 2010 | Am J Trop Med Hyg | Sustainability and cost of a community-based strategy against Aedes aegypti in northern and central Vietnam | No | Not cluster-randomised |  |
| Keating et al. | 2011 | Acta Trop | Evaluating indoor residual spray for reducing malaria infection prevalence in Eritrea: results from a community randomised control trial | No | Community information-giving or consultation |  |
| Keenan et al. | 2019 | PLOS Negl Trop Dis | Linear growth in preschool children treated with mass azithromycin distributions for trachoma: a cluster-randomised trial | No | Community information-giving or consultation |  |
| Kelly et al. | 1991 | Am J Public Health | HIV risk behaviour reduction following intervention with key opinion leaders of population: an experimental analysis | No | Not cluster-randomised |  |
| Kerrigan et al. | 2019 | J Acquir Immune Defic Syndr | Project Shikamana: community empowerment-based combination HIV prevention significantly impacts HIV incidence and care continuum outcomes among female sex workers in Iringa, Tanzania | No | Not cluster-randomised |  |
| Khin Thet et al. | 2012 | Pathog Glob Health | Community-centred eco-bio-social approach to control dengue vectors: an intervention study from Myanmar | No | Community collaboration | Partners, including government agencies, midwives, and community representatives, formed ecofriendly groups for dengue control and set targets, organised community members to accept environmental management strategies, and selected and supervised activities by volunteers. |
| Kim et al. | 2007 | Am J Public Health | Understanding the impact of a microfinance-based intervention on women's empowerment and the reduction of intimate partner violence in South Africa | No | Community collaboration | Microfinance groups participated in trainings by external actors. Groups were collaboratively involved in action planning and implementation by staff at microfinance loan centres for community mobilisation events. |
| Kim et al. | 2015 | PLOS Negl Trop Dis | Financial and economic costs of the elimination and eradication of onchocerciasis (river blindness) in Africa | No | Not cluster-randomised |  |
| Kipp et al. | 1998 | Health Policy Plan | Ivermectin distribution using community volunteers in Kabarole district, Uganda | No | Not cluster-randomised |  |
| Kirby et al. | 2004 | J Adolesc Health | The "Safer Choices" intervention: its impact on the sexual behaviours of different subgroups of high school students | No | Community information-giving or consultation |  |
| Kittayapong et al | 2006 | J Am Mosq Control Assoc | Community participation and appropriate technologies for dengue vector control at transmission foci in Thailand | No | Not cluster-randomised |  |
| Kittayapong et al | 2012 | Pathog Glob Health | Application of eco-friendly tools and eco-bio-social strategies to control dengue vectors in urban and peri-urban settings in Thailand | No | Pilot or interim studies |  |
| Klinkenberg et al. | 2023 | PLOS Med | Tuberculosis prevalence after 4 years of population-wide systematic TB symptom screening and universal testing and treatment for HIV in the HPTN 071 (PopART) community-randomised trial in Zambia and South Africa: a cross-sectional survey (TREATS) | No | Community information-giving or consultation |  |
| Kohler et al. | 2016 | Int J STD AIDS | Sexually transmitted infection screening uptake and knowledge of sexually transmitted infection symptoms among female sex workers participating in a community randomised trial in Peru | No | Community information-giving or consultation |  |
| Kolaczinski et al. | 2010 | Malar J | Costs and effects of two public sector delivery channels for long-lasting insecticidal nets in Uganda | No | Not cluster-randomised |  |
| Kouyate et al. | 2008 | Malar J | Process and effects of a community intervention on malaria in rural Burkina Faso: randomised controlled trial | No | Community information-giving or consultation |  |
| Kroeger et al. | 1995 | Am J Trop Med Hyg | Insecticide-impregnated bed nets for malaria control: varying experiences from Ecuador, Colombia, and Peru concerning acceptability and effectiveness | No | Community information-giving or consultation |  |
| Kuteesa et al. | 2019 | PLOS One | Feasibility of conducting HIV combination prevention interventions in fishing communities in Uganda: a pilot cluster randomised trial | No | Pilot or interim studies |  |
| Kweku et al. | 2009 | PLOS One | Options for the delivery of intermittent preventive treatment for malaria to children: a community randomised trial | No | Community information-giving or consultation |  |
| Kyaw et al. | 2021 | BMC Public Health | Estimating the programmatic cost of targeted mass drug administration for malaria in Myanmar | No | Not cluster-randomised |  |
| Kyegombe et al. | 2014 | J Int AIDS Soc | The impact of SASA!, a community mobilisation intervention, on reported HIV-related risk behaviours and relationship dynamics in Kampala, Uganda | Yes | Community empowerment |  |
| Kyegombe et al. | 2014 | Glob Health Action | SASA! is the medicine that treats violence'. Qualitative findings on how a community mobilisation intervention to prevent violence against women created change in Kampala, Uganda | Yes | Community empowerment |  |
| Labhardt et al. | 2014 | PLOS Med | Home-based versus mobile clinic HIV testing and counseling in rural Lesotho: a cluster-randomised trial | No | Community information-giving or consultation |  |
| Laithavewat et al. | 2020 | Glob Health Promot | Analysis of a school-based health education model to prevent opisthorchiasis and cholangiocarcinoma in primary school children in northeast Thailand | No | Community information-giving or consultation |  |
| Larson et al. | 2015 | PLOS One | How much does it cost to improve access to voluntary medical male circumcision among high-risk, low-income communities in Uganda? | No | Not cluster-randomised |  |
| LeCroy et al. | 2018 | Health Educ Behav | Go Grrrls: a randomised controlled trial of a gender-specific intervention to reduce sexual risk factors in middle school females | No | Not cluster-randomised |  |
| Legorreta-Soberanis et al. | 2017 | BMC Public Health | Coverage and beliefs about temephos application for control of dengue vectors and impact of a community-based prevention intervention: secondary analysis from the Camino Verde trial in Mexico | Yes | Community empowerment |  |
| Legorreta-Soberanis et al. | 2017 | BMC Public Health | Household costs for personal protection against mosquitoes: secondary outcomes from a randomised controlled trial of dengue prevention in Guerrero state, Mexico | Yes | Community empowerment |  |
| Legorreta-Soberanis et al. | 2017 | BMC Public Health | Household costs of dengue illness: secondary outcomes from a randomised controlled trial of dengue prevention in Guerrero state, Mexico | Yes | Community empowerment |  |
| Lenk et al. | 2020 | Clin Infect Dis | A test-and-not-treat strategy for onchocerciasis elimination in Loa loa-coendemic areas: cost analysis of a pilot in the Soa health district, Cameroon | No | Not cluster-randomised |  |
| Leontsini et al. | 1993 | Trans R Soc Trop Med Hyg | Effect of a community-based Aedes aegypti control programme on mosquito larval production sites in El Progreso, Honduras | No | Community collaboration | External actors engaged community leaders and members, who formed health committees to address dengue control. Committees met and planned activities with support from external actors and were trained on environmental management. Committee members provided information to households and surveyed the infrastructure, often accompanied by external actors. |
| Lewycka et al. | 2013 | Lancet | Effect of women's groups and volunteer peer counselling on rates of mortality, morbidity, and health behaviours in mothers and children in rural Malawi (MaiMwana): a factorial, cluster-randomised controlled trial | Yes | Community empowerment |  |
| Li et al. | 2022 | AIDS Behav | Community capacity building for HIV and addiction service integration: an intervention trial in Vietnam | No | Community information-giving or consultation |  |
| Li et al. | 2022 | Int J STD AIDS | A community approach to promote healthcare services for people living with HIV who use drugs in Vietnam | No | Community information-giving or consultation |  |
| Lietman et al. | 2020 | Am J Ophthalmol | Frequency of mass azithromycin distribution for ocular chlamydia in a trachoma endemic region of Ethiopia: a cluster randomised trial | No | Community information-giving or consultation |  |
| Limbada et al. | 2022 | Lancet HIV | Rates of viral suppression in a cohort of people with stable HIV from two community models of ART delivery versus facility-based HIV care in Lusaka, Zambia: a cluster-randomised, non-inferiority trial nested in the HPTN 071 (PopART) trial | No | Community information-giving or consultation |  |
| Lin et al. | 2023 | J Med Internet Res | Digital, crowdsourced, multilevel intervention to promote HIV testing among men who have sex with men: cluster randomised controlled trial | No | Community collaboration | The intervention was developed through a series of crowdsourcing open calls that were tailored for men who have sex with men and solicited by external actors. |
| Lippman et al. | 2017 | J Acquir Immune Defic Syndr | Community mobilisation for HIV testing uptake: results from a community randomised trial of a theory-based intervention in rural South Africa | No | Community collaboration | A team of supervisors, community mobilisers, and community action teams delivered mobilisation activities, which were implemented with systematic targets. |
| Lippman et al. | 2022 | Lancet HIV | A community mobilisation intervention to improve engagement in HIV testing, linkage to care, and retention in care in South Africa: a cluster-randomised controlled trial | No | Community collaboration | A team of supervisors, community mobilisers, and community action teams delivered mobilisation activities, which were implemented with systematic targets. |
| Loum et al. | 2017 | Am J Trop Med Hyg | Evaluation of community-directed operation of black fly traps for entomological surveillance of onchocerca volvulus transmission in the madi-mid north focus of onchocerciasis in northern Uganda | No | Not cluster-randomised |  |
| Loum et al. | 2019 | PLOS Negl Trop Dis | Optimisation and evaluation of the Esperanza window trap to reduce biting rates of Simulium damnosum sensu lato in northern Uganda | No | Not cluster-randomised |  |
| Low et al. | 2013 | AIDS Care | The effects of home-based HIV counseling and testing on HIV/AIDS stigma among individuals and community leaders in western Kenya: evidence from a cluster-randomised trial | No | Community information-giving or consultation |  |
| Ma et al. | 2018 | Cancer | Efficacy of a community-based participatory and multi-level intervention to enhance hepatitis B virus screening and vaccination in underserved Korean Americans | No | Community collaboration | Community advisory boards were formed in collaboration with external actors for HPV screening and acted as liaisons between church members and external actors. Boards, which included church representatives, health care providers, and community-based organisation representatives, attended regular planning meetings and were actively involved in the development and implementation of strategies and materials. |
| Ma et al. | 2019 | PLOS Med | Evaluating the impact of community health volunteer home visits on child diarrhoea and fever in the Volta region, Ghana: a cluster-randomised controlled trial | No | Community information-giving or consultation |  |
| Macleod et al. | 2022 | Lancet HIV | Viral suppression and self-reported ART adherence after 3 years of universal testing and treatment in the HPTN 071 (PopART) community-randomised trial in Zambia and South Africa: a cross-sectional analysis | No | Community information-giving or consultation |  |
| Mafirakureva et al. | 2023 | Lancet Glob Health | Cost-effectiveness of community-based household tuberculosis contact management for children in Cameroon and Uganda: a modelling analysis of a cluster-randomised trial | No | Community information-giving or consultation |  |
| Mageda et al. | 2023 | BMC Public Health | Effectiveness of a community-based intervention (Konga model) to address factors contributing to low viral load suppression among children living with HIV in Tanzania: a preliminary, cluster, randomised clinical trial report | No | Community information-giving or consultation |  |
| Makaula et al. | 2019 | BMC Health Serv Res | Strengthening primary health care at district-level in Malawi: determining the coverage, costs and benefits of community-directed interventions | Yes | Community empowerment |  |
| Makofane et al. | 2023 | J Int AIDS Soc | Impact of family networks on uptake of health interventions: evidence from a community-randomised controlled trial aimed at increasing HIV testing in South Africa | No | Community information-giving or consultation |  |
| Malenga et al. | 2017 | Glob Health | Malaria control in rural Malawi: implementing peer health education for behaviour change | Yes | Community empowerment |  |
| Manaseki-Holland et al. | 2021 | PLOS Med | Effects on childhood infections of promoting safe and hygienic complementary-food handling practices through a community-based programme: a cluster randomised controlled trial in a rural area of the Gambia | No | Community information-giving or consultation |  |
| Mangenah et al. | 2019 | J Int AIDS Soc | Economic cost analysis of door-to-door community-based distribution of HIV self-test kits in Malawi, Zambia and Zimbabwe | No | Community information-giving or consultation |  |
| Mankadi et al. | 2021 | Int J Environ Res Public Health | Effects of door-to-door hang-up visits on the use of long-lasting insecticide-treated mosquito nets in the Democratic Republic of the Congo: a cluster randomised controlled trial | No | Community information-giving or consultation |  |
| Mantra et al. | 1992 | Southeast Asian J Trop Med Public Health | The role of community participation in the malaria control programme in Indonesia | No | Full text not found |  |
| Martinez-Ibarra et al. | 2012 | J Vector Ecol | Combining two teaching techniques for young children on Aedes aegypti control: effects on entomological indices in western Mexico | No | Community information-giving or consultation |  |
| Massa et al. | 2009 | Trans R Soc Trop Med Hyg | The effect of the community-directed treatment approach versus the school-based treatment approach on the prevalence and intensity of schistosomiasis and soil-transmitted helminthiasis among schoolchildren in Tanzania | Yes | Community empowerment |  |
| Massa et al. | 2009 | J Biosoc Sci | Community perceptions on the community-directed treatment and school-based approaches for the control of schistosomiasis and soil-transmitted helminthiasis among school-age children in Lushoto district, Tanzania | Yes | Community empowerment |  |
| Massa et al. | 2009 | Parasitol | Can coverage of schistosomiasis and soil transmitted helminthiasis control programmes targeting school-aged children be improved? New approaches | Yes | Community empowerment |  |
| Matovu et al. | 2014 | Malar J | Household health care-seeking costs: experiences from a randomised, controlled trial of community-based malaria and pneumonia treatment among under-fives in eastern Uganda | No | Community information-giving or consultation |  |
| Matowo et al. | 2023 | Lancet Planet Health | Differential impact of dual-active ingredient long-lasting insecticidal nets on primary malaria vectors: a secondary analysis of a 3-year, single-blind, cluster-randomised controlled trial in rural Tanzania | No | Community information-giving or consultation |  |
| Mazumder et al. | 2014 | BMJ | Effect of implementation of integrated management of neonatal and childhood illness programme on treatment seeking practices for morbidities in infants: cluster randomised trial | No | Community information-giving or consultation |  |
| Mbonye et al. | 2008 | Public Health | Effect of a community-based delivery of intermittent preventive treatment of malaria in pregnancy on treatment seeking for malaria at health units in Uganda | No | Not cluster-randomised |  |
| McCann et al. | 2021 | Malar J | The effect of community-driven larval source management and house improvement on malaria transmission when added to the standard malaria control strategies in Malawi: a cluster-randomised controlled trial | Yes | Community empowerment |  |
| McGrawa et al. | 2013 | Int J Humanit Soc Sci | Promoting breast and cervical cancer screening among Hispanic and African American women | No | NCD |  |
| McGuigan et al. | 2011 | Environ Sci Technol | High compliance randomised controlled field trial of solar disinfection of drinking water and its impact on childhood diarrhoea in rural Cambodia | No | Not cluster-randomised |  |
| McPhee et al. | 2003 | Pediatrics | Successful promotion of hepatitis B vaccinations among Vietnamese-American children ages 3 to 18: results of a controlled trial | No | Not cluster-randomised |  |
| Mendoza-Cano et al. | 2017 | Int J Environ Res Public Health | Cost-effectiveness of the strategies to reduce the incidence of dengue in Colima, Mexico | No | Community information-giving or consultation |  |
| Mengistie et al. | 2013 | PLOS One | Household water chlorination reduces incidence of diarrhoea among under-five children in rural Ethiopia: a cluster randomised controlled trial | No | Community information-giving or consultation |  |
| Michaels-Igbokwe et al. | 2016 | BMC Public Health | Cost and cost-effectiveness analysis of a community mobilisation intervention to reduce intimate partner violence in Kampala, Uganda | Yes | Community empowerment |  |
| Minakawa et al. | 2021 | Am J Trop Med Hyg | Long-lasting insecticidal nets incorporating piperonyl butoxide reduce the risk of malaria in children in western Kenya: a cluster randomised controlled trial | No | Community information-giving or consultation |  |
| Mitchell-Foster et al. | 2015 | Trans R Soc Trop Med Hyg | Integrating participatory community mobilisation processes to improve dengue prevention: an eco-bio-social scaling up of local success in Machala, Ecuador | No | Community information-giving or consultation |  |
| Morozoff et al. | 2022 | BMJ Open | Costs of community-wide mass drug administration and school-based deworming for soil-transmitted helminths: evidence from a randomised controlled trial in Benin, India and Malawi | No | Community information-giving or consultation |  |
| Mosha et al. | 1992 | Med Vet Entomol | Efficacy of Esbiothrin mosquito coils at community level in northern Tanzania | No | Not cluster-randomised |  |
| Mpangala et al. | 2021 | BMC Public Health | On the cost-effectiveness of insecticide-treated wall liner and indoor residual spraying as additions to insecticide treated bed nets to prevent malaria: findings from cluster randomised trials in Tanzania | No | Community information-giving or consultation |  |
| Mutero et al. | 2020 | Malar J | Evaluating the impact of larviciding with Bti and community education and mobilisation as supplementary integrated vector management interventions for malaria control in Kenya and Ethiopia | No | Community information-giving or consultation |  |
| Nair et al. | 2017 | Lancet Glob Health | Effect of participatory women's groups and counselling through home visits on children's linear growth in rural eastern India (CARING trial): a cluster-randomised controlled trial | Yes | Community empowerment |  |
| Nance et al. | 2017 | PLOS One | Short-term effectiveness of a community health worker intervention for HIV-infected pregnant women in Tanzania to improve treatment adherence and retention in care: a cluster-randomised trial | No | Community information-giving or consultation |  |
| Nanyonjo et al. | 2015 | BMC Health Serv Res | Estimating the cost of referral and willingness to pay for referral to higher-level health facilities: a case series study from an integrated community case management programme in Uganda | No | Not cluster-randomised |  |
| Negin et al. | 2009 | Trop Med Int Health | Feasibility, acceptability and cost of home-based HIV testing in rural Kenya | No | Not cluster-randomised |  |
| Nevill et al. | 1996 | Trop Med Int Health | Insecticide-treated bednets reduce mortality and severe morbidity from malaria among children on the Kenyan coast | No | Community information-giving or consultation |  |
| Newell et al. | 2006 | Lancet | Family-member DOTS and community DOTS for tuberculosis control in Nepal: cluster-randomised controlled trial | No | Community information-giving or consultation |  |
| Newton-Sanchez et al. | 2020 | Int J Public Health | Effect of an ecosystem-centered community participation programme on the incidence of dengue: a field randomised, controlled trial | No | Community collaboration | Partners, including stakeholders, community leaders, and other stakeholders, formed focal groups for dengue control and discussed problems, defined strategies that were adapted to each area, and implemented actions. |
| Nganda et al. | 2003 | Int J Tuberc Lung Dis | Cost and cost-effectiveness of increased community and primary care facility involvement in tuberculosis care in Machakos district, Kenya | No | Not cluster-randomised |  |
| NIMH Collaborative HIV/STD Prevention Trial Group | 2010 | J Acquir Immune Defic Syndr | Results of the NIMH collaborative HIV/sexually transmitted disease prevention trial of a community popular opinion leader intervention | No | Community information-giving or consultation |  |
| Odhiambo et al. | 2023 | Soc Sci Med | Comparing the effect of a multisectoral agricultural intervention on HIV-related health outcomes between widowed and married women | No | Community information-giving or consultation |  |
| Okere et al. | 2021 | J Int AIDS Soc | Patient-incurred costs in a differentiated service delivery club intervention compared to standard clinical care in northwest Tanzania | No | Not cluster-randomised |  |
| Okonofua et al. | 2003 | Int J of Infect Dis | Impact of an intervention to improve treatment-seeking behaviour and prevent sexually transmitted diseases among Nigerian youths | No | Community collaboration | Health clubs, which were established by external actors, mounted STI awareness campaigns in schools as well as organised other activities including debates, dramas, essay writing, symposia, and film shows. Members were also selected as peer educators and trained by external actors to provide counselling and distribute informational materials. |
| Oladepo et al. | 1996 | Patient Educ Couns | Outcome of two patient education methods on recruitment and compliance with ivermectin in the treatment of onchocerciasis | No | Community information-giving or consultation |  |
| Onwujekwe et al. | 2001 | Acta Tropica | Onchocerciasis control in Nigeria: will households be able to afford community-directed treatment with ivermectin? | No | Not cluster-randomised |  |
| Onwujekwe et al. | 2002 | Health Policy | Community-directed treatment with ivermectin in two Nigerian communities: an analysis of first year start-up processes, costs and consequences | No | Not cluster-randomised |  |
| Orgill-Meyer et al. | 2019 | Bull World Health Organ | Long-term impact of a community-led sanitation campaign in India, 2005-2016 | No | Community collaboration | Community members participated in 'triggering' exercises by external actors and committed to sanitation improvement. External actors worked closely with community-based organisations and government agencies to establish accessible latrine production centres in villages, raise awareness about subsidies for latrine building, and helped the community to establish systems to punish those who defecated in the open. |
| Ortblad et al. | 2017 | PLOS Med | Direct provision versus facility collection of HIV self-tests among female sex workers in Uganda: a cluster-randomised controlled health systems trial | No | Community information-giving or consultation |  |
| Outlaw et al. | 2010 | Am J Public Health | Using motivational interviewing in HIV field outreach with young African American men who have sex with men: a randomised clinical trial | No | Not cluster-randomised |  |
| Overgaard et al. | 2016 | PLOS Negl Trop Dis | A cluster-randomised controlled trial to reduce diarrhoeal disease and dengue entomological risk factors in rural primary schools in Colombia | No | Community information-giving or consultation |  |
| Oyo-Ita et al. | 2021 | PLOS One | Cost-effectiveness analysis of an intervention project engaging traditional and religious leaders to improve uptake of childhood immunisation in southern Nigeria | No | Community collaboration | Ward development committees consisting of traditional and religious leaders attended trainings aimed at improving leadership and knowledge on vaccination. Ward committees then provided education on vaccination during routine community meetings. |
| Oyo-Ita et al. | 2021 | PLOS One | Effects of engaging communities in decision-making and action through traditional and religious leaders on vaccination coverage in Cross River state, Nigeria: a cluster-randomised control trial | No | Community collaboration | Ward development committees consisting of traditional and religious leaders attended trainings aimed at improving leadership and knowledge on vaccination. Ward committees then provided education on vaccination during routine community meetings. |
| Paramita et al | 2017 | J Public Health | Evaluation of a community-based intervention to improve routine childhood vaccination uptake among migrants in urban slums of Ludhiana, India | No | Not cluster-randomised |  |
| Patouillard et al. | 2011 | PLOS One | Coverage, adherence and costs of intermittent preventive treatment of malaria in children employing different delivery strategies in Jasikan, Ghana | No | Community information-giving or consultation |  |
| Pence et al. | 2007 | Scand J Public Health | The effect of community nurses and health volunteers on child mortality: the Navrongo Community Health and Family Planning project | No | Not cluster-randomised |  |
| Pettifor et al. | 2018 | J Int AIDS Soc | Community mobilisation to modify harmful gender norms and reduce HIV risk: results from a community cluster randomised trial in South Africa | No | Community collaboration | A team of supervisors, community mobilisers, and community action teams delivered mobilisation activities, which were implemented with systematic targets. |
| Phiri et al. | 2021 | Malar J | Cost of community-led larval source management and house improvement for malaria control: a cost analysis within a cluster-randomised trial in a rural district in Malawi | Yes | Community empowerment |  |
| Pickering et al. | 2015 | Lancet Glob Health | Effect of a community-led sanitation intervention on child diarrhoea and child growth in rural Mali: a cluster-randomised controlled trial | Yes | Community empowerment |  |
| Pinkerton et al. | 1998 | Am J Public Health | Cost-effectiveness of a community-level HIV risk reduction intervention | No | Not cluster-randomised |  |
| Porco et al. | 2019 | Clin Infect Dis | Mass oral azithromycin for childhood mortality: timing of death after distribution in the MORDOR trial | No | Community information-giving or consultation |  |
| Pronyk et al. | 2006 | Lancet | Effect of a structural intervention for the prevention of intimate-partner violence and HIV in rural South Africa: a cluster randomised trial | No | Community collaboration | Microfinance groups participated in trainings by external actors. Groups were collaboratively involved in action planning and implementation by staff at microfinance loan centres for community mobilisation events. |
| Psaki et al. | 2022 | PLOS One | What are we learning about HIV testing in informal settlements in KwaZulu-Natal, South Africa? Results from a randomised controlled trial | No | Not cluster-randomised |  |
| Puffer et al. | 2016 | J Consult Clin Psychol | A church-based intervention for families to promote mental health and prevent HIV among adolescents in rural Kenya: results of a randomised trial | No | Community collaboration | External actors engaged community advisory committees to develop and implement activities. |
| Pullan et al. | 2019 | Lancet | Effects, equity, and cost of school-based and community-wide treatment strategies for soil-transmitted helminths in Kenya: a cluster-randomised controlled trial | No | Community information-giving or consultation |  |
| Quansah et al. | 2022 | PLOS Glob Public Health | The impact of a demand-side sanitation and hygiene promotion intervention on sustained behaviour change and health in Amhara, Ethiopia: a cluster-randomised trial | No | Community collaboration | Community members were oriented to the sanitation and hygiene programme by external actors and formed coordinating committees, which were collaboratively involved in action planning for community mobilisation and commitment events. Community health workers and community conversation facilitators were also trained to promote behaviour change activities. |
| Quattrochi et al. | 2021 | BMJ Glob Health | Effects of a community-driven water, sanitation and hygiene intervention on water and sanitation infrastructure, access, behaviour, and governance: a cluster- | Yes | Community empowerment |  |
|  |  |  | randomised controlled trial in rural Democratic Republic of Congo |  |  |  |
| Rehman et al. | 2019 | Malar J | Intermittent preventive treatment of malaria delivered to primary schoolchildren provided effective individual protection in Jinja, Uganda: secondary outcomes of a cluster-randomised trial (START-IPT) | No | Community information-giving or consultation |  |
| Rey et al. | 1987 | Rev Med Moamb | Schistosomiasis: methods of control in communal villages in Mozambique. 1. Control of molluscs, therapy and community participation | No | Full text not found |  |
| Rhodes et al. | 2020 | Health Educ Res | A peer navigation intervention to prevent HIV among mixed immigrant status Latinx GBMSM and transgender women in the United States: outcomes, perspectives and implications for PrEP uptake | No | Community collaboration | External actors partnered with government agencies, community-based organisations, and community members to develop, implement, and evaluate an HIV programme including peer navigators. |
| Rivera et al. | 2023 | BMC Public Health | Adoption of community-based strategies for sustainable vector control and prevention | No | Community information-giving or consultation |  |
| Rockers et al. | 2023 | PLOS Med | Evaluation of a community health worker home visit intervention to improve child development in South Africa: a cluster-randomised controlled trial | No | No CD outcome reported |  |
| Rojas et al. | 2006 | Biomedica | A multifaceted intervention to prevent American cutaneous leishmaniasis in Colombia: results of a group-randomised trial | No | Full text not found |  |
| Rosato et al. | 2012 | Int Health | Strategies developed and implemented by women's groups to improve mother and infant health and reduce mortality in rural Malawi | Yes | Community empowerment |  |
| Ross et al. | 2007 | AIDS | Biological and behavioural impact of an adolescent sexual health intervention in Tanzania: a community-randomised trial | No | Community information-giving or consultation |  |
| Rotheram-Borus et al. | 2011 | Prev Sci | Philani Plus (+): a Mentor Mother community health worker home visiting programme to improve maternal and infants' outcomes | No | Community information-giving or consultation |  |
| Rubenstein et al. | 2022 | Malar J | A cluster randomised trial of delivery of intermittent preventive treatment of malaria in pregnancy at the community level in Malawi | No | Community information-giving or consultation |  |
| Ruebush et al. | 1994 | Am J Trop Med Hyg | Community participation in malaria surveillance and treatment. III. An evaluation of modifications in the Volunteer Collaborator Network of Guatemala | No | Not cluster-randomised |  |
| Salinger et al. | 2024 | World Dev | People are now working together for a common good: the effect on social capital of participatory design for community-level sanitation infrastructure in urban informal settlements | No | Community information-giving or consultation |  |
| Samburu et al. | 2020 | Int Breastfeed J | Effectiveness of the baby-friendly community initiative in promoting exclusive breastfeeding among HIV negative and positive mothers: a randomised controlled trial in Koibatek sub-county, Baringo, Kenya | No | Community collaboration | Partners, including sub-county health agencies, community health extension workers, and community volunteers, were trained by the Ministry of Health and developed joint plans for implementing the Baby Friendly Communities Initiative. Community health extension workers and community volunteers implemented plans, with supportive supervision by health agencies. |
| San Sebastian et al. | 2001 | Trop Doct | Improving immunisation coverage in rural areas of Ecuador: a cost-effectiveness analysis | No | Not cluster-randomised |  |
| Scarinci et al. | 2020 | Vaccine | HPV vaccine uptake among daughters of Latinx immigrant mothers: findings from a cluster randomised controlled trial of a community-based, culturally relevant intervention | No | Community information-giving or consultation |  |
| Schutte et al. | 2015 | Vaccine | Cost analysis of routine immunisation in Zambia | No | Not cluster-randomised |  |
| Secor et al. | 2020 | Am J Trop Med Hyg | Comparison of school-based and community-wide mass drug administration for schistosomiasis control in an area of western kenya with high initial Schistosoma mansoni infection prevalence: a cluster randomised trial | No | Community information-giving or consultation |  |
| Sevelius et al. | 2022 | J Acquir Immune Defic Syndr | Randomised controlled trial of Healthy Divas: a gender-affirming, peer-delivered intervention to improve HIV care engagement among transgender women living with HIV | No | Not cluster-randomised |  |
| Shargie et al. | 2006 | Bull World Health Organ | Tuberculosis case-finding through a village outreach programme in a rural setting in southern Ethiopia: community randomised trial | No | Community information-giving or consultation |  |
| Shu et al. | 1999 | East Afr Med J | Influence of health education on community participation in rapid assessment of onchocerciasis prior to distribution of ivermectin | No | Full text not found |  |
| Sibanda et al. | 2021 | BMJ Glob Health | Comparison of community-led distribution of HIV self-tests kits with distribution by paid distributors: a cluster randomised trial in rural Zimbabwean communities | Yes | Community empowerment |  |
| Sikkema et al. | 2005 | AIDS | Outcomes of a randomised, controlled community-level HIV prevention intervention for adolescents in low-income housing developments | No | Community collaboration | Adolescents attended sexual health training by external actors and were asked to nominate peer leaders. The peer leadership council routinely met with external actors to plan HIV prevention activities for adolescents and the wider community. |
| Simuyemba et al. | 2023 | Res Sq | An evaluation of the cost of human papilloma virus (HPV) vaccine delivery in Zambia | No | Not cluster-randomised |  |
| Sinanovic et al. | 2003 | Int J Tuberc Lung Dis | Cost and cost-effectiveness of community-based care for tuberculosis in Cape Town, South Africa | No | Not cluster-randomised |  |
| Skull et al. | 2001 | Int J Epidemiol | Should programmes for community-level meningococcal vaccination be considered in Australia? An economic evaluation | No | Not cluster-randomised |  |
| Sohn et al. | 2019 | Public Health Action | Costs and operation management of community outreach programme for tuberculosis in tribal populations in India | No | Not cluster-randomised |  |
| Soofi et al. | 2012 | Lancet | Effectiveness of community case management of severe pneumonia with oral amoxicillin in children aged 2-59 months in Matiari district, rural Pakistan: a cluster-randomised controlled trial | No | Community information-giving or consultation |  |
| Soremekun et al. | 2023 | PLOS Digit Health | Improving outcomes for children with malaria, diarrhoea and pneumonia in Mozambique: a cluster randomised controlled trial of the inSCALE technology innovation | No | Community information-giving or consultation |  |
| Staedke et al. | 2018 | Lancet Glob Health | Assessment of community-level effects of intermittent preventive treatment for malaria in schoolchildren in Jinja, Uganda (START-IPT trial): a cluster-randomised trial | No | Community information-giving or consultation |  |
| Starmann et al. | 2017 | Prev Sci | Exploring couples' processes of change in the context of SASA!, a violence against women and HIV prevention intervention in Uganda | Yes | Community empowerment |  |
| Starmann et al. | 2018 | BMC Public Health | Examining diffusion to understand the how of SASA!, a violence against women and HIV prevention intervention in Uganda | Yes | Community empowerment |  |
| Sudhaker et al. | 2007 | Indian J Community Med | Participatory training programme on prevention of HIV/AIDS, with agent exposure, among Anganwadi workers for training young village women | No | Not research article |  |
| Suraratdecha et al. | 2024 | AIDS Care | Cost-outcome analysis of HIV testing and counseling, linkage and defaulter tracing services in Bukoba, Tanzania | No | Not cluster-randomised |  |
| Sutter et al. | 1983 | Soc Sci Med | Community participation in the control of trachoma in Gazankulu | No | Not cluster-randomised |  |
| Sweat et al. | 2011 | Lancet Infect Dis | Community-based intervention to increase HIV testing and case detection in people aged 16-32 years in Tanzania, Zimbabwe, and Thailand (NIMH Project Accept, HPTN 043): a randomised study | No | Community collaboration | Community working groups and volunteers liaised between external actors and community members, providing input on materials and activities and implementing community engagement around HIV testing and care. |
| Tabana et al. | 2015 | PLOS One | A cost-effectiveness analysis of a home-based HIV counselling and testing intervention versus the standard (facility-based) HIV testing strategy in rural South Africa | No | Community information-giving or consultation |  |
| Tamarozzi et al. | 2012 | Parasit Vectors | Long term impact of large scale community-directed delivery of doxycycline for the treatment of onchocerciasis | No | Not cluster-randomised |  |
| Tang et al. | 2018 | PLOS Med | Crowdsourcing to expand HIV testing among men who have sex with men in China: a closed cohort stepped wedge cluster randomised controlled trial | No | Community collaboration | External actors solicited a contest for concepts and images promoting HIV testing, with entries evaluated by community representatives and multisectoral experts. A regional contest was then held with teams of representatives from government agencies, community-based organisations, and national applicants, with judging done by a multisectoral panel. The final intervention integrated components from national and regional contests and was collaboratively implemented by external actors, government agencies, and community-based organisations. |
| Tang et al. | 2023 | JMIR Public Health Surveill | A pay-it-forward approach to improve chlamydia and gonorrhea testing uptake among female sex workers in China: | No | Community information- |  |
|  |  |  | venue-based superiority cluster randomised controlled trial |  | giving or consultation |  |
| Terris-Prestholt et al. | 2006 | Sex Transm Dis | From trial intervention to scale-up: costs of an adolescent sexual health programme in Mwanza, Tanzania | No | Community information-giving or consultation |  |
| Thang et al. | 2009 | PLOS One | Long-lasting insecticidal hammocks for controlling forest malaria: a community-based trial in a rural area of central Vietnam | No | Community information-giving or consultation |  |
| Thirumurthy et al. | 2021 | Lancet HIV | The effect of providing women sustained access to HIV self-tests on male partner testing, couples testing, and HIV incidence in Kenya: a cluster-randomised trial | No | Community information-giving or consultation |  |
| Thomas et al. | 2016 | Trans R Soc Trop Med Hyg | Effectiveness of TB sensitisation initiatives in improving the involvement of self help group members in rural TB control in south India | No | Community collaboration | Self-help groups decided on the type, format, and timings of tuberculosis sensitisation that they were to receive from external actors. |
| Thomas et al. | 2021 | Lancet Glob Health | Cost and cost-effectiveness of a universal HIV testing and treatment intervention in Zambia and South Africa: evidence and projections from the HPTN 071 (PopART) trial | No | Community information-giving or consultation |  |
| Thomas et al. | 2023 | BMC Infect Dis | Do community measures impact the effectiveness of a community led HIV testing intervention? Secondary analysis of an HIV self-testing intervention in rural communities in Zimbabwe | Yes | Community empowerment |  |
| Tingey et al. | 2015 | AIDS Care | Respecting the circle of life: one year outcomes from a randomised controlled comparison of an HIV risk reduction intervention for American Indian adolescents | No | Community information-giving or consultation |  |
| Tintle et al. | 2021 | Trop Med Health | diarrhoea prevalence in a randomised, controlled prospective trial of point-of-use water filters in homes and schools in the Dominican Republic | No | Community information-giving or consultation |  |
| Tobin et al. | 2011 | Addiction | The STEP into Action study: a peer-based, personal risk network-focused HIV prevention intervention with injection drug users in Baltimore, Maryland | No | Not cluster-randomised |  |
| Torres-Rueda et al. | 2018 | J Acquir Immune Defic Syndr | Cost and cost-effectiveness of a demand creation intervention to increase uptake of voluntary medical male circumcision in Tanzania: spending more to spend less | No | Community information-giving or consultation |  |
| Tripiboon et al. | 2001 | Aust J Prim Health | A HIV/AIDS prevention programme for married women in rural northern Thailand | No | Full text not found |  |
| Trotignon et al. | 2023 | PLOS Negl Trop Dis | Cost of implementing a doxycycline test-and-treat strategy for onchocerciasis elimination among settled and semi-nomadic groups in Cameroon | No | Not cluster-randomised |  |
| Tschampl et al. | 2020 | Int J Infect Dis | Cost-effectiveness of community mobilisation (Camino Verde) for dengue prevention in Nicaragua and Mexico: a cluster randomised controlled trial | Yes | Community empowerment |  |
| Tun-Lin et al. | 2009 | Trop Med Int Health | Reducing costs and operational constraints of dengue vector control by targeting productive breeding places: a multi-country non-inferiority cluster randomised trial | No | Community information-giving or consultation |  |
| Umniyati et al. | 2000 | Dengue Bulletin | Evaluation of community-based Aedes control programme by source reduction in Perumnas Condong Catur, Yogyakarta, Indonesia | No | Not cluster-randomised |  |
| Vanlerberghe et al. | 2009 | BMJ | Community involvement in dengue vector control: cluster randomised trial | No | Community collaboration | Partners, including community leaders, public health workers, and nurses, formed community working groups for dengue control and conducted a situational assessment with communities, identified local needs and priorities, and developed action plans. |
| Vassall et al. | 2014 | PLOS One | Community mobilisation and empowerment interventions as part of HIV prevention for female sex workers in | No | Not cluster-randomised |  |
|  |  |  | Southern India: a cost-effectiveness analysis |  |  |  |
| Vaz Nery et al. | 2019 | Am J Trop Med Hyg | WASH for WORMS: a cluster-randomised controlled trial of the impact of a community integrated water, sanitation, and hygiene and deworming intervention on soil-transmitted helminth infections | No | Community collaboration | Community members participated in 'triggering' meetings by external actors and committed to sanitation improvement. External actors worked with community members to build taps and latrines and promoted handwashing. |
| von Seidlein et al. | 2019 | PLOS Med | The impact of targeted malaria elimination with mass drug administrations on falciparum malaria in Southeast Asia: a cluster randomised trial | No | Community collaboration | External actors partnered with community leaders, community malaria workers, and community volunteers to design and implement community engagement prior to mass drug administration for malaria treatment. |
| Waiswa et al. | 2021 | BMJ Glob Health | Child health and the implementation of community and district-management empowerment for scale-up (CODES) in Uganda: a randomised controlled trial | No | Community collaboration | External actors and district health offices collated and analysed data related to management of local health services, which were then discussed and evaluated by community leaders and members through community score cards. Community representatives and health care workers then developed consensus on prioritised problems and solutions, as actioned through community contracts. Health care workers implemented actions, which were also monitored by community representatives. |
| Walsh et al. | 2020 | Curr HIV/AIDS Rep | Getting to 90-90-90: experiences from the MaxART early access to ART for all (EAAA) trial in eSwatini | No | Full text not found |  |
| Wamae et al. | 2006 | Afr J Health Sci | Community-directed treatment of lymphatic filariasis in Kenya and its role in the national programmes for elimination of lymphatic filariasis | No | Not cluster-randomised |  |
| Wambura et al. | 2017 | AIDS | Increasing voluntary medical male circumcision uptake among adult men in Tanzania | No | Community information-giving or consultation |  |
| Warsame et al. | 2016 | Clin Infect Dis | Pre-referral rectal artesunate treatment by community-based treatment providers in Ghana, Guinea-Bissau, Tanzania, and Uganda (study 18): a cluster-randomised trial | No | Community information-giving or consultation |  |
| Weaver et al. | 2001 | Arch Intern Med | Cost-effectiveness of combined outreach for the pneumococcal and influenza vaccines | No | Not cluster-randomised |  |
| Webel et al. | 2019 | AIDS Care | Randomised clinical trial of a community navigation intervention to improve well-being in persons living with HIV and other co-morbidities | No | Not cluster-randomised |  |
| Weeks et al. | 1997 | AIDS Educ Prev | Does parental involvement make a difference? The impact of parent interactive activities on students in a school-based AIDS prevention programme | No | Community information-giving or consultation |  |
| Willis et al. | 2012 | J Trop Pediatr | Impact of community-based behaviour-change management on perceived neonatal morbidity: a cluster-randomised controlled trial in Shivgarh, Uttar Pradesh, India | No | Community information-giving or consultation |  |
| Wilson et al. | 2019 | Am J Public Health | HIV prevention for black heterosexual men: the barbershop talk with brothers cluster randomised trial | No | Community information-giving or consultation |  |
| Wilson et al. | 2022 | Lancet HIV | Impact of universal testing and treatment on sexual risk behaviour and herpes simplex virus type 2: a prespecified secondary outcomes analysis of the HPTN 071 (PopART) community-randomised trial | No | Community information-giving or consultation |  |
| Winch et al. | 2003 | Trans R Soc Trop Med Hyg | Increases in correct administration of chloroquine in the home and referral of sick children to health facilities through a community-based intervention in Bougouni | No | Community information-giving or consultation |  |
|  |  |  | District, Mali |  |  |  |
| Yapabandara et al. | 2001 | Acta Trop | Control of malaria vectors with the insect growth regulator pyriproxyfen in a gem-mining area in Sri Lanka | No | Community information-giving or consultation |  |
| Younes et al. | 2015 | J Epidemiol Community Health | The effect of participatory women's groups on infant feeding and child health knowledge, behaviour and outcomes in rural Bangladesh: a controlled before-and-after study | No | Not cluster-randomised |  |
| Young et al. | 2015 | Lancet HIV | The HOPE social media intervention for global HIV prevention in Peru: a cluster randomised controlled trial | No | Not cluster-randomised |  |
| Young et al. | 2022 | J Acquir Immune Defic Syndr | A peer-led online community to increase HIV self-testing among African American and Latinx MSM: a randomised controlled trial | No | Not cluster-randomised |  |
| Zewdie et al. | 2023 | AIDS | Uptake of medical male circumcision with household-based testing, and the association of traditional male circumcision and HIV infection | No | Community information-giving or consultation |  |
| Zhang et al. | 2013 | PLOS One | Community based promotion on VCT acceptance among rural migrants in Shanghai, China | No | Not cluster-randomised |  |
| Zhang et al. | 2023 | Nat Med | Pay-it-forward incentives for hepatitis virus testing in men who have sex with men: a cluster randomised trial | No | Community information-giving or consultation |  |
| Zhou et al. | 2003 | Pediatrics | Economic analysis of promotion of hepatitis B vaccinations among Vietnamese-American children and adolescents in Houston and Dallas | No | Not cluster-randomised |  |

CD, communicable disease; NCD, non-communicable disease.
